# High Density Microarray Analysis Reveals New Insights into Genetic Footprints of *Listeria monocytogenes* Strains Involved in Listeriosis Outbreaks

**DOI:** 10.1371/journal.pone.0032896

**Published:** 2012-03-21

**Authors:** Pongpan Laksanalamai, Scott A. Jackson, Mark K. Mammel, Atin R. Datta

**Affiliations:** Office of Applied Research and Safety Assessment, Center for Food Safety and Applied Nutrition, U.S. Food and Drug Administration, Laurel, Maryland, United States of America; Tulane University, United States of America

## Abstract

*Listeria monocytogenes*, a foodborne bacterial pathogen, causes invasive and febrile gastroenteritis forms of listeriosis in humans. Both invasive and febrile gastroenteritis listeriosis is caused mostly by serotypes 1/2a, 1/2b and 4b strains. The outbreak strains of serotype 1/2a and 4b could be further classified into several epidemic clones but the genetic bases for the diverse pathophysiology have been unsuccessful. DNA microarray provides an important tool to scan the entire genome for genetic signatures that may distinguish the *L. monocytogenes* strains belonging to different outbreaks. We have designed a pan-genomic microarray chip (*Listeria* GeneChip) containing sequences from 24 *L. monocytogenes* strains. The chip was designed to identify the presence/absence of genomic sequences, analyze transcription profiles and identify SNPs. Analysis of the genomic profiles of 38 outbreak strains representing 1/2a, 1/2b and 4b serotypes, revealed that the strains formed distinct genetic clusters adhering to their serotypes and epidemic clone types. Although serologically 1/2a and 1/b strains share common antigenic markers microarray analysis revealed that 1/2a strains are further apart from the closely related 1/2b and 4b strains. Within any given serotype and epidemic clone type the febrile gastroenteritis and invasive strains can be further distinguished based on several genetic markers including large numbers of phage genome, and intergenic sequences. Our results showed that the microarray-based data can be an important tool in characterization of *L. monocytogenes* strains involved in both invasive and gastroenteritis outbreaks. The results for the first time showed that the serotypes and epidemic clones are based on extensive pan-genomic variability and the 1/2b and 4bstrains are more closely related to each other than the 1/2a strains. The data also supported the hypothesis that the strains causing these two diverse outbreaks are genotypically different and this finding might be important in understanding the pathophysiology of this organism.

## Introduction

*Listeria monocytogenes* is a Gram-positive foodborne bacterial pathogen responsible for human and animal listeriosis. Recent data [1] indicate that the total number of human listeriosis case in the USA is about 1,600 cases/infections per year causing 255 deaths. The economic burden due to death, hospitalization and destruction of food amounts to several billion dollars each year. The invasive (Inv) form of listeriosis is characterized by septicemia, meningitis, abortion, still birth and death while the febrile gastroenteritis (FG) form is characterized by fever, nausea, vomiting and diarrhea [2], [3]. Invasive listeriosis predominantly affects immuno-compromised individuals including pregnant women, elderly and patients whose immunity is compromised by drug treatment and/or an underlying disease. On the other hand, FG cases have been reported to affect healthy individuals with a high attack rate [2], [3]. Although in recent years several outbreaks of FG outbreaks due to *L. monocytogenes* have been reported [3], [4], the actual burden of FG due to *L. monocytogenes* is not known because FG cases are not routinely screened for *L. monocytogenes* [1].

Based on somatic and flagellar antigens, *L. monocytogenes* strains can be classified into 13 serotypes [5], of which the vast majority of human listeriosis cases are caused by serotypes 1/2a, 1/2b and 4b [2], [6]. The majority of FG outbreaks are caused by serotype 1/2a and 1/2b strains whereas the majority of Inv listeriosis outbreaks are caused by serotype 4b strains [2], [3]. Generally, FG outbreaks have been associated with high level of contamination with *L. monocytogenes* [7] but the relevance of such findings to FG is not clear. In order to understand the genetic and physiological basis of this diverse pathophysiology, several attempts [8]–[10] have been made to identify specific genetic footprints associated with *L. monocytogenes* strains isolated from these outbreaks. Franciosa et al (2001) analyzed a total of 32 strains, 16 from Inv and 16 from FG listeriosis outbreaks by ribotyping, arbitrarily primed PCR (AP-PCR) and interspersed repetitive sequence PCR (IRS-PCR) [9]. Out of these three techniques, only IRS-PCR could group all the FG strains into two specific clusters, distinctly separated from the Inv and a few non-outbreak related strains. This was the first and only indication that there may be distinct genetic markers associated with this diverse group of strains. In a follow-up study, Franciosa et al. (2005) analyzed 27 *L. monocytogenes* serotype 4b and 1/2b strains from Inv and FG listeriosis by several other molecular sub-typing techniques [8]. The restriction fragment length polymorphism (RFLP) of eight different virulence associated genes and pulse-field gel electrophoresis (PFGE) analysis by two different enzymes failed to produce any distinct profile for the Inv and FG strains. These authors also showed no difference in virulence potential among a small numbers of Inv and FG strains when tested by mouse intra-gastric and intra-peritoneal inoculation [8].

Based on several molecular subtyping studies including multi-locus enzyme electrophoresis (MLEE), RFLP and PFGE, genetic structures of *L. monocytogenes* appear to be highly clonal. These molecular subtyping methods revealed that *L. monocytogenes* can be classified into at least three lineages correlated to their serotypes [11], [12]. Further analyses of these *L. monocytogenes* strains associated with different outbreaks using molecular subtyping methods divide these strains into five epidemic clones (ECs), suggesting that strains causing major outbreaks are genetically related [11], [13], [14] (Table S1). To date, 4 ECs including ECI, ECII, ECIV and ECV belong to serotype 4b, which are implicated in most documented human listeriosis cases whereas ECIII isolates are serotype 1/2a. ECIV, previously assigned as ECIa, is closely related to ECI isolates [11]. ECV isolates harbor unique genetic markers distinct enough to be assigned as another separate clone, although they are similar to ECII isolates [11].

With the advent of whole genome sequencing technology and the availability of advanced bioinformatics tools, it is possible to identify small changes in the genetic makeup of bacterial pathogens, including *L. monocytogenes* [15], [16]. These developments were instrumental in identifying differences in genetic sequences and lead to the development of serotype, ECs and lineage specific molecular detection techniques [17], [18]. Whole genome sequencing was also useful in exploring genetic diversity [12], [19], characterizing outbreak strains and aiding epidemiological investigations [20]. An alternative to whole genome sequencing and analysis which is costly and time consuming, the DNA microarray-based analysis has been successfully used to probe entire genomes of *L. monocytogenes* [21]–[24]. The array-based analysis has been useful in species identification [21], [23], virulence assessment [16], serotype and lineage determination [24], [25] and during epidemiological investigations [20], [22] as the pan-genomic variability is supposed to provide much better discriminatory power than PFGE, multiple loci variable tandem repeats analysis (MLVA) and multi loci sequence typing (MLST), which depend on the variability in limited areas of the genome. In this work we describe the design of a pan-genomic microarray chip for *L. monocytogenes* based on the publicly available information (as of May 2009) of 24 *L. monocytogenes* genome sequences. Using our custom *Listeria* GeneChip (Affymetrix technology), we analyzed 38 *L. monocytogenes* strains isolated from Inv and FG listeriosis outbreaks. The strains represent serotypes 1/2a, 1/2b and 4b including epidemiologically matched clinical and food isolates. Our results show that the microarray-based analysis using this GeneChip can be used as an outbreak investigation tool to identify genome differences and separate *L. monocytogenes* strains based on their serotype, epidemic clone type and outbreaks. The distinct difference in genetic footprints between strains of FG and Inv outbreaks may help in understanding the diverse pathophysiology of this organism.

## Materials and Methods

### *L. monocytogenes* strains and preparation of genomic DNA for hybridization

Strains of *L. monocytogenes* were obtained from various sources (Table S1) and stored in our facility at -80°C in brain heart infusion (BHI) broth containing 20% glycerol. The cultures were routinely grown in BHI broth and/or BHI agar at 37°C. Genomic DNA was isolated from 10ml of cultures grown overnight in a shaking incubator at 170rpm using the Qiagen DNeasy Blood and Tissue kit (Qiagen, Valencia, CA) with the following modifications. The cultures were resuspended in 180 µl lysis buffer and incubated at 37°C for 1 hour, followed by addition of buffer AL with 25 µl proteinase K supplied with the kit. The reaction mixture was then used to extract genomic DNA following incubation at 55°C for another 1 hour. The extracted genomic DNA was further purified and concentrated using Microcon YM-30 microcentrifuge filter (Millipore, Billerica, MA) to a final volume of approximately 20 µl. 10 µg of the genomic DNA was fragmented by incubating at 37°C for 10 minutes in a 40 µl reaction volume containing 1X One-Phore-All buffer (GE Healthcare, Waukesha, WI) and 0.2 units DNaseI (Promega, Madison, WI), followed by heat-inactivation at 95°C for 10 minutes. The fragmented genomic DNA was then labeled on its 3′ end by 2 nM biotin-11-ddATP using 60 units of terminal transferase (Promega, Madison, WI). Labeling was carried out at 37°C for 4 hours and the labeled product was used for hybridization onto the GeneChip.

**Table 1:** *Listeria monocytogenes* genome sequences used in the *Listeria* GeneChip design.

Strain	Serotype	Source	Description
*L. monocytogenes* 10403S	1/2a	Broad Institute	Streptomycin resistant derivative of strain 10403
*L. monocytogenes* J2818	1/2a	Broad Institute	Food isolate, Listeriosis outbreak in 2000 related to consumption of turkey
*L. monocytogenes* F6900	1/2a	Broad Institute	A single case of human listeriosis in 1989 related to consumption of processed meat
*L. monocytogenes* J0161 (FSL R2-499)	1/2a	Broad Institute	Listeriosis outbreak in 2000 related to consumption of turkey
*L. monocytogenes* FSL N3-165	1/2a	Broad Institute	Soil isolate
*L. monocytogenes* FSL J2-003	1/2a	Broad Institute	Feces/farm isolate
*L. monocytogenes* FSL F2-515	1/2a	Broad Institute	Food isolate, rarely cause human disease
*L. monocytogenes* EGD-e	1/2a	EC Consortium	Derivative of EGD
*L. monocytogenes* F6854	1/2a	J. Craig Venter Institute	Associated with turkey hotdog, sporadic case in Oklahoma in 1988
*L. monocytogenes* Finland1998	1/2a group^1^	Broad Institute	Finland 1988
*L. monocytogenes* FSL R2-503 (G6054)	1/2b	Broad Institute	Gastroenteritis outbreak in the USA in 1994
*L. monocytogenes* FSL J1-194	1/2b	Broad Institute	Sporadic human listeriosis
*L. monocytogenes* FSL J1-175	1/2b	Broad Institute	Water isolate, not associated with any disease
*L. monocytogenes* J2-064	1/2b	Broad Institute	Food isolate, commonly cause human disease
*L. monocytogenes* LO28	1/2c	Broad Institute	Widely distributed and used in virulence study
*L. monocytogenes* FSL R2-561	1/2c	Broad Institute	Human isolate, sporadic case
*L. monocytogenes* FSL J1-208	4a	Broad Institute	Animal clinical isolate, first serotype 4a being sequenced
*L. monocytogenes* HCC23	4a	Mississippi State University	Channel catfish isolate
*L. monocytogenes* HPB2262	4b	Broad Institute	Gastroenteritis in Northern Italy in 1997
*L. monocytogenes* FSL N1-017	4b	Broad Institute	Trout in brine, not associated with any human cases
*L. monocytogenes* F2365	4b	J. Craig Venter Institute	Associated with cheese product, California outbreak in 1985
*L. monocytogenes* H7858	4b	J. Craig Venter Institute	Associated with hot dog, Multiple state Outbreak in 1998–1999
*L. monocytogenes* Clip81459	4b	Institute Pasteur	Epidemic isolate from a patient in France in 1999
*L. monocytogenes* FSL J2-071	4c	Broad Institute	Associated with animal disease

1 The serotype was determined by BLAST analysis of the sequence with the serotype specific primers as reported by Doumith et al, 2004 [17]

### Array hybridization, washing, staining and scanning

Hybridizations were performed according to the Affymetrix GeneChip Expression Analysis Technical Manual (http://media.affymetrix.com/support/downloads/manuals/expression_analysis_technical_manual.pdf). Briefly, 200µl hybridization reactions containing 10µg of labeled fragmented DNA, 100mM MES, 1M(Na^+^), 20mM EDTA, 0.01% Tween-20, 50pM control oligoB2 (Affymetrix, Santa Clara, CA), 0.1 mg/ml herring sperm DNA (Promega), 7.8% dimethylsulfoxide (DMSO) (Sigma, St. Louis, MO), were heated at 95°C for 1 minute followed by incubation at 45°C for 5 minutes, prior to hybridizing onto the Affymetrix *Listeria* GeneChip at 45°C with rotation (60rpm) for 16 hours in a hybridization oven. The buffer preparation, the wash and staining procedures were carried out on an Affymetrix FS-450 fluidics station using the mini_prok2v1_450 fluidics script as described by GeneChip Expression Analysis Technical Manual with the slight modification that Streptavidin solution mix was replaced with Streptavidin, R-phycoerythrin conjugate (SAPE) (Invitrogen, San Diego, CA). Arrays were subsequently scanned using a GeneChip Scanner 3000 7G with GCOS v1.4 software.

### *Listeria* GeneChip design

The *L. monocytogenes* microarray (*Listeria* GeneChip) is custom designed using Affymetrix chip technology and has components for use as an expression or genotyping array and probes for use as a tiling array. The *Listeria* expression/genotyping microarray was designed to represent 64,539 annotated gene sequences from 24 sequenced strains of *L. monocytogenes* which were available from GenBank and Broad Institute (http://www.broadinstitute.org/annotation/genome/listeria_group/MultiHome.html) as of May, 2009 and 7,354 intergenic sequences from four of the sequenced *L. monocytogenes* strains (F2365, HCC23, EGD-e, and Clip81459) (Table 1). Identical or nearly identical alleles of a gene from different genomes were represented with one probe set while each divergent allele was represented by an additional probe set. The expression array consists of 253,361 25-mer oligonucleotides that represent a total of 18,630 probe sets including 45 AFFY controls, 4,481 intergenic regions and 14,104 genes. Each probe set contains approximately 28 oligonucleotide probes; up to 14 perfect match (PM) probes and 14 mismatch probes (MM). Mismatch probes are identical to the perfect match probe with the exception of a one nucleotide (nt) mismatch located at the 13th (middle) position of the oligo nucleotide sequence.

The tiling portion of the array consists of 568,677 probes covering the whole genome of AE017262 4b F2365. The 25-mer probes cover the genome at 4-nt gaps between starts, allowing for 20-nt overlap between probes. Each nucleotide in the genome is covered by 5 probes for detection of Single Nucleotide Polymorphisms (SNPs) relative to the reference sequence.

### Parsing CEL files, probe set summarization methods and data analysis tools

All Affymetrix CEL files generated in this study were parsed and analyzed using algorithms including MAS5.0 [26]–[28] for gene detection calls and Robust Multi Array (RMA) methods for summarized probe-set intensities implemented by the Affy package of R and Bioconductor [29]–[32].

### Genomic relationship analysis

Affymetrix MAS5.0 algorithm using Affy package of R and Bioconductor was used to identify *L. monocytogenes* gene contents for which the presence or absence of genes were coded as T (present) or A (absent), respectively. The gene present/absent binary nucleotide calls were concatenated for each strain, such that a 18,630 bp sequence was generated to represent the gene content. Genes that are not phylogenetically informative [33] as they are either present or absent in all of the tested strains were eliminated from the analysis. The parsimonious informative sites were identified from the concatenated gene content sequences of each strain using Splitstree 4.11.3 [34]. A neighbor-net or neighbor joining phylogeny highlighting the distribution of *L. monocytogenes* serotype 1/2a, 1/2b and 4b was constructed using the uncorrected *p*-distance in Splitstree 4.11.3.

**Figure 1:**
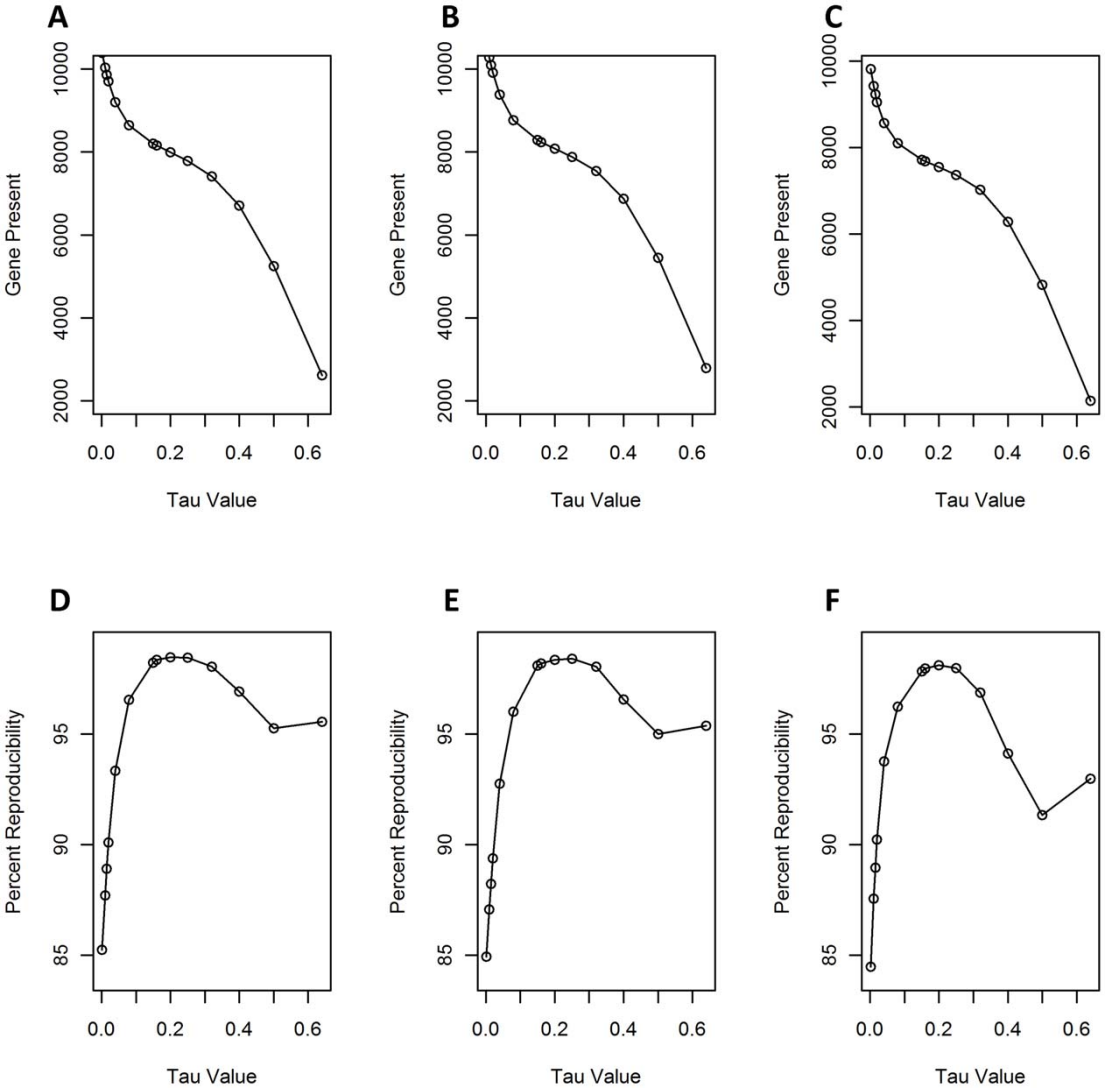
Effects of *Tau* values on gene present calls (LS402; A, LS406; B and LS411; C) and percent reproducibility (LS402; D, LS406; E and LS411; F).

## Results and Discussion

### Accurate gene detection and validity of the *Listeria* GeneChip

The availability of *L. monocytogenes* genome sequences allowed us to design a GeneChip that integrates sequences from many genomes in one single GeneChip. In this study, we investigated the *L. monocytogenes* genome diversity using Affymetrix high-density microarray GeneChip that was custom designed based on 24 *L. monocytogenes* genome sequences available at the time of GeneChip design (Table 1) .The *Listeria* GeneChip was designed to study genome diversity and gene expression as well as single nucleotide polymorphisms (SNPs) of *L. monocytogenes*. All probe-sets on Our *Listeria* GeneChip consist of up to 14 probe-pairs per gene, phage gene and intergenic region. Each probe-pair contains one perfect-match probe (PM) and another with one nucleotide mismatch (MM). This feature enables us to perform highly accurate gene content detection.

To determine gene contents (present/absent) in *L. monocytogenes* strains, MAS5.0 gene detection approach was used [26]–[28]. However, MAS5.0 requires several factors that need to be empirically determined to obtain the most accurate gene present or absent calls. First, by performing hybridization experiments of genomic DNA on the GeneChip, target-specific intensity differences relative to its overall hybridization for each probe pair can be measured providing the discrimination score (R), defined as R = {(PM-MM)/(PM+MM)}. This value is then used to generate *p*-values. In addition to the R Score, sensitivity and/or specificity of gene detection depends on a small positive threshold value, *Tau* [27], [28] which needs to be adjusted to make the most accurate gene present/absent calls. The last step is the determination of the detection *p*-value using a one-sided Wilcoxon Signed Rank test as described by Jackson et al. [28]. Probe-sets with the detection *p*-value <0.05 were scored as present and ≥0.05 as absent. Fig. 1 A–C shows the effects of *Tau* values on gene present calls for strains LS402, LS406 and LS411. Increased *Tau* values clearly resulted in reduced numbers of gene present calls that correspond to false negatives. However, higher *Tau* values will also result in reduced numbers of truly present genes (false-negative). In addition, it is important to note that our *Listeria* GeneChip was designed based on the available genome sequences to study global genomic diversity. Several probe sets may contain probes that share different percent identities from the same genes in various genomes. The predicted numbers of present genes, therefore, can or often do exceed the true numbers of genes in the *L. monocytogenes* genomes, providing better resolution for gene detection (Fig. 1 A–C and Table S1).

Data reproducibility, defined as a ratio between the numbers of probe-sets that are either absent or present in all of the triplicate experiments and the total numbers of probe-sets, was determined to assess the reliability of the genotyping results. However, when gene detection analysis was performed using MAS5.0 algorithm by applying various *Tau* values to the individual CEL files in the triplicate experiments, we found that the data reproducibility changed, depending largely on the *Tau* value selection. Figures 1 (D–F) indicated that reduced *Tau* values provided results with lower percent reproducibility. *Tau* values up to 0.3, on the other hand, increased the percent reproducibility to approximately 98%. While increasing *Tau* values above 0.3 resulted in reduced percent reproducibility, *Tau* values above 0.5 again raised the percent reproducibility. This increase in reproducibility resulted from more false absent calls occurring with *Tau* values above 0.5, (Fig. 1D–F). Based on these studies, among all three triplicate experiments for strains LS402, LS406 and LS411, *Tau* values between 0.2 and 0.3 appear to provide the most accurate gene detection calls for our Listeria GeneChip.

**Figure 2:**
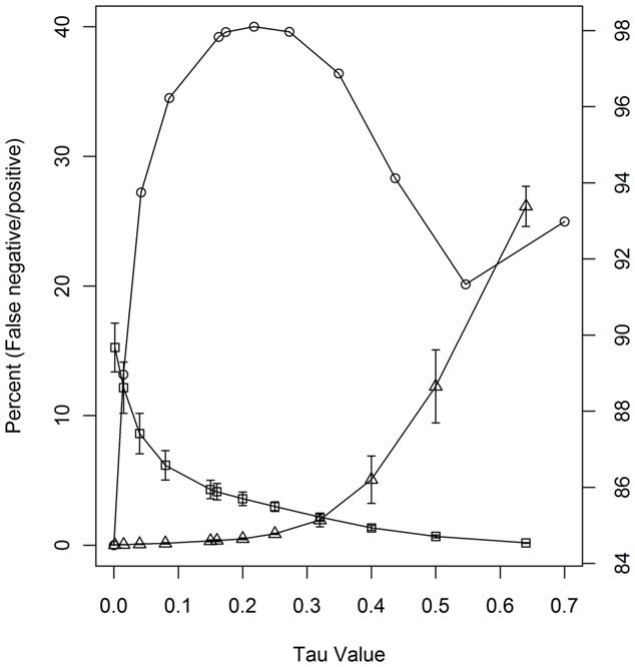
Effects of *Tau* values on percent false negative and positive calls based on the hybridization results of strain LS411. Left axis indicates percent false positive (□) and false negative (Δ) Right axis indicates percent reproducibility (○) from the three LS411 hybridization results.

To further validate our *Listeria* microarray, we determined the false positive and false negative numbers based on hybridization experiments. Since the *Listeria* GeneChip was designed mainly from the F2365 genome (LS411, Table S1) [19], these numbers can be determined by comparing the hybridization results of the LS411 strain with the *in silico* BLAST results of every individual probe on the array against the F2365 genome sequence. Hence, genes that are absent from the hybridization but present in the BLAST results are referred to as false negative whereas the reverse is a false positive. We generated the numbers of gene absent/present calls using varied *Tau* values from the LS411 strain hybridization results. *In silico* BLAST analysis of probe sequence against the F2365 genome returned 8,038 probe-sets of which at least one probe is 100% matched to the F2365 genome (referred to as a 100% matched probe). As a result, 10,592 out of 18,630 total probe-sets were automatically scored as absent. Although 8,038 probe-sets may be called as present, we found that 6,980 probe-sets can be scored as present based on the following two criteria. First, each probe-set must contain at least 40% of 100% matched probes compared to the total probe numbers. Secondly, each probe-set screened by the first criteria must contain at least six 100% matched probes, allowing at least 150 nucleotides to be detected. The numbers of nucleotides are therefore sufficient for gene detection. The gene detection calls from the triplicate LS411 hybridization experiments using varied *Tau* values were then compared with the gene present/absent calls from the BLAST analysis to identify the numbers of false positive and false negative. As expected, with increasing *Tau* values, the numbers of false negative rose due to more absent calls, whereas the numbers of false positive dropped exponentially (Fig. 2). Furthermore, a specific searching within the LMOf2365 probe-sets revealed that *Tau* values below 0.3 provide less than 1% of false negative calls (data not shown). This analysis strongly indicated that *Tau* values between 0.2 and 0.3 provide the most accurate gene detection calls for the *Listeria* GeneChip hence the *Tau* value of 0.25 was subsequently used in the downstream analyses to identify the gene contents of different *L. monocytogenes* strains. A similar study using Affymetrix GeneChip® *E. coli* Genome 2.0 revealed that a *Tau* value of 0.2 provided the most accurate gene present/absent calls [28].

### Validation of the data by a Robust Multi-array Averaging (RMA) analysis

**Figure 3:**
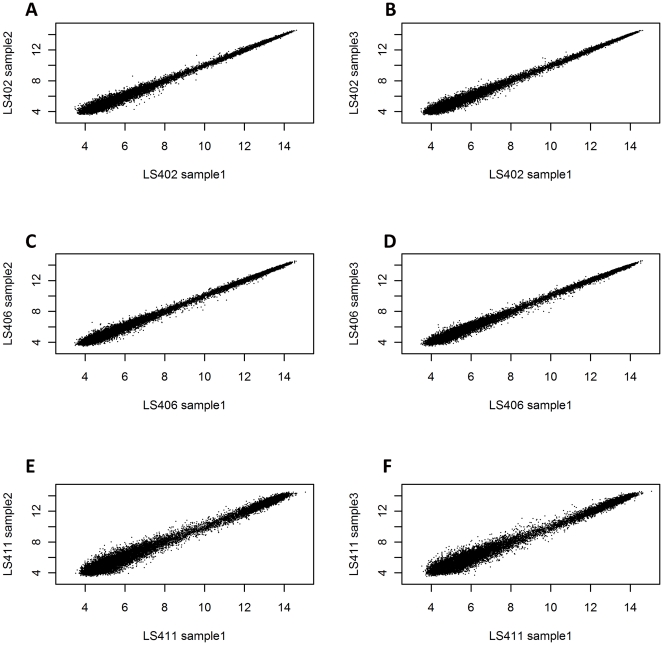
Scatter plots of the summarized Robust Multi-array Averaging (RMA) intensities from the triplicate experiments of strains LS402 (A, B), LS406 (C, D), and LS411 (E, F).

**Figure 4:**
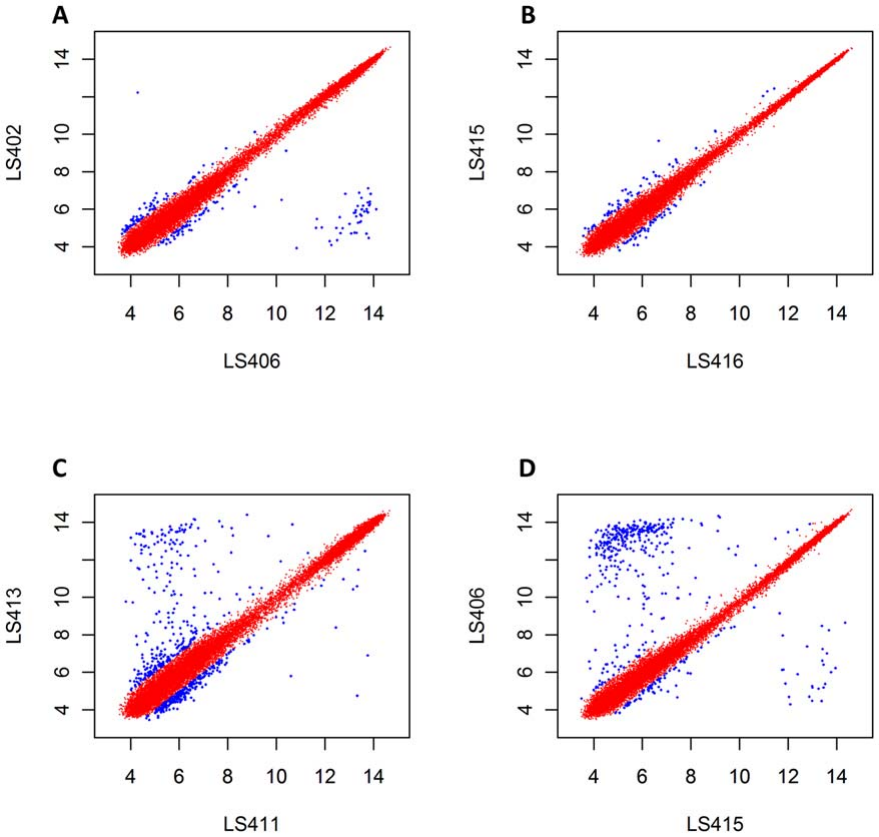
Comparison of the summarized Robust Multi-array Averaging (RMA) intensities by scatter plots. Between strains from the same epidemic clones (ECIV) and pathotypes; FG, LS402 and LS406 (A) and Inv; LS415 and LS416 (B). Between strains from the same epidemic clones (ECI) and pathotype (Inv), different outbreaks; LS411 and LS413 (C). Between strains from the same epidemic clones (ECIV), different pathotype; Inv, LS415 and FG, LS406 (D). Red dots indicate summarized RMA intensity differences of less than or equal to 2-fold between two strains. Blue dots indicate RMA intensity differences of more than 2-fold between two strains.

The Robust Multi-array Averaging (RMA) approach [29] was also used to validate our *Listeria* GeneChip by comparing summarized probe-set intensities, independent to MAS5.0 algorithm. In contrast to MAS5.0 gene present/absent call analysis, MM probes are not considered as a part of the RMA calculation. The summarized probe-set intensities were therefore determined based on PM probes alone. As a part of the result validation, the same CEL files used in the MAS5.0 analysis from the triplicate hybridization experiments performed in some strains were subjected to RMA analysis using Affy package in R-Bioconductor. The summarized probe intensities among triplicate experiments with LS402, LS406 and LS411 were compared. The RMA scatter plots between the samples in triplicate (Fig. 3 A–F) revealed that the summarized probe-set intensities are comparable and there are no significantly different probe-set intensities among all of the probe-sets. Comparison of the summarized probe-set intensities between the strains derived from the same epidemic clones (ECIV) and pathotype (Fig. 4A and B) by scatter plots reveals the close similarity between the two strains. However by comparing summarized probe-sets intensity that have more than one unit difference from the same EC strains derived from the different outbreaks, there are 264 probe-sets in the same pathotype strains (LS411 and LS413, Fig. 4C) whereas 452 probe-sets were found in the different pathotype strains (LS406 and LS415, Fig 4D). This result suggested that, within the same epidemic clones, the outcomes of the diseases may be affected by the genetic information. It is important to note that although the numbers of probe-sets exceed the true gene numbers as previously described (Fig. 1 A–C), the advantage of probe-set redundancy in our *Listeria* GeneChip is improved resolution as shown in Fig. 4 and Table S1. The results suggested that within the same epidemic clone, there is a higher genomic diversity between strains belonging to different pathotypes (4C and 4D) than between strains of the same pathotypes obtained from the diverse outbreaks. However, whether these genetic variations are responsible for different pathotypes cannot be ascertained at this time.

**Figure 5:**
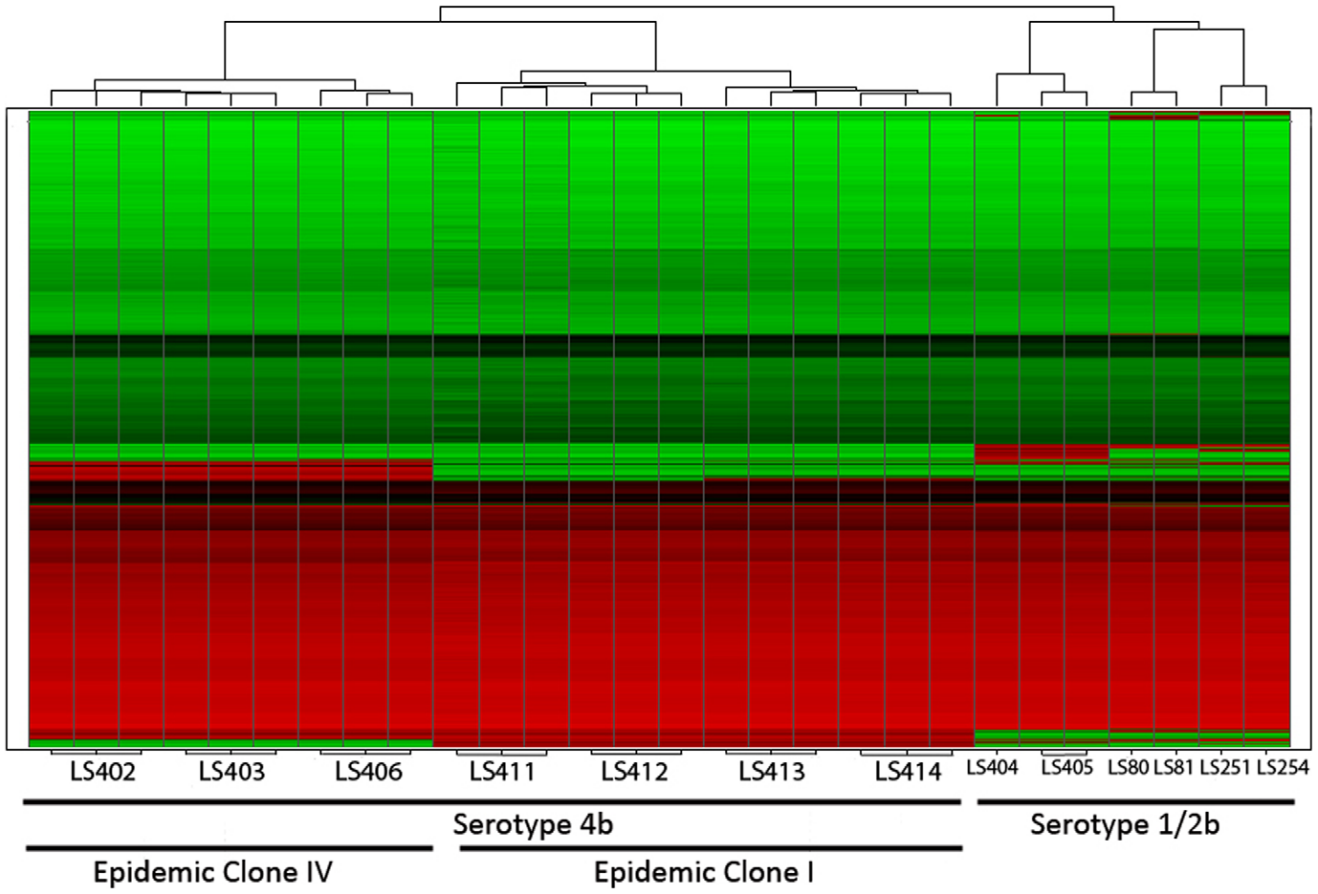
Hierarchical clustering dendrogram and heat map analysis based on the summarized Robust Multi-array Averaging (RMA) intensities obtained from all of the strains using in this study. The RMA summarized probe-set intensities are ranging from 2.5 (green) to 14 (red).

We further validated the microarray results using the RMA-summarized probe intensities generated from serotype 4b (ECI and ECIV) and serotype 1/2b strains. A heat map generated from the RMA-summarized probe intensities shows that, when the individual 4b strains were examined in triplicate, the results appeared to be identical suggesting consistency in the array data (Fig. 5). The dendrogram calculated from probe intensities using a Euclidean means hierarchical clustering analysis groups the seven serotype 4b *L. monocytogenes* strains into 2 clusters correlating to their ECs (ECI and IV) while they are clearly distinct from the serotype 1/2b strains. The heat map reveals distinct trends in the differences of gene content among strains from different ECs as also established by previous study in *E. coli* [28]. Interestingly, matched food and clinical isolates from the same outbreaks also show slight differences in the RMA-summarized probe intensities suggesting that some adaptation within the strains derived from related sources may occur in response to different environments.

### Genomic relationship of *L. monocytogenes* strains

**Figure 6:**
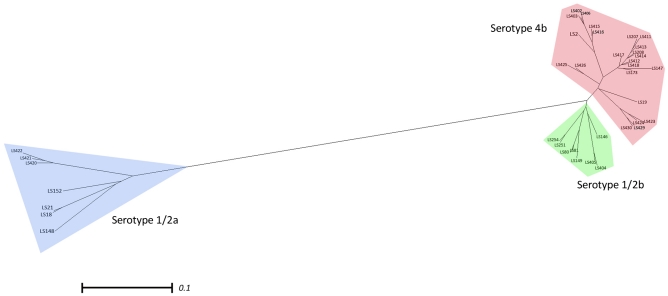
Relatedness analysis of the compatible parsimony informative genes from the 38 strains of *L. monocytogenes*. The tree was generated from the concatenated gene contents using neighbor joining with the uncorrected *p* distance. The colors indicated the serotype of *L. monocytogenes* strains (red; serotype 4b, green; serotype 1/2b and blue; serotype 1/2a). Scale bar represents number of gene differences (present or absent) per gene site.

The investigation of genome diversity using pan-genome analysis has been reported in several prokaryotic species [12]. To understand the relatedness and global diversity of *L. monocytogenes* strains, genomic content information (present/absent) was analyzed using MAS5.0 algorithm to infer strain relatedness. All CEL files generated from the genomic DNA hybridization of the 38 *L. monocytogenes* strains from the three serotypes (1/2a, 1/2b and 4b) were parsed and analyzed using Affy package in R-Bioconductor with the *Tau* value of 0.25. There are 18,360 probe-sets on the array, of which 8,079 probe-sets are conserved (either present or absent) across all 38 strains (44% of all probe-sets), likely representing a large component of the core *L. monocytogenes* genome. As a result, they are not phylogenetically informative and were consequently excluded from the analysis [33]. Resulting pan-genome of 10,551 phylogenetically informative sites (present/absent), including 2,326 intergenic regions and 925 phage genes were concatenated and then used to determine the relatedness of the 38 strains of *L. monocytogenes*. The 9,767 parsimoniously informative sites were selected from the 10,551 phylogenetically informative probe-sets. A neighbor-joining tree of the 38 strains constructed using the gene content information, separated the strains into their respective serotypes and epidemic clones (Fig. 6). Serotype 1/2a strains are more divergent than serotype 1/2b and 4b strains. Previous study using Multilocus Enzyme Electrophoresis (MEE) also identified 30 electrophoretic types (ET) within the 1/2a strains and only 10 and 11 ETs were found in serotypes 4b and 1/2b strains, respectively [35], [36]. Our microarray analysis revealed that the serotype 1/2a strains exhibit 2,090 unique probe-sets whereas 93 and 18 unique probe-sets are found in serotype 1/2b and 4b strains, respectively (Tables S2, S3, S4). These results, therefore, confirmed the divergence among these serotypes and agree with several other molecular subtyping studies [11].

**Figure 7:**
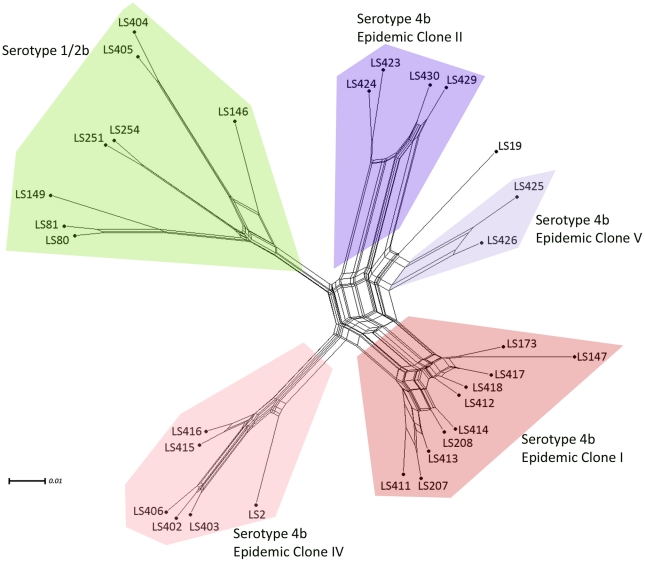
A neighbor-net constructed from the gene contents from 31 strains belonging to the two serotypes 1/2b and 4b. The parallel edges represent incompatible signals indicative of independent gene loss or gain due to the multiple transductions or recombinations. Serotypes and epidemic clones are grouped in different color as indicated. Node labels refer to strain names (Listed in Table S1). Scale bar represents number of gene differences (present or absent) per gene site.

The concatenated sequences of the probe-sets (present/absent) were also examined using Neighbor-net in Splitstree program [34], [37]. Since the serotype 1/2b and 4b strains represented most of the strains in the two listeriosis outbreak pathotypes and are also more closely related, the corresponding taxa (31 of 38), based on the concatenated gene contents, were characterized separately [38]. The neighbor-net method was used to infer the strain relatedness between the two serotypes, 1/2b and 4b, and revealed a network like phylogeny (Fig. 7) where the parallel edges represent incompatible signals indicative of independent gene loss or gain due to the multiple transductions or recombinations [33], [39]. The neighbor-net based parallelogram divided the 31 *L. monocytogenes* strains into two distinct groups mirroring their serotypes (1/2b and 4b). The serotype 4b strains were divided into four distinct clusters corresponding to their ECs (ECI, ECII, ECIV and ECV). Interestingly, the parallelogram analysis revealed that there may be more substantial mutations or recombinations in ECI, ECII and ECV than those of the ECIV and serotype 1/2b strains. Pairwise homoplasy index (PHI) [40], which tests for recombination events, was conducted in Splitstree providing a *p*-value of 0.0 which confirms that there was significant evidence of recombination or parallel gene gain/loss due to multiple transduction events. In addition, when 925 phage genes were removed from the analysis, the topology and clusters of the resulting tree were unaffected (data not shown) suggesting the stability of the genomes and the relationship among these *L. monocytogenes* strains has not been influenced by phages.

Currently, serotype 4b strains are divided into four ECs in which each of them are harboring unique probe-sets (Tables S5, S6, S7, S8). Our microarray result confirmed that one of the probe-sets, representing gene LMOf2365_0687, which does not cluster with other ORFs in the *L. monocytogenes* genomes, is unique in 10 strains belonging to the ECI [11]. ECII strains used in this study including the 2002 deli meat outbreak (LS429 and LS430) and the 1998–1999 hotdog outbreak (LS423 and LS424) strains are clustered closely together. Our microarray analysis revealed that three probe-sets, representing genes LMOh7858_1168, LMOh7858_2753 and LMOh7858_2764 from the H7858 genome, are unique to ECII [41] but are absent in all other strains. The 2000 North Carolina Mexican-style cheese outbreak strains (LS425 and LS426) were branched away from the ECII due to some genetic variation, forming an ECV cluster [11]. Tables S5, S6, S7, S8 show the present probe-sets unique to each EC. As a result, the microarray analyses, substantiated by their agreement with several molecular characterization studies [11], confirm both characteristics and relationship among epidemic clones.

Previous study involving the whole genome comparison between the serotypes 1/2a and 4b strains revealed that *L. monocytogenes* genomes are very similar (syntenic) and most of the differences are due to phage genomes and transposable elements as well as SNPs [19]. In addition, high similarity of the gene contents with relatively small numbers of specific genes found in serotype 1/2a and 4b strains derived from different epidemiologic backgrounds suggested that *L. monocytogenes* may not require many genetic elements to adapt to different environments and exhibit different virulence attributes as suggested by Nelson et al.[19]. We have analyzed the genetic contents of 31 strains, 7 from FG and 24 from Inv outbreaks, belonging to serotype 1/2b and 4b. No unique probe-sets were found to be associated either with the FG or with the Inv strains. However, comparison of different pathotypes under each serotype revealed some unique sequences present in these strains (Tables S9, S10, S11, S12). For instance, within the ECIV group consisting of 3 FG and 3 Inv strains, the FG strains (LS402, LS403 and LS406) harbor 193 unique sequences of which 151(78%), accounted for phage related genes. Further comparison between these serotype 4b FG (LS402, LS403 and LS406) and 24 serotype 4b Inv strains revealed that these strains have 63 unique probe-sets in the FG strains of which 45 probe-sets (71%), accounted for phage-related genes. On the other hand, in the serotype 1/2b cluster, 58 probe-sets were identified to be unique among the FG strains (LS404, LS405, LS251 and LS254) of which 6 probe-sets are phage-related (Table S10). The importance of *comK* prophage in *L. monocytogenes* for niche-specific adaptation, biofilm formation and persistence has been recently demonstrated [42]. Our finding may indicate that small number of changes may be crucial to account for pathophysiology of human listeriosis. It is also possible that the whole genome architecture taken account of phage genes, intergenic regions and SNPs may be more important in determining the ecology and pathophysiology of *L. monocytogenes*.

Based on the genomic profiles of 31 different strains representing serotypes 1/2b and 4b (ECII, ECIV and ECV), both FG and Inv listeriosis strains from matched pairs of food and clinical isolates are more similar closely related than to those from the different outbreaks (Fig 7). However, relatedness analysis of the serotype 4b (ECI) strains showed that the majority of these strains are grouped by sources of the strains (Fig 7). Interestingly, some variations revealed from the gene content analysis between the clinical and food isolates derived from the same outbreaks do occur. We found that approximately 2% and 4% of probe-sets numbers are different between the food and clinical isolates from the 1981 coleslaw (LS413 and LS414) and 1985 Jalisco cheese outbreaks (LS411 and LS412), respectively. In addition, when the food and clinical isolates from both these outbreaks were compared, 19 probe-sets were exclusively present in the food isolates whereas 94 probe-sets were present in the clinical isolates (data not shown). These results suggested that the small variation in the food and clinical isolates may be due to microevolution [43] resulting from adaptation to host or food environments of these ECI strains. Further study with a larger number of strains may elucidate this point.

In conclusion, we report the design of a microarray GeneChip consisting of 24 *L. monocytogenes* genomes from the public databases and genomic analysis of *L. monocytogenes* outbreak strains using this GeneChip. Gene detection methods using the MAS5.0 algorithm to identify gene presence and absence have been optimized for our GeneChip based on known sequences. The numbers of present genes called may exceed the true numbers of genes in *L. monocytogenes* genomes due to redundancy of probes within probe-sets as a result of our microarray design in order to study global diversity of *L. monocytogenes* genomes. We showed that the results obtained from either RMA or MAS5.0 approaches are consistent, suggesting the reliability and validity of the data. The gene content analysis using the phylogenetically informative sites revealed that *L. monocytogenes* strains are divided into three distinct groups correlating with the serotypes. Strains belonging to the serotype 1/2a are more genetically distant from those of 1/2b and 4b strains. Within the same serotype, strains that belong to the same ECs are clustered closely together. Comparison of the serotype 4b, ECIV FG and Inv strains indicated that the majority of the uniquely present probe-sets in FG isolates are phage-related genes suggesting that phages may play a significant role in the divergence of these two pathotypes and may play important roles in pathotype determination. We showed that our high density microarray can identify genetic contents that are specific to serotypes, pathotypes and epidemic clones. Our results also indicated that microarray based genotypic analysis can be a very important tool in outbreak investigation as closely related members of the same serotype as well as the food and clinical isolates derived from outbreaks can further be differentiated from each other.

## Supporting Information

Table S1 *Listeria monocytogenes* strains used in this study.
(DOCX)

Table S2 Probe-sets uniquely present in the serotype 1/2a strains.
(DOCX)

Table S3 Probe-sets uniquely present in the serotype 1/2b strains.
(DOCX)

Table S4 Probe-sets uniquely present in the serotype 4b strains.
(DOCX)

Table S5 Probe-sets uniquely present in the serotype 4b, epidemic clone I.
(DOCX)

Table S6 Probe-sets uniquely present in the serotype 4b, epidemic clone II.
(DOCX)

Table S7 Probe-sets uniquely present in the serotype 4b, epidemic clone IV.
(DOCX)

Table S8 Probe-sets uniquely present in the serotype 4b, epidemic clone V.
(DOCX)

Table S9 Probe-sets uniquely present in the serotype 1/2b strains that cause invasive listeriosis.
(DOCX)

Table S10 Probe-sets uniquely present in the serotype 1/2b strains that cause febrile gastroenteritis listeriosis.
(DOCX)

Table S11 Probe-sets uniquely present in the serotype 4b, ECIV strains that cause invasive listeriosis.
(DOCX)

Table S12 Probe-sets uniquely present in the serotype 4b, ECIV strains that cause febrile gastroenteritis listeriosis.
(DOCX)

## References

[1] Scallan E, Hoekstra RM, Angulo FJ, Tauxe RV, Widdowson MA (2011). Foodborne illness acquired in the United States – major pathogens.. Emerg Infect Dis.

[2] Datta AR, Miliotis MD, Bier JW (2003). “*Listeria monocytogenes*”.. International handbook of foodborne pathogens.

[3] Norton DM, Braden CR, Ryser ET, Marth EH (2007). Foodborne listeriosis.. Listeria, listeriosis and food safety.

[4] Ooi ST, Lorber B (2005). Gastroenteritis due to *Listeria monocytogenes*.. Clin Infect Dis.

[5] Seeliger HP, Hohne K (2011). Serotyping of *Listeria monocytogenes* and related species.. Methods Microbiol.

[6] Graves LM, Swaminathan B, Hunter SB, Ryser ET, Marth EH (2007). Subtyping *Listeria monocytogenes*.. Listeria, listeriosis, and food safety.

[7] Barbuddhe SB, Chakraborty T (2009). *Listeria* as an enteroinvasive gastrointestinal pathogen.. Curr Top Microbiol Immunol.

[8] Franciosa G, Maugliani A, Floridi F, Aureli P (2005). Molecular and experimental virulence of *Listeria monocytogenes* strains isolated from cases with invasive listeriosis and febrile gastroenteritis.. FEMS Immunol Med Microbiol.

[9] Franciosa G, Tartaro S, Wedell-Neergaard C, Aureli P (2001). Characterization of *Listeria monocytogenes* strains involved in invasive and noninvasive listeriosis outbreaks by PCR-based fingerprinting techniques.. Appl Environ Microbiol.

[10] Miettinen MK, Siitonen A, Heiskanen P, Haajanen H, Bjorkroth KJ (1999). Molecular epidemiology of an outbreak of febrile gastroenteritis caused by *Listeria monocytogenes* in cold-smoked rainbow trout.. J Clin Microbiol.

[11] Cheng Y, Siletzky RM, Kathariou S, Dongyou L (2008). Genomic division/lineages, epidemic clones and population structure.. Handbook of *Listeria monocytogenes*.

[12] Deng X, Phillippy AM, Li Z, Salzberg SL, Zhang W (2010). Probing the pan-genome of *Listeria monocytogenes*: new insights into intraspecific niche expansion and genomic diversification.. BMC Genomics.

[13] Piffaretti JC, Kressebuch H, Aeschbacher M, Bille J, Bannerman E (1989). Genetic characterization of clones of the bacterium *Listeria monocytogenes* causing epidemic disease.. Proc Natl Acad Sci U S A.

[14] Chen Y, Knabel SJ (2008). Prophages in *Listeria monocytogenes* contain single-nucleotide polymorphisms that differentiate outbreak clones within epidemic clones.. J Clin Microbiol.

[15] Glaser P, Frangeul L, Buchrieser C, Rusniok C, Amend A (2001). Comparative genomics of *Listeria* species.. Science.

[16] Doumith M, Cazalet C, Simoes N, Frangeul L, Jacquet C (2004). New aspects regarding evolution and virulence of *Listeria monocytogenes* revealed by comparative genomics and DNA arrays.. Infect Immun.

[17] Doumith M, Buchrieser C, Glaser P, Jacquet C, Martin P (2004). Differentiation of the major *Listeria monocytogenes* serovars by multiplex PCR.. J Clin Microbiol.

[18] Ward TJ, Gorski L, Borucki MK, Mandrell RE, Hutchins J (2004). Intraspecific phylogeny and lineage group identification based on the *prf*A virulence gene cluster of *Listeria monocytogenes*.. J Bacteriol.

[19] Nelson KE, Fouts DE, Mongodin EF, Ravel J, DeBoy RT (2004). Whole genome comparisons of serotype 4b and 1/2a strains of the food-borne pathogen *Listeria monocytogenes* reveal new insights into the core genome components of this species.. Nucleic Acids Res.

[20] Orsi RH, Borowsky ML, Lauer P, Young SK, Nusbaum C (2008). Short-term genome evolution of *Listeria monocytogenes* in a non-controlled environment.. BMC Genomics.

[21] Call DR, Borucki MK, Loge FJ (2003). Detection of bacterial pathogens in environmental samples using DNA microarrays.. J Microbiol Methods.

[22] Borucki MK, Kim SH, Call DR, Smole SC, Pagotto F (2004). Selective discrimination of *Listeria monocytogenes* epidemic strains by a mixed-genome DNA microarray compared to discrimination by pulsed-field gel electrophoresis, ribotyping, and multilocus sequence typing.. J Clin Microbiol.

[23] Volokhov D, Rasooly A, Chumakov K, Chizhikov V (2002). Identification of *Listeria* species by microarray-based assay.. J Clin Microbiol.

[24] Zhang C, Zhang M, Ju J, Nietfeldt J, Wise J (2003). Genome diversification in phylogenetic lineages I and II of *Listeria monocytogenes*: identification of segments unique to lineage II populations.. J Bacteriol.

[25] Call DR, Borucki MK, Besser TE (2003). Mixed-genome microarrays reveal multiple serotype and lineage-specific differences among strains of *Listeria monocytogenes*.. J Clin Microbiol.

[26] Hubbell E, Liu WM, Mei R (2002). Robust estimators for expression analysis.. Bioinformatics.

[27] Affymetrix (2002). http://media.affymetrix.com/support/technical/whitepapers/sadd_whitepaper.pdf.

[28] Jackson SA, Patel IR, Barnaba T, LeClerc JE, Cebula TA (2011). Investigating the global genomic diversity of Escherichia coli using a multi-genome DNA microarray platform with novel gene prediction strategies.. BMC Genomics.

[29] Bolstad BM, Irizarry RA, Astrand M, Speed TP (2003). A comparison of normalization methods for high density oligonucleotide array data based on variance and bias.. Bioinformatics.

[30] Irizarry RA, Bolstad BM, Collin F, Cope LM, Hobbs B (2003). Summaries of Affymetrix GeneChip probe level data.. Nucleic Acids Res.

[31] Gentleman RC, Carey VJ, Bates DM, Bolstad B, Dettling M (2004). Bioconductor: open software development for computational biology and bioinformatics.. Genome Biol.

[32] Irizarry RA, Wu Z, Jaffee HA (2006). Comparison of Affymetrix GeneChip expression measures.. Bioinformatics.

[33] Abu-Ali GS, Lacher DW, Wick LM, Qi W, Whittam TS (2009). Genomic diversity of pathogenic *Escherichia coli* of the EHEC 2 clonal complex.. BMC Genomics.

[34] Huson DH, Bryant D (2006). Application of phylogenetic networks in evolutionary studies.. Mol Biol Evol.

[35] Kathariou S (2002). *Listeria monocytogenes* virulence and pathogenicity, a food safety perspective.. J Food Prot.

[36] Bibb WF, Schwartz B, Gellin BG, Plikaytis BD, Weaver RE (1989). Analysis of *Listeria monocytogenes* by multilocus enzyme electrophoresis and application of the method to epidemiologic investigations.. Int J Food Microbiol.

[37] Huson DH, Kloepper TH (2005). Computing recombination networks from binary sequences.. Bioinformatics.

[38] Bandelt HJ, Dress AW (1992). Split decomposition: a new and useful approach to phylogenetic analysis of distance data.. Mol Phylogenet Evol.

[39] Huson DH (1998). SplitsTree: analyzing and visualizing evolutionary data.. Bioinformatics.

[40] Bruen TC, Philippe H, Bryant D (2006). A simple and robust statistical test for detecting the presence of recombination.. Genetics.

[41] Cheng Y, Kim JW, Lee S, Siletzky RM, Kathariou S (2010). DNA probes for unambiguous identification of *Listeria monocytogenes* epidemic clone II strains.. Appl Environ Microbiol.

[42] Verghese B, Lok M, Wen J, Alessandria V, Chen Y (2011). *comK* prophage junction fragments as markers for *Listeria monocytogenes* genotypes unique to individual meat and poultry processing plants and a model for rapid niche-specific adaptation, biofilm formation, and persistence.. Appl Environ Microbiol.

[43] Gilmour MW, Graham M, Van DG, Tyler S, Kent H (2010). High-throughput genome sequencing of two *Listeria monocytogenes* clinical isolates during a large foodborne outbreak.. BMC Genomics.

