# Supplementary material for: High Density Microarray Analysis Reveals New Insights into Genetic Footprints of Listeria monocytogenes Strains Involved in Listeriosis Outbreaks
Source: PLoS One. 2012 Mar 21;7(3):e32896. doi: 10.1371/journal.pone.0032896 (PMC3310058; doi:10.1371/journal.pone.0032896)
Supplement: Table S1 — Listeria monocytogenes strains used in this study. (DOCX) [file pone.0032896.s002.docx]

**Table S1 *Listeria monocytogenes* strains used in this study.**

| **Strain** | **Serotype** | **Source** | **Outbreak/Episode** | **Outbreak Type** | **Number of present**  **calls^1^** | **Reference** |
| --- | --- | --- | --- | --- | --- | --- |
| LS2 | 4b | Patient | 1983 milk outbreak Massachusetts | Invasive | 7,744 | ScottA |
| LS18 | 1/2a | Food (raw milk) | 1983 raw milk outbreak | Invasive | 8,024 | V-7 |
| LS19 | 4b | Food (raw milk) | 1983 raw milk outbreak | Invasive | 7,633 | V37LE |
| LS21 | 1/2a | n/k | 1983 raw milk outbreak | Invasive | 7,952 | V-7 |
| LS80 | 1/2b | Patient | 1987 New York, ricotta cheese, Sporadic | Invasive | 7,670 | CDC G76 |
| LS81 | 1/2b | Food (ricotta cheese) | 1987 New York, ricotta cheese, Sporadic | Invasive | 7,668 | CDC G136 |
| LS146 | 1/2b | Patient (placenta) | NK | Invasive | 7,584 | CDC G1125 |
| LS147 | 4b | Patient (blood) | NK | Invasive | 7,869 | CDC G1124 |
| LS148 | 1/2a | Patient | NK | Invasive | 8,260 | CDC G1070 |
| LS149 | 1/2b | Patient (spinal fluid) | NK | Invasive | 7,799 | CDC G1049 |
| LS152 | 1/2a | Patient (blood) | NK | Invasive | 7,948 | CDC G553 |
| LS173 | 4b | Patient | NK | NK | 7,727 | NFPA N7295 |
| LS207 | 4b | Food (coleslaw) | 1990 Regional coleslaw outbreak | Invasive | 7,530 | FDA HPB #2 |
| LS208 | 4b | Patient | 1990 Regional coleslaw outbreak | Invasive | 7,727 | FDA HPB #37 |
| LS251 | 1/2b | Patient (blood) | 1993 Italian rice salad outbreak | Febrile Gastroenteritis | 7,541 | CDC G4598 |
| LS254 | 1/2b | Food (cheese pastry) | 1993 Italian rice salad outbreak | Febrile Gastroenteritis | 7,640 | CDC G4601 |
| LS402 | 4b | Food (corn salad) | Italian corn salad outbreak | Febrile Gastroenteritis | 7,839 | CDC H7013 |
| LS403 | 4b | Patient (blood) | Italian corn salad outbreak | Febrile Gastroenteritis | 7,867 | CDC H7014 |
| LS404 | 1/2b | Food (chocolate milk) | Chocolate milk outbreak | Febrile Gastroenteritis | 7,856 | CDC G6003 |
| LS405 | 1/2b | Patient | Chocolate milk outbreak | Febrile Gastroenteritis | 7,740 | CDC G6054 |
| LS406 | 4b | Patient (stool) | Italian corn salad outbreak | Febrile Gastroenteritis | 7,915 | CDC H7015/HPB2262 |

**Table S1 (continued): *Listeria monocytogenes* strains used in this study.**

| **Strain** | **Serotype** | **Source** | **Outbreak/Episode** | **Outbreak type** | **Numbers of present**  **calls^1^** | **References** |
| --- | --- | --- | --- | --- | --- | --- |
| LS411 | 4b | Food | 1985 Los Angeles epidemic | Invasive | 7,328 | FSL-J1-110/F2365 |
| LS412 | 4b | Patient | 1985 Los Angeles epidemic | Invasive | 7,641 | FSL-J1-119 |
| LS413 | 4b | Food | 1981 Halifax epidemic | Invasive | 7,619 | FSL-N3-008 |
| LS414 | 4b | Patient | 1981 Halifax epidemic | Invasive | 7,711 | FSL-J1-108 |
| LS415 | 4b | Food | 1988-1990 UK epidemic | Invasive | 7,671 | FSL-N3-013 |
| LS416 | 4b | Patient | 1988-1990 UK epidemic | Invasive | 7,693 | FSL-J1-116 |
| LS417 | 4b | Food | 1987 Switzerland epidemic | Invasive | 7,620 | FSL-N3-022 |
| LS418 | 4b | Patient | 1987 Switzerland epidemic | Invasive | 7,616 | FSL-J1-123 |
| LS420 | 1/2a | Food | 1989 US hot dog, Sporadic | Invasive | 8,168 | FSL-N3-031 |
| LS421 | 1/2a | Patient | 1989 US hot dog, Sporadic | Invasive | 8,142 | FSL-J1-101/F6854 |
| LS422 | 1/2a | Patient | 2000 US sliced turkey epidemic | Invasive | 8,216 | FSL-R2-499 |
| LS423 | 4b | Food | 1998-1999 US hot dog epidemic | Invasive | 7,868 | FSL-N1-227 |
| LS424 | 4b | Patient | 1998-1999 US epidemic | Invasive | 7,839 | FSL-N1-225 |
| LS425 | 4b | Food | 2000 North Carolina epidemic | Invasive | 7,537 | FSL-R2-500 |
| LS426 | 4b | Patient | 2000 North Carolina epidemic | Invasive | 7,572 | FSL-R2-501 |
| LS429 | 4b | Patient | 2002 NE USA epidemic | Invasive | 7,726 | FSL-R2-763 |
| LS430 | 4b | Food | 2002 NE USA epidemic | Invasive | 7,780 | FSL-R2-764 |

^1^ Numbers of present calls calculated using MAS5.0 with the *Tau* value of 0.25. NK; not known.
