# Supplementary material for: High Density Microarray Analysis Reveals New Insights into Genetic Footprints of Listeria monocytogenes Strains Involved in Listeriosis Outbreaks
Source: PLoS One. 2012 Mar 21;7(3):e32896. doi: 10.1371/journal.pone.0032896 (PMC3310058; doi:10.1371/journal.pone.0032896)
Supplement: Table S2 — Probe-sets uniquely present in the serotype 1/2a strains. (DOCX) [file pone.0032896.s001.docx]

**Supporting Information Table S2: Probe-sets uniquely present in the serotype 1/2a strains**

| **Probe ID** | **Annotation** |
| --- | --- |
| AARI_0004_s_at | 99% similar to lmo0171 |
| AARI_0005_s_at | 99% similar to lmo0160 |
| AARI_0032_s_at | 99% similar to lmo0613 |
| AARI_0039_s_at | NK |
| AARI_0047_s_at | 99% similar to lmo2539 |
| AARI_0057_s_at | 99% similar to lmo0333 |
| AARI_0077_s_at | 100% similar to lmo0204 |
| AARI_0079_s_at | 100% similar to lmo2784 |
| AARI_0094_s_at | 99% similar to lmo0785 |
| AARI_0098_s_at | 98% similar to lmo0626 |
| AARI_0109_s_at | 99% similar to lmo2073 |
| AARI_0120_x_at | 98% similar to lmo2251 |
| AARI_0122_s_at | 99% similar to lmo0974 |
| AARI_0131_s_at | 99% similar to lmo0171 |
| AARI_0132_at | 98% similar to lmo0171 |
| AARI_0133_s_at | 99% similar to lmo0170 |
| AARI_0140_s_at | 99% similar to lmo2461 |
| AARI_0146_s_at | 99% similar to lmo2179 |
| AARI_0147_s_at | 99% similar to lmo2179 |
| AARI_0154_s_at | 99% similar to lmo0434 |
| AARI_0175_s_at | 100% similar to lmo2750 |
| AARI_0189_s_at | 99% similar to lmo1755 |
| AARI_0191_s_at | 99% similar to lmo1543 |
| AARI_0193_x_at | 99% similar to lmo0454 |
| AARI_0201_s_at | 99% similar to lmo1843 |
| AARI_0202_s_at | 99% similar to lmo2576 |
| AARI_0203_at | 100% similar to lmo2576 |
| AARI_0206_s_at | 99% similar to lmo0202 |
| AARI_0215_s_at | 100% similar to lmo0429 |
| AARI_0218_s_at | 99% similar to lmo2820 |
| AARI_0222_s_at | 99% similar to lmo0627 |
| AARI_0226_s_at | 100% similar to lmo2786 |
| AARI_0234_at | 100% similar to lmo2601 |
| AARI_0242_s_at | 99% similar to lmo0001 |
| AARI_0254_s_at | 99% similar to lmo2355 |
| AARI_0294_s_at | 99% similar to lmo2120 |
| AARI_0295_s_at | 100% similar to lmo2121 |
| AARI_0308_s_at | 99% similar to lmo0845 |
| AARI_0310_s_at | 100% similar to lmo1820 |
| AARI_0336_s_at | 100% similar to lmo0653 |
| AARI_0339_s_at | 99% similar to lmo0412 |
| AARI_0340_x_at | NK |
| AARI_0343_at | 99% similar to lmo0444 |
| AARI_0358_s_at | 99% similar to lmo1593 |
| AARI_0360_s_at | 99% similar to lmo1320 |
| AARI_0364_x_at | NK |
| AARI_0371_s_at | 99% similar to lmo2337 |
| AARI_0375_at | NK |
| AARI_0385_s_at | 99% similar to lmo2079 |
| AARI_0394_s_at | 99% similar to lmo0835 |
| AARI_0403_s_at | 99% similar to lmo1076 |
| AARI_0408_x_at | 99% similar to lmo1446 |
| AARI_0409_s_at | 98% similar to lmo1644 |
| AARI_0411_s_at | 98% similar to lmo2747 |
| AARI_0415_s_at | 99% similar to lmo2569 |
| AARI_0427_s_at | NK |
| AARI_0428_s_at | 99% similar to lmo2092 |
| AARI_0432_s_at | 100% similar to lmo0641 |
| AARI_0434_s_at | 100% similar to LMHCC_2059 |
| AARI_0438_s_at | 100% similar to lmo2500 |
| AARI_0439_s_at | 99% similar to lmo0292 |
| AARI_0441_s_at | 99% similar to lmo2419 |
| AARI_0446_s_at | 99% similar to lmo1003 |
| AARI_0465_s_at | 99% similar to lmo0627 |
| AARI_0470_s_at | 99% similar to lmo2845 |
| AARI_0472_s_at | 99% similar to lmo0411 |
| AARI_0476_at | 100% similar to lmo1145 |
| AARI_0481_s_at | 99% similar to lmo2338 |
| AARI_0486_s_at | 99% similar to lmo0595 |
| AARI_0487_s_at | 99% similar to lmo1605 |
| AARI_0494_s_at | 99% similar to lmo2011 |
| AARI_0504_s_at | 99% similar to lmo2237 |
| AARI_0507_s_at | 99% similar to lmo0872 |
| AARI_0514_s_at | 99% similar to lmo2154 |
| AARI_0520_s_at | 100% similar to lmo1679 |
| AARI_0528_s_at | 99% similar to lmo1132 |
| AARI_0530_s_at | 99% similar to lmo0650 |
| AARI_0533_x_at | 98% similar to lmo0355 |
| AARI_0536_at | 99% similar to lmo2691 |
| AARI_0537_s_at | 100% similar to lmo0659 |
| AARI_0539_x_at | 100% similar to lmo0321 |
| AARI_0540_s_at | 99% similar to lmo2770 |
| AARI_0541_s_at | 99% similar to lmo2489 |
| AARI_0545_at | 99% similar to lmo1825 |
| AARI_0560_s_at | NK |
| AARI_0562_s_at | 99% similar to lmo1641 |
| AARI_0565_s_at | 99% similar to lmo1363 |
| AARI_0568_s_at | 99% similar to lmo0035 |
| AARI_0573_s_at | 99% similar to lmo1567 |
| AARI_0574_s_at | 99% similar to lmo1222 |
| AARI_0580_s_at | 99% similar to lmo1828 |
| AARI_0585_at | NK |
| AARI_0587_s_at | 99% similar to lmo1988 |
| AARI_0590_s_at | 99% similar to lmo1835 |
| AARI_0591_s_at | 98% similar to lmo1131 |
| AARI_0596_s_at | 100% similar to lmo0734 |
| AARI_0597_s_at | NK |
| AARI_0598_s_at | 98% similar to lmo2267 |
| AARI_0600_at | NK |
| AARI_0620_s_at | 98% similar to lmo2014 |
| AARI_0627_s_at | NK |
| AARI_0633_s_at | 100% similar to lmo1393 |
| AARI_0638_s_at | 99% similar to lmo1693 |
| AARI_0648_s_at | 99% similar to lmo0594 |
| AARI_0652_s_at | NK |
| AARI_0663_s_at | 99% similar to lmo1728 |
| AARI_0668_at | 99% similar to lmo1152 |
| AARI_0681_at | 100% similar to lmo1946 |
| AARI_0684_s_at | 100% similar to lmo1283 |
| AARI_0686_s_at | 99% similar to lmo1751 |
| AARL_0096_s_at | NK |
| AARL_0236_at | NK |
| AARL_0291_s_at | 98% similar to LMHCC_1967 |
| AARL_0389_x_at | NK |
| AARL_0505_x_at | 98% similar to LMHCC_0976 |
| AARL_0548_x_at | 98% similar to LMHCC_0335 |
| AARL_0665_s_at | NK |
| AARL_0736_s_at | 100% similar to LMHCC_2091 |
| AARL_0759_s_at | 98% similar to LMHCC_0583 |
| AARL_0828_s_at | NK |
| AARL_0894_s_at | 98% similar to LMHCC_1042 |
| AARM_0058_s_at | 99% similar to lmo2785 |
| AARM_0070_s_at | 100% similar to lmo2770 |
| AARM_0085_s_at | 99% similar to lmo0052 |
| AARM_0116_s_at | NK |
| AARM_0173_s_at | 99% similar to lmo2467 |
| AARM_0188_x_at | 99% similar to lmo0546 |
| AARM_0205_s_at | 100% similar to lmo0344 |
| AARM_0235_s_at | 100% similar to lmo0348 |
| AARM_0262_s_at | 99% similar to lmo0006 |
| AARM_0295_s_at | 99% similar to lmo2138 |
| AARM_0303_s_at | 98% similar to lmo2757 |
| AARM_0313_s_at | 99% similar to lmo0038 |
| AARM_0314_s_at | 99% similar to lmo0037 |
| AARM_0319_s_at | 99% similar to lmo0033 |
| AARM_0364_s_at | 99% similar to lmo0644 |
| AARM_0365_s_at | 99% similar to lmo0644 |
| AARM_0395_s_at | 98% similar to lmo2851 |
| AARM_0409_s_at | 100% similar to lmo0600 |
| AARM_0419_s_at | 99% similar to lmo0590 |
| AARM_0448_s_at | 99% similar to lmo2520 |
| AARM_0459_s_at | 99% similar to lmo0630 |
| AARM_0480_s_at | 99% similar to lmo0092 |
| AARM_0497_x_at | 99% similar to lmo2559 |
| AARM_0504_s_at | 99% similar to lmo1833 |
| AARM_0532_s_at | 99% similar to lmo0372 |
| AARM_0534_s_at | 98% similar to lmo0371 |
| AARM_0562_s_at | 100% similar to lmo1226 |
| AARM_0564_s_at | 99% similar to lmo1226 |
| AARM_0569_s_at | 99% similar to lmo2489 |
| AARM_0575_s_at | 99% similar to lmo2221 |
| AARM_0589_x_at | 98% similar to lmo1374 |
| AARM_0591_s_at | 99% similar to lmo1376 |
| AARM_0596_s_at | 99% similar to lmo0402 |
| AARM_0636_s_at | 98% similar to lmo0569 |
| AARM_0699_s_at | 99% similar to lmo1512 |
| AARM_0719_s_at | NK |
| AARM_0740_x_at | 100% similar to lmo1770 |
| AARM_0745_s_at | 100% similar to lmo0734 |
| AARM_0752_s_at | 99% similar to lmo0086 |
| AARM_0753_s_at | 99% similar to lmo0086 |
| AARM_0779_s_at | 99% similar to lmo1935 |
| AARM_0786_s_at | 99% similar to lmo0701 |
| AARM_0787_s_at | 99% similar to lmo0701 |
| AARM_0790_s_at | 99% similar to lmo0705 |
| AARM_0803_s_at | 99% similar to lmo1223 |
| AARM_0823_s_at | 98% similar to lmo1728 |
| AARM_0835_s_at | 99% similar to lmo0961 |
| AARM_0866_s_at | 99% similar to lmo2267 |
| AARM_0867_s_at | 99% similar to lmo2267 |
| AARM_0874_s_at | 99% similar to lmo0759 |
| AARM_0883_s_at | 98% similar to lmo1354 |
| AARM_0886_x_at | 98% similar to lmo1357 |
| AARM_0907_s_at | 100% similar to lmo0233 |
| AARM_0917_s_at | 99% similar to lmo2576 |
| AARM_0920_s_at | NK |
| AARM_0929_s_at | 99% similar to lmo0157 |
| AARM_0941_s_at | 100% similar to lmo1287 |
| AARM_0957_s_at | 99% similar to lmo1886 |
| AARM_0990_s_at | 99% similar to lmo2441 |
| AARM_1009_s_at | 99% similar to lmo1565 |
| AARM_1034_s_at | 99% similar to lmo1393 |
| AARM_1041_s_at | 99% similar to lmo1021 |
| AARM_1055_s_at | 99% similar to lmo2095 |
| AARM_1077_s_at | 98% similar to lmo1292 |
| AARM_1081_s_at | 100% similar to lmo2262 |
| AARM_1100_s_at | 100% similar to lmo1610 |
| AARM_1125_s_at | 100% similar to lmo1838 |
| AARM_1130_x_at | 99% similar to lmo1919 |
| AARM_1144_s_at | 99% similar to lmo2359 |
| AARM_1145_s_at | 99% similar to lmo1899 |
| AARM_1147_s_at | 99% similar to lmo1915 |
| AARM_1149_s_at | 98% similar to lmo1916 |
| AARM_1194_s_at | 98% similar to lmo1473 |
| AARM_1211_s_at | NK |
| AARM_1227_s_at | 99% similar to lmo2085 |
| AARM_1244_x_at | 99% similar to lmo0955 |
| AARM_1262_s_at | 99% similar to lmo1368 |
| AARM_1264_x_at | 100% similar to lmo1130 |
| AARM_1269_x_at | 99% similar to lmo1370 |
| AARM_1274_s_at | 99% similar to lmo0906 |
| AARM_1281_s_at | 99% similar to lmo2388 |
| AARM_1296_s_at | 99% similar to lmo1721 |
| AARM_1307_s_at | 99% similar to lmo1173 |
| AARM_1311_at | 99% similar to lmo2074 |
| AARM_1321_s_at | 99% similar to lmo1741 |
| AARM_1330_at | 99% similar to lmo1971 |
| AARM_1330_s_at | 99% similar to lmo1971 |
| AARM_1331_s_at | 99% similar to lmo1970 |
| AARM_1362_s_at | 99% similar to lmo0982 |
| AARM_1374_x_at | NK |
| AARM_1400_s_at | 99% similar to lmo1076 |
| AARM_1404_x_at | 99% similar to lmo2067 |
| AARM_1411_s_at | 99% similar to lmo1641 |
| AARM_1413_s_at | 98% similar to lmo2444 |
| AARM_1427_s_at | 100% similar to lmo1384 |
| AARM_1428_at | NK |
| AARM_1433_s_at | 99% similar to lmo0727 |
| AARM_1434_s_at | NK |
| AARM_1448_x_at | 99% similar to lmo0532 |
| AARM_1457_s_at | NK |
| AARM_1489_s_at | 100% similar to lmo1804 |
| AARM_1494_s_at | 99% similar to lmo1490 |
| AARM_1500_s_at | 99% similar to lmo0830 |
| AARM_1511_s_at | 99% similar to lmo1485 |
| AARM_1515_x_at | 99% similar to lmo0826 |
| AARM_1538_s_at | 99% similar to lmo1286 |
| AARM_1545_s_at | NK |
| AARM_1552_s_at | 99% similar to lmo0934 |
| AARM_1565_s_at | 99% similar to lmo2105 |
| AARM_1567_s_at | 99% similar to lmo1305 |
| AARM_1569_s_at | 99% similar to lmo0003 |
| AARM_1598_s_at | 99% similar to lmo0567 |
| AARM_1602_s_at | 99% similar to lmo0555 |
| AARM_1606_s_at | 100% similar to lmo2044 |
| AARM_1610_s_at | 100% similar to lmo0722 |
| AARM_1616_s_at | 98% similar to lmo0530 |
| AARM_1617_s_at | 99% similar to lmo2660 |
| AARM_1619_s_at | NK |
| AARM_1621_at | 99% similar to lmo1587 |
| AARM_1621_s_at | 99% similar to lmo1587 |
| AARM_1632_s_at | 100% similar to lmo1910 |
| AARM_1658_s_at | 98% similar to lmo1987 |
| AARM_1679_s_at | NK |
| AARM_1682_s_at | 99% similar to lmo0525 |
| AARM_1693_s_at | 99% similar to lmo1361 |
| AARM_1695_s_at | NK |
| AARM_1701_s_at | 99% similar to lmo1386 |
| AARM_1712_s_at | NK |
| AARM_1724_s_at | 99% similar to lmo1881 |
| AARM_1726_s_at | NK |
| AARM_1728_s_at | 100% similar to lmo1684 |
| AARM_1732_s_at | 99% similar to lmo1777 |
| AARM_1734_s_at | 99% similar to lmo1422 |
| AARM_1742_s_at | 100% similar to lmo1548 |
| AARM_1743_s_at | 99% similar to lmo1000 |
| AARM_1749_s_at | 100% similar to lmo2232 |
| AARM_1759_s_at | 99% similar to lmo2505 |
| AARM_1765_s_at | 99% similar to lmo1161 |
| AARM_1768_at | 99% similar to lmo0514 |
| AARM_1768_x_at | 99% similar to lmo0514 |
| AARY_0012_s_at | 100% similar to lmo2467 |
| AARY_0023_x_at | 99% similar to lmo2688 |
| AARY_0024_s_at | 99% similar to lmo0086 |
| AARY_0035_s_at | 99% similar to lmo0109 |
| AARY_0071_s_at | 99% similar to lmo2006 |
| AARY_0087_s_at | 100% similar to lmo2121 |
| AARY_0088_s_at | 99% similar to lmo2121 |
| AARY_0094_s_at | 99% similar to lmo2126 |
| AARY_0113_s_at | 100% similar to lmo1822 |
| AARY_0119_s_at | 99% similar to lmo2488 |
| AARY_0152_s_at | 99% similar to lmo0927 |
| AARY_0152_x_at | 99% similar to lmo0927 |
| AARY_0198_s_at | 99% similar to lmo0588 |
| AARY_0201_s_at | 99% similar to lmo1759 |
| AARY_0212_s_at | 99% similar to lmo2079 |
| AARY_0214_at | 99% similar to lmo2085 |
| AARY_0235_s_at | 99% similar to lmo2843 |
| AARY_0244_s_at | 100% similar to lmo0919 |
| AARY_0246_s_at | 99% similar to lmo0918 |
| AARY_0247_s_at | 99% similar to lmo0918 |
| AARY_0253_x_at | 100% similar to lmo1886 |
| AARY_0254_x_at | 100% similar to lmo1886 |
| AARY_0267_s_at | 100% similar to lmo0681 |
| AARY_0276_at | 100% similar to lmo1307 |
| AARY_0277_s_at | 99% similar to lmo1307 |
| AARY_0283_s_at | 99% similar to lmo2050 |
| AARY_0286_s_at | 100% similar to lmo0019 |
| AARY_0301_s_at | 100% similar to lmo1072 |
| AARY_0344_s_at | 99% similar to lmo1947 |
| AARY_0345_s_at | 99% similar to lmo2513 |
| AARY_0381_s_at | 100% similar to lmo1060 |
| AARY_0396_s_at | 100% similar to lmo2143 |
| AARY_0441_s_at | 100% similar to lmo0702 |
| AARY_0481_s_at | 100% similar to lmo0788 |
| AARY_0483_x_at | 99% similar to lmo2243 |
| AARY_0487_s_at | 100% similar to lmo2214 |
| AARY_0498_s_at | 100% similar to lmo2819 |
| AARY_0516_s_at | 99% similar to lmo0788 |
| AARY_0533_s_at | 99% similar to lmo2178 |
| AARY_0549_s_at | 100% similar to lmo0199 |
| AARY_0596_s_at | 100% similar to lmo0421 |
| AARY_0602_s_at | 99% similar to lmo2119 |
| AARY_0629_x_at | 100% similar to lmo0263 |
| AARY_0636_s_at | 100% similar to lmo0773 |
| AARY_0667_s_at | 100% similar to lmo0534 |
| AARY_0676_s_at | 99% similar to lmo0501 |
| AARY_0679_s_at | 99% similar to lmo0425 |
| AARY_0681_at | 99% similar to lmo0426 |
| AARY_0681_x_at | 99% similar to lmo0426 |
| AARY_0684_s_at | 99% similar to lmo0401 |
| AARY_0686_s_at | 99% similar to lmo0402 |
| AARY_0689_s_at | 99% similar to lmo1939 |
| AARY_0699_s_at | 99% similar to lmo1275 |
| AARY_0700_s_at | 100% similar to lmo1274 |
| AARY_0704_at | 100% similar to lmo1501 |
| AARY_0720_x_at | 99% similar to lmo0595 |
| AARY_0726_s_at | 99% similar to lmo2787 |
| AARY_0727_s_at | 99% similar to lmo2787 |
| AARY_0729_s_at | 100% similar to lmo1742 |
| AARY_0746_s_at | 99% similar to lmo2115 |
| AARY_0749_s_at | 99% similar to lmo1804 |
| AARY_0750_s_at | 100% similar to lmo1804 |
| AARY_0766_s_at | 99% similar to lmo0183 |
| AARY_0770_s_at | 99% similar to lmo1899 |
| AARY_0778_s_at | 100% similar to lmo1061 |
| AARY_0779_s_at | 100% similar to lmo1062 |
| AARY_0780_s_at | 99% similar to lmo1062 |
| AARY_0798_s_at | 100% similar to lmo0361 |
| AARY_0798_x_at | 100% similar to lmo0361 |
| AARY_0808_at | 100% similar to lmo2110 |
| AARY_0809_s_at | 99% similar to lmo2108 |
| AARY_0815_s_at | 100% similar to lmo1627 |
| AARY_0816_s_at | 100% similar to lmo1628 |
| AARY_0828_s_at | 100% similar to lmo0829 |
| AARY_0854_x_at | 100% similar to lmo2766 |
| AARY_0862_s_at | 100% similar to lmo1645 |
| AARY_0881_s_at | 99% similar to lmo1690 |
| AARY_0922_s_at | 100% similar to lmo0897 |
| AARY_0933_s_at | 99% similar to lmo0659 |
| AARY_0942_s_at | 99% similar to lmo1681 |
| AARY_0946_s_at | NK |
| AARY_0953_x_at | 100% similar to lmo2401 |
| AARY_0966_s_at | 99% similar to lmo1871 |
| AARY_0967_s_at | 99% similar to lmo1475 |
| AARY_0987_s_at | 100% similar to lmo1397 |
| AARY_0993_s_at | 100% similar to lmo2175 |
| AARY_1012_s_at | 100% similar to lmo1513 |
| AARY_1024_s_at | 99% similar to lmo2039 |
| AARY_1029_s_at | 99% similar to lmo1311 |
| AARY_1032_s_at | 99% similar to lmo1835 |
| AARY_1033_s_at | 100% similar to lmo1836 |
| AARY_1076_s_at | 100% similar to lmo0933 |
| AARY_1094_s_at | 100% similar to lmo1458 |
| AARY_1103_s_at | 100% similar to lmo2179 |
| AARY_1104_s_at | 100% similar to lmo1224 |
| AARY_1109_s_at | 100% similar to lmo2157 |
| AARY_1117_s_at | 99% similar to lmo0627 |
| AARY_1118_s_at | 100% similar to lmo0627 |
| AARY_1122_s_at | 99% similar to lmo2099 |
| AARY_1124_s_at | 99% similar to lmo1357 |
| AARY_1134_s_at | 100% similar to lmo2547 |
| AARY_1150_s_at | 100% similar to lmo1853 |
| AARY_1160_s_at | 99% similar to lmo1527 |
| AARY_1177_x_at | 100% similar to lmo1431 |
| AARY_1180_s_at | 99% similar to lmo0964 |
| AARY_1201_s_at | 100% similar to lmo2573 |
| AARY_1203_s_at | 100% similar to lmo0288 |
| AARY_1208_x_at | NK |
| AARY_1222_s_at | 100% similar to lmo1360 |
| AARY_1230_s_at | 99% similar to lmo0543 |
| AARY_1306_s_at | 100% similar to lmo1378 |
| AARY_1323_s_at | 100% similar to lmo1680 |
| AARY_1324_s_at | 100% similar to lmo1679 |
| AARY_1338_s_at | 100% similar to lmo1325 |
| AARY_1348_s_at | 100% similar to lmo1378 |
| AARY_1355_s_at | 100% similar to lmo1633 |
| AARY_1368_s_at | 100% similar to lmo0559 |
| AARY_1370_s_at | 99% similar to lmo0618 |
| AARY_1374_s_at | 99% similar to lmo0841 |
| AARY_1382_s_at | 99% similar to lmo1624 |
| AARY_1392_s_at | 99% similar to lmo1525 |
| AARY_1410_s_at | 100% similar to lmo2268 |
| AARY_1418_s_at | 100% similar to lmo1286 |
| AARY_1429_s_at | 100% similar to lmo1871 |
| AARY_1460_s_at | 99% similar to lmo1614 |
| AARY_1467_s_at | 100% similar to lmo1441 |
| AARY_1470_s_at | 99% similar to lmo0524 |
| AARY_1476_s_at | 100% similar to lmo2525 |
| AARY_1493_s_at | 98% similar to lmo1224 |
| AARY_1498_s_at | 100% similar to lmo1574 |
| AARY_1502_at | NK |
| AARY_1528_s_at | 100% similar to lmo0873 |
| AARY_1529_s_at | 100% similar to lmo1300 |
| AARY_1544_s_at | 100% similar to lmo1513 |
| AARY_1549_s_at | 100% similar to lmo0739 |
| AARY_1550_s_at | 100% similar to lmo0679 |
| AARY_1553_s_at | 99% similar to lmo2091 |
| AARY_1553_x_at | 99% similar to lmo2091 |
| AARY_1558_s_at | NK |
| AARY_1563_s_at | 99% similar to lmo1405 |
| AARY_1569_s_at | 99% similar to lmo0203 |
| AARY_1570_s_at | 100% similar to lmo1660 |
| AARY_1572_s_at | 99% similar to lmo2092 |
| AARY_1578_s_at | 100% similar to lmo1574 |
| AARY_1584_s_at | 99% similar to lmo0182 |
| AARY_1587_s_at | 99% similar to lmo2576 |
| AARY_1587_x_at | 99% similar to lmo2576 |
| AARY_1593_at | NK |
| AARY_1593_x_at | NK |
| AARY_1607_x_at | 100% similar to lmo2815 |
| IGLMHCC_0081_x_at | Intergenic region |
| IGLMHCC_0284_s_at | Intergenic region |
| IGLMHCC_0287_at | Intergenic region |
| IGLMHCC_0288_s_at | Intergenic region |
| IGLMHCC_0369_at | Intergenic region |
| IGLMHCC_0369_x_at | Intergenic region |
| IGLMHCC_0584_at | Intergenic region |
| IGLMHCC_0689_at | Intergenic region |
| IGLMHCC_1114_s_at | Intergenic region |
| IGLMHCC_1205_s_at | Intergenic region |
| IGLMHCC_1243_x_at | Intergenic region |
| IGLMHCC_1334_at | Intergenic region |
| IGLMHCC_1596_x_at | Intergenic region |
| IGLMHCC_1614_at | Intergenic region |
| IGLMHCC_1703_x_at | Intergenic region |
| IGLMHCC_1833_x_at | Intergenic region |
| IGLMHCC_1856_at | Intergenic region |
| IGLMHCC_1866_x_at | Intergenic region |
| IGLMHCC_1878_at | Intergenic region |
| IGLMHCC_1882_at | Intergenic region |
| IGLMHCC_2020_at | Intergenic region |
| IGLMHCC_2144_x_at | Intergenic region |
| IGLMHCC_2216_s_at | Intergenic region |
| IGLMHCC_2312_at | Intergenic region |
| IGLMHCC_2371_x_at | Intergenic region |
| IGLMHCC_2470_s_at | Intergenic region |
| IGLMHCC_2523_s_at | Intergenic region |
| IGLMHCC_2683_x_at | Intergenic region |
| IGLMHCC_2787_at | Intergenic region |
| IGlmo0013_at | Intergenic region |
| IGlmo0020_at | Intergenic region |
| IGlmo0030_x_at | Intergenic region |
| IGlmo0032_at | Intergenic region |
| IGlmo0032_x_at | Intergenic region |
| IGlmo0044_x_at | Intergenic region |
| IGlmo0055_at | Intergenic region |
| IGlmo0056_at | Intergenic region |
| IGlmo0056_x_at | Intergenic region |
| IGlmo0084_at | Intergenic region |
| IGlmo0084_x_at | Intergenic region |
| IGlmo0085_at | Intergenic region |
| IGlmo0085_x_at | Intergenic region |
| IGlmo0086_at | Intergenic region |
| IGlmo0095_at | Intergenic region |
| IGlmo0096_at | Intergenic region |
| IGlmo0096_x_at | Intergenic region |
| IGlmo0100_at | Intergenic region |
| IGlmo0100_x_at | Intergenic region |
| IGlmo0110_x_at | Intergenic region |
| IGlmo0111_at | Intergenic region |
| IGlmo0117_at | Intergenic region |
| IGlmo0157_at | Intergenic region |
| IGlmo0157_x_at | Intergenic region |
| IGlmo0159_at | Intergenic region |
| IGlmo0160_at | Intergenic region |
| IGlmo0171_at | Intergenic region |
| IGlmo0174_at | Intergenic region |
| IGlmo0175_s_at | Intergenic region |
| IGlmo0178_x_at | Intergenic region |
| IGlmo0186_at | Intergenic region |
| IGlmo0196_at | Intergenic region |
| IGlmo0198_at | Intergenic region |
| IGlmo0198_x_at | Intergenic region |
| IGlmo0203_at | Intergenic region |
| IGlmo0207_x_at | Intergenic region |
| IGlmo0218_at | Intergenic region |
| IGlmo0252_at | Intergenic region |
| IGlmo0252_x_at | Intergenic region |
| IGlmo0256_s_at | Intergenic region |
| IGlmo0264_at | Intergenic region |
| IGlmo0264_x_at | Intergenic region |
| IGlmo0265_at | Intergenic region |
| IGlmo0265_x_at | Intergenic region |
| IGlmo0275_at | Intergenic region |
| IGlmo0275_x_at | Intergenic region |
| IGlmo0282_x_at | Intergenic region |
| IGlmo0333_at | Intergenic region |
| IGlmo0339_at | Intergenic region |
| IGlmo0341_at | Intergenic region |
| IGlmo0342_at | Intergenic region |
| IGlmo0353_s_at | Intergenic region |
| IGlmo0354_at | Intergenic region |
| IGlmo0356_at | Intergenic region |
| IGlmo0357_at | Intergenic region |
| IGlmo0357_x_at | Intergenic region |
| IGlmo0361_at | Intergenic region |
| IGlmo0361_x_at | Intergenic region |
| IGlmo0371_x_at | Intergenic region |
| IGlmo0382_x_at | Intergenic region |
| IGlmo0390_at | Intergenic region |
| IGlmo0397_at | Intergenic region |
| IGlmo0397_x_at | Intergenic region |
| IGlmo0403_x_at | Intergenic region |
| IGlmo0413_at | Intergenic region |
| IGlmo0413_x_at | Intergenic region |
| IGlmo0416_x_at | Intergenic region |
| IGlmo0420_at | Intergenic region |
| IGlmo0420_x_at | Intergenic region |
| IGlmo0421_at | Intergenic region |
| IGlmo0459_at | Intergenic region |
| IGlmo0460_at | Intergenic region |
| IGlmo0478_at | Intergenic region |
| IGlmo0479_at | Intergenic region |
| IGlmo0485_x_at | Intergenic region |
| IGlmo0511_at | Intergenic region |
| IGlmo0525_at | Intergenic region |
| IGlmo0526_at | Intergenic region |
| IGlmo0527_at | Intergenic region |
| IGlmo0527_x_at | Intergenic region |
| IGlmo0536_at | Intergenic region |
| IGlmo0541_x_at | Intergenic region |
| IGlmo0548_at | Intergenic region |
| IGlmo0550_x_at | Intergenic region |
| IGlmo0554_at | Intergenic region |
| IGlmo0554_x_at | Intergenic region |
| IGlmo0561_at | Intergenic region |
| IGlmo0576_at | Intergenic region |
| IGlmo0576_x_at | Intergenic region |
| IGlmo0577_at | Intergenic region |
| IGlmo0585_x_at | Intergenic region |
| IGlmo0588_at | Intergenic region |
| IGlmo0588_x_at | Intergenic region |
| IGlmo0589_at | Intergenic region |
| IGlmo0594_x_at | Intergenic region |
| IGlmo0599_at | Intergenic region |
| IGlmo0610_at | Intergenic region |
| IGlmo0611_at | Intergenic region |
| IGlmo0611_x_at | Intergenic region |
| IGlmo0614_at | Intergenic region |
| IGlmo0617_x_at | Intergenic region |
| IGlmo0624_at | Intergenic region |
| IGlmo0626_at | Intergenic region |
| IGlmo0626_x_at | Intergenic region |
| IGlmo0628_x_at | Intergenic region |
| IGlmo0629_x_at | Intergenic region |
| IGlmo0635_at | Intergenic region |
| IGlmo0635_x_at | Intergenic region |
| IGlmo0641_at | Intergenic region |
| IGlmo0654_x_at | Intergenic region |
| IGlmo0656_at | Intergenic region |
| IGlmo0660_at | Intergenic region |
| IGlmo0661_at | Intergenic region |
| IGlmo0671_at | Intergenic region |
| IGlmo0671_x_at | Intergenic region |
| IGlmo0672_at | Intergenic region |
| IGlmo0691_at | Intergenic region |
| IGlmo0691_x_at | Intergenic region |
| IGlmo0719_at | Intergenic region |
| IGlmo0719_x_at | Intergenic region |
| IGlmo0723_at | Intergenic region |
| IGlmo0723_x_at | Intergenic region |
| IGlmo0732_at | Intergenic region |
| IGlmo0733_x_at | Intergenic region |
| IGlmo0734_at | Intergenic region |
| IGlmo0735_at | Intergenic region |
| IGlmo0735_x_at | Intergenic region |
| IGlmo0741_at | Intergenic region |
| IGlmo0747_at | Intergenic region |
| IGlmo0773_at | Intergenic region |
| IGlmo0778_at | Intergenic region |
| IGlmo0781_at | Intergenic region |
| IGlmo0785_at | Intergenic region |
| IGlmo0788_at | Intergenic region |
| IGlmo0788_x_at | Intergenic region |
| IGlmo0792_x_at | Intergenic region |
| IGlmo0826_at | Intergenic region |
| IGlmo0827_x_at | Intergenic region |
| IGlmo0830_at | Intergenic region |
| IGlmo0831_x_at | Intergenic region |
| IGlmo0832_at | Intergenic region |
| IGlmo0833_at | Intergenic region |
| IGlmo0833_x_at | Intergenic region |
| IGlmo0834_at | Intergenic region |
| IGlmo0840_at | Intergenic region |
| IGlmo0840_x_at | Intergenic region |
| IGlmo0841_at | Intergenic region |
| IGlmo0841_x_at | Intergenic region |
| IGlmo0842_at | Intergenic region |
| IGlmo0843_at | Intergenic region |
| IGlmo0866_at | Intergenic region |
| IGlmo0867_at | Intergenic region |
| IGlmo0913_at | Intergenic region |
| IGlmo0919_at | Intergenic region |
| IGlmo0943_at | Intergenic region |
| IGlmo0944_at | Intergenic region |
| IGlmo0956_at | Intergenic region |
| IGlmo0956_x_at | Intergenic region |
| IGlmo0975_at | Intergenic region |
| IGlmo0975_x_at | Intergenic region |
| IGlmo0978_at | Intergenic region |
| IGlmo0993_x_at | Intergenic region |
| IGlmo1007_at | Intergenic region |
| IGlmo1009_at | Intergenic region |
| IGlmo1009_x_at | Intergenic region |
| IGlmo1014_at | Intergenic region |
| IGlmo1014_x_at | Intergenic region |
| IGlmo1018_at | Intergenic region |
| IGlmo1018_x_at | Intergenic region |
| IGlmo1026_x_at | Intergenic region |
| IGlmo1029_at | Intergenic region |
| IGlmo1031_at | Intergenic region |
| IGlmo1031_x_at | Intergenic region |
| IGlmo1050_at | Intergenic region |
| IGlmo1050_x_at | Intergenic region |
| IGlmo1052_at | Intergenic region |
| IGlmo1056_at | Intergenic region |
| IGlmo1056_x_at | Intergenic region |
| IGlmo1060_at | Intergenic region |
| IGlmo1062_at | Intergenic region |
| IGlmo1073_at | Intergenic region |
| IGlmo1136_at | Intergenic region |
| IGlmo1137_at | Intergenic region |
| IGlmo1138_s_at | Intergenic region |
| IGlmo1150_at | Intergenic region |
| IGlmo1215_at | Intergenic region |
| IGlmo1216_at | Intergenic region |
| IGlmo1216_x_at | Intergenic region |
| IGlmo1227_at | Intergenic region |
| IGlmo1227_x_at | Intergenic region |
| IGlmo1235_at | Intergenic region |
| IGlmo1235_x_at | Intergenic region |
| IGlmo1236_at | Intergenic region |
| IGlmo1236_x_at | Intergenic region |
| IGlmo1241_at | Intergenic region |
| IGlmo1242_at | Intergenic region |
| IGlmo1253_at | Intergenic region |
| IGlmo1263_at | Intergenic region |
| IGlmo1264_at | Intergenic region |
| IGlmo1266_at | Intergenic region |
| IGlmo1266_x_at | Intergenic region |
| IGlmo1274_at | Intergenic region |
| IGlmo1276_at | Intergenic region |
| IGlmo1285_at | Intergenic region |
| IGlmo1294_at | Intergenic region |
| IGlmo1296_x_at | Intergenic region |
| IGlmo1300_at | Intergenic region |
| IGlmo1300_x_at | Intergenic region |
| IGlmo1301_at | Intergenic region |
| IGlmo1304_at | Intergenic region |
| IGlmo1304_x_at | Intergenic region |
| IGlmo1313_at | Intergenic region |
| IGlmo1375_at | Intergenic region |
| IGlmo1385_at | Intergenic region |
| IGlmo1385_x_at | Intergenic region |
| IGlmo1389_at | Intergenic region |
| IGlmo1398_at | Intergenic region |
| IGlmo1406_at | Intergenic region |
| IGlmo1409_at | Intergenic region |
| IGlmo1410_at | Intergenic region |
| IGlmo1412_at | Intergenic region |
| IGlmo1414_at | Intergenic region |
| IGlmo1425_x_at | Intergenic region |
| IGlmo1432_x_at | Intergenic region |
| IGlmo1480_at | Intergenic region |
| IGlmo1509_x_at | Intergenic region |
| IGlmo1511_x_at | Intergenic region |
| IGlmo1515_at | Intergenic region |
| IGlmo1516_at | Intergenic region |
| IGlmo1516_x_at | Intergenic region |
| IGlmo1522_x_at | Intergenic region |
| IGlmo1527_at | Intergenic region |
| IGlmo1527_x_at | Intergenic region |
| IGlmo1534_at | Intergenic region |
| IGlmo1534_x_at | Intergenic region |
| IGlmo1536_at | Intergenic region |
| IGlmo1536_x_at | Intergenic region |
| IGlmo1549_at | Intergenic region |
| IGlmo1566_at | Intergenic region |
| IGlmo1570_at | Intergenic region |
| IGlmo1571_at | Intergenic region |
| IGlmo1580_x_at | Intergenic region |
| IGlmo1581_x_at | Intergenic region |
| IGlmo1604_at | Intergenic region |
| IGlmo1605_at | Intergenic region |
| IGlmo1605_x_at | Intergenic region |
| IGlmo1617_at | Intergenic region |
| IGlmo1619_at | Intergenic region |
| IGlmo1634_at | Intergenic region |
| IGlmo1641_at | Intergenic region |
| IGlmo1641_x_at | Intergenic region |
| IGlmo1653_at | Intergenic region |
| IGlmo1655_at | Intergenic region |
| IGlmo1655_x_at | Intergenic region |
| IGlmo1665_at | Intergenic region |
| IGlmo1726_x_at | Intergenic region |
| IGlmo1751_at | Intergenic region |
| IGlmo1754_at | Intergenic region |
| IGlmo1754_x_at | Intergenic region |
| IGlmo1764_at | Intergenic region |
| IGlmo1782_at | Intergenic region |
| IGlmo1782_x_at | Intergenic region |
| IGlmo1786_at | Intergenic region |
| IGlmo1801_at | Intergenic region |
| IGlmo1801_x_at | Intergenic region |
| IGlmo1814_at | Intergenic region |
| IGlmo1816_at | Intergenic region |
| IGlmo1871_at | Intergenic region |
| IGlmo1877_at | Intergenic region |
| IGlmo1877_x_at | Intergenic region |
| IGlmo1878_at | Intergenic region |
| IGlmo1879_at | Intergenic region |
| IGlmo1909_x_at | Intergenic region |
| IGlmo1911_at | Intergenic region |
| IGlmo1919_at | Intergenic region |
| IGlmo1919_x_at | Intergenic region |
| IGlmo1920_x_at | Intergenic region |
| IGlmo1921_at | Intergenic region |
| IGlmo1921_x_at | Intergenic region |
| IGlmo1929_at | Intergenic region |
| IGlmo1929_x_at | Intergenic region |
| IGlmo1936_at | Intergenic region |
| IGlmo1936_x_at | Intergenic region |
| IGlmo1939_x_at | Intergenic region |
| IGlmo1946_at | Intergenic region |
| IGlmo1946_x_at | Intergenic region |
| IGlmo1965_at | Intergenic region |
| IGlmo1965_x_at | Intergenic region |
| IGlmo1966_at | Intergenic region |
| IGlmo1975_at | Intergenic region |
| IGlmo1976_x_at | Intergenic region |
| IGlmo1983_at | Intergenic region |
| IGlmo1987_at | Intergenic region |
| IGlmo2034_at | Intergenic region |
| IGlmo2059_at | Intergenic region |
| IGlmo2059_x_at | Intergenic region |
| IGlmo2064_at | Intergenic region |
| IGlmo2064_x_at | Intergenic region |
| IGlmo2067_s_at | Intergenic region |
| IGlmo2068_at | Intergenic region |
| IGlmo2068_x_at | Intergenic region |
| IGlmo2075_at | Intergenic region |
| IGlmo2086_s_at | Intergenic region |
| IGlmo2100_at | Intergenic region |
| IGlmo2100_x_at | Intergenic region |
| IGlmo2106_at | Intergenic region |
| IGlmo2107_x_at | Intergenic region |
| IGlmo2109_at | Intergenic region |
| IGlmo2110_at | Intergenic region |
| IGlmo2121_at | Intergenic region |
| IGlmo2121_x_at | Intergenic region |
| IGlmo2129_x_at | Intergenic region |
| IGlmo2131_s_at | Intergenic region |
| IGlmo2132_at | Intergenic region |
| IGlmo2139_at | Intergenic region |
| IGlmo2145_at | Intergenic region |
| IGlmo2145_x_at | Intergenic region |
| IGlmo2155_at | Intergenic region |
| IGlmo2158_at | Intergenic region |
| IGlmo2166_at | Intergenic region |
| IGlmo2166_x_at | Intergenic region |
| IGlmo2170_at | Intergenic region |
| IGlmo2173_at | Intergenic region |
| IGlmo2174_at | Intergenic region |
| IGlmo2175_at | Intergenic region |
| IGlmo2175_x_at | Intergenic region |
| IGlmo2176_at | Intergenic region |
| IGlmo2176_x_at | Intergenic region |
| IGlmo2177_at | Intergenic region |
| IGlmo2177_x_at | Intergenic region |
| IGlmo2178_at | Intergenic region |
| IGlmo2180_x_at | Intergenic region |
| IGlmo2187_at | Intergenic region |
| IGlmo2187_x_at | Intergenic region |
| IGlmo2188_at | Intergenic region |
| IGlmo2191_at | Intergenic region |
| IGlmo2199_at | Intergenic region |
| IGlmo2202_x_at | Intergenic region |
| IGlmo2204_at | Intergenic region |
| IGlmo2205_x_at | Intergenic region |
| IGlmo2229_x_at | Intergenic region |
| IGlmo2233_x_at | Intergenic region |
| IGlmo2234_at | Intergenic region |
| IGlmo2239_x_at | Intergenic region |
| IGlmo2254_at | Intergenic region |
| IGlmo2256_at | Intergenic region |
| IGlmo2259_at | Intergenic region |
| IGlmo2259_x_at | Intergenic region |
| IGlmo2270_at | Intergenic region |
| IGlmo2334_at | Intergenic region |
| IGlmo2338_at | Intergenic region |
| IGlmo2338_x_at | Intergenic region |
| IGlmo2340_at | Intergenic region |
| IGlmo2354_at | Intergenic region |
| IGlmo2362_at | Intergenic region |
| IGlmo2366_at | Intergenic region |
| IGlmo2370_at | Intergenic region |
| IGlmo2376_s_at | Intergenic region |
| IGlmo2385_s_at | Intergenic region |
| IGlmo2388_at | Intergenic region |
| IGlmo2394_x_at | Intergenic region |
| IGlmo2447_at | Intergenic region |
| IGlmo2447_x_at | Intergenic region |
| IGlmo2451_x_at | Intergenic region |
| IGlmo2452_at | Intergenic region |
| IGlmo2452_x_at | Intergenic region |
| IGlmo2466_at | Intergenic region |
| IGlmo2478_at | Intergenic region |
| IGlmo2478_x_at | Intergenic region |
| IGlmo2484_at | Intergenic region |
| IGlmo2484_x_at | Intergenic region |
| IGlmo2488_at | Intergenic region |
| IGlmo2490_at | Intergenic region |
| IGlmo2490_x_at | Intergenic region |
| IGlmo2500_at | Intergenic region |
| IGlmo2504_x_at | Intergenic region |
| IGlmo2519_x_at | Intergenic region |
| IGlmo2522_at | Intergenic region |
| IGlmo2523_at | Intergenic region |
| IGlmo2524_at | Intergenic region |
| IGlmo2524_x_at | Intergenic region |
| IGlmo2537_at | Intergenic region |
| IGlmo2538_at | Intergenic region |
| IGlmo2538_x_at | Intergenic region |
| IGlmo2556_at | Intergenic region |
| IGlmo2559_at | Intergenic region |
| IGlmo2560_at | Intergenic region |
| IGlmo2569_at | Intergenic region |
| IGlmo2576_at | Intergenic region |
| IGlmo2576_x_at | Intergenic region |
| IGlmo2577_at | Intergenic region |
| IGlmo2580_at | Intergenic region |
| IGlmo2603_at | Intergenic region |
| IGlmo2603_x_at | Intergenic region |
| IGlmo2604_at | Intergenic region |
| IGlmo2605_at | Intergenic region |
| IGlmo2605_x_at | Intergenic region |
| IGlmo2634_at | Intergenic region |
| IGlmo2636_at | Intergenic region |
| IGlmo2638_at | Intergenic region |
| IGlmo2687_at | Intergenic region |
| IGlmo2700_at | Intergenic region |
| IGlmo2703_at | Intergenic region |
| IGlmo2705_s_at | Intergenic region |
| IGlmo2711_at | Intergenic region |
| IGlmo2715_at | Intergenic region |
| IGlmo2715_x_at | Intergenic region |
| IGlmo2720_at | Intergenic region |
| IGlmo2744_at | Intergenic region |
| IGlmo2745_at | Intergenic region |
| IGlmo2745_x_at | Intergenic region |
| IGlmo2758_at | Intergenic region |
| IGlmo2766_x_at | Intergenic region |
| IGlmo2771_x_at | Intergenic region |
| IGlmo2786_at | Intergenic region |
| IGlmo2786_x_at | Intergenic region |
| IGlmo2788_at | Intergenic region |
| IGlmo2789_at | Intergenic region |
| IGlmo2807_at | Intergenic region |
| IGlmo2820_at | Intergenic region |
| IGlmo2829_at | Intergenic region |
| IGlmo2829_x_at | Intergenic region |
| IGlmo2843_x_at | Intergenic region |
| IGlmo2852_at | Intergenic region |
| IGlmo2852_x_at | Intergenic region |
| IGlmo2875_x_at | Intergenic region |
| IGlmo2879_at | Intergenic region |
| IGlmo2909_at | Intergenic region |
| IGlmo2910_at | Intergenic region |
| IGlmo2915_at | Intergenic region |
| IGlmo2915_x_at | Intergenic region |
| IGlmo2934_at | Intergenic region |
| LMBG_00120_s_at | conserved hypothetical protein |
| LMBG_02642_x_at | carbamoylphosphate synthase/Pfam=PF02787.11 |
| LMHCC_0259_s_at | L-cystine transport system permease protein TcyM/GI=217332825 |
| LMHCC_0277_s_at | fumarylacetoacetate hydrolase family protein/GI=217332843 |
| LMHCC_0279_s_at | YitT/GI=217332845 |
| LMHCC_0280_s_at | HAD-superfamily hydrolase, subfamily IIB/GI=217332846 |
| LMHCC_0281_s_at | conserved hypothetical protein/GI=217332847 |
| LMHCC_0284_s_at | acetyltransferase, gnat family/GI=217332850 |
| LMHCC_0385_s_at | sugar phosphate isomeraseepimerase/GI=217332950 |
| LMHCC_0458_s_at | argH argininosuccinate lyase/GI=217333021 |
| LMHCC_0500_s_at | mosc domain protein/GI=217333063 |
| LMHCC_0539_s_at | 3H domain protein/GI=217333102 |
| LMHCC_0580_x_at | conserved hypothetical protein/GI=217333143 |
| LMHCC_0619_s_at | GTP-binding protein EngA/GI=217333181 |
| LMHCC_0686_s_at | DedA family protein/GI=217333247 |
| LMHCC_0715_at | stas domain protein/GI=217333276 |
| LMHCC_1084_s_at | iojap-related protein/GI=217333638 |
| LMHCC_1110_s_at | recO DNA repair protein RecO/GI=217333664 |
| LMHCC_1113_s_at | conserved hypothetical protein/GI=217333667 |
| LMHCC_1330_s_at | conserved hypothetical protein/GI=217333881 |
| LMHCC_1442_s_at | ATP:cob(I)alamin adenosyltransferase/GI=217333993 |
| LMHCC_1632_s_at | mate efflux family protein/GI=217334180 |
| LMHCC_1764_s_at | conserved hypothetical protein/GI=217334311 |
| LMHCC_1770_s_at | LacI family transcriptional regulator/GI=217334317 |
| LMHCC_1772_s_at | UDP-N-acetylmuramoyl-tripeptide--D-alanyl-D-alanine ligase (UDP-MurNAc-pentapeptide synthetase) (D-alanyl-D-alanine-adding enzyme)/GI=217334319 |
| LMHCC_1776_s_at | transcriptional regulator, TetR family/GI=217334323 |
| LMHCC_1815_x_at | HD domain protein/GI=217334362 |
| LMHCC_1836_s_at | transcriptional regulator, MarR family/GI=217334383 |
| LMHCC_1852_s_at | fructose permease iic component (pts system fructose-specific eiiccomponent) (eiic-fru) (p28)/GI=217334399 |
| LMHCC_1855_s_at | conserved hypothetical protein/GI=217334402 |
| LMHCC_1858_s_at | ROK family protein/GI=217334405 |
| LMHCC_1873_s_at | lipolytic protein G-D-S-L family/GI=217334420 |
| LMHCC_1878_s_at | conserved hypothetical protein/GI=217334425 |
| LMHCC_1879_s_at | conserved domain protein/GI=217334426 |
| LMHCC_1880_s_at | conserved hypothetical protein/GI=217334427 |
| LMHCC_1881_s_at | conserved hypothetical protein/GI=217334428 |
| LMHCC_1926_s_at | flagellar motor switch protein/GI=217334473 |
| LMHCC_1989_s_at | membrane sulfatase family protein/GI=217334534 |
| LMHCC_2020_s_at | rhodanese domain protein/GI=217334565 |
| LMHCC_2055_s_at | secreted MUCin-binding domain protein/GI=217334600 |
| LMHCC_2164_x_at | transposase OrfA, IS3 family, putative/GI=217334708 |
| LMHCC_2300_s_at | conserved hypothetical protein/GI=217334844 |
| LMHCC_2304_s_at | cell wall surface anchor family protein/GI=217334848 |
| LMHCC_2310_s_at | lipoprotein, putative/GI=217334853 |
| LMHCC_2333_s_at | PRDPTS system IIA 2 domain regulatory protein/GI=217334876 |
| LMHCC_2367_s_at | glyoxalase family protein/GI=217334910 |
| LMHCC_2476_s_at | signal peptidase II/GI=217335017 |
| LMHCC_2521_s_at | ROK family protein/GI=217335062 |
| LMHCC_2641_s_at | PTS system, mannosefructosesorbose family, IIC component/GI=217335182 |
| LMHCC_2664_s_at | conserved hypothetical protein/GI=217335205 |
| LMHCC_2688_s_at | kojibiose phosphorylase/GI=217335229 |
| LMHCC_2721_s_at | conserved hypothetical protein/GI=217335262 |
| LMHCC_2722_x_at | conserved hypothetical protein/GI=217335263 |
| LMHCC_2728_s_at | PTS system, IIA component/GI=217335269 |
| LMHCC_2732_x_at | lipoprotein, putative/GI=217335273 |
| LMHCC_2784_s_at | general stress protein 26 (GSP26)/GI=217335324 |
| LMHCC_2786_s_at | TfoX domain protein/GI=217335326 |
| LMHCC_2872_s_at | rpiB ribose 5-phosphate isomerase B/GI=217335409 |
| LMHG_00222_s_at | leucine rich repeat domaincontaining protein |
| LMHG_00343_s_at | acetyltransferase/Pfam=PF00132.16 |
| LMHG_00509_s_at | conserved hypothetical protein |
| LMHG_00924_s_at | ABC transporter/Pfam=PF00664.15 |
| LMHG_01121_s_at | conserved hypothetical protein/Pfam=PF05991.3 |
| LMHG_01943_x_at | conserved hypothetical protein/Pfam=PF02302.9 |
| LMHG_01948_x_at | conserved hypothetical protein |
| LMHG_02083_x_at | conserved hypothetical protein |
| LMHG_03192_s_at | ATPdependent nuclease |
| LMHG_03192_x_at | ATPdependent nuclease |
| LMHG_03208_s_at | PTS system protein/Pfam=PF00358.12 |
| LMIG_00209_x_at | conserved hypothetical protein/Pfam=PF01381.14 |
| LMIG_00215_x_at | hydrolase/Pfam=PF00702.18 |
| LMIG_00445_x_at | conserved hypothetical protein |
| LMIG_00469_s_at | primosome assembly protein PriA/Pfam=PF04851.7 |
| LMIG_00479_s_at | pyrAB/Pfam=PF02787.11 |
| LMIG_00489_s_at | conserved hypothetical protein |
| LMIG_00500_s_at | purine nucleoside phosphorylase deoDtype/Pfam=PF01048.12 |
| LMIG_00559_s_at | NADPdependent malic enzyme/Pfam=PF03949.7 |
| LMIG_00640_s_at | deoxyribosephosphate aldolase/Pfam=PF01791.1 |
| LMIG_00681_s_at | Nacetylglucosaminyl transferase/Pfam=PF04101.8 |
| LMIG_00704_s_at | protoheme IX farnesyltransferase/Pfam=PF01040.10 |
| LMIG_00763_s_at | conserved hypothetical protein/Pfam=PF06778.4 |
| LMIG_00772_s_at | conserved hypothetical protein |
| LMIG_00901_s_at | hisGluGlnArgopine ABC transporter permease/Pfam=PF00528.14 |
| LMIG_00931_s_at | monooxygenase |
| LMIG_00977_x_at | nitrogen regulatory protein PII/Pfam=PF00543.14 |
| LMIG_01067_x_at | predicted protein |
| LMIG_01087_s_at | polysaccharide biosynthesis family protein/Pfam=PF01943.9 |
| LMIG_01307_s_at | precorrin6X reductase/Pfam=PF02571.6 |
| LMIG_01566_x_at | transcriptional regulator/Pfam=PF01022.12 |
| LMIG_01617_s_at | peptide chain release factor 1/Pfam=PF03462.10 |
| LMIG_01632_s_at | autolysin/Pfam=PF01510.17 |
| LMIG_01634_s_at | transmembrane protein |
| LMIG_01684_s_at | conserved hypothetical protein |
| LMIG_01816_x_at | hydrolase/Pfam=PF08282.4 |
| LMIG_01829_x_at | methionine import ATPbinding protein metN/Pfam=PF09383.2 |
| LMIG_01895_s_at | transcriptional regulator/Pfam=PF01047.14 |
| LMIG_01936_s_at | lysM domaincontaining protein/Pfam=PF05737.4 |
| LMIG_01981_s_at | ABC transporter/Pfam=PF00005.19 |
| LMIG_02027_s_at | peptidoglycan binding protein/Pfam=PF07523.4 |
| LMIG_02097_s_at | conserved hypothetical protein |
| LMIG_02106_x_at | spermidineputrescine ABC transporter/Pfam=PF01547.17 |
| LMIG_02121_s_at | NaPi-cotransporter family protein/Pfam=PF02690.7 |
| LMIG_02130_s_at | ABC transporter/Pfam=PF00664.15 |
| LMIG_02157_s_at | conserved hypothetical protein |
| LMIG_02254_s_at | molybdopterin biosynthesis protein MoeA/Pfam=PF03454.7 |
| LMIG_02258_s_at | molybdenum cofactor biosynthesis protein C/Pfam=PF01967.13 |
| LMIG_02334_s_at | ABC transporter/Pfam=PF00005.19 |
| LMIG_02453_x_at | transcriptional regulator/Pfam=PF00376.15 |
| LMIG_02549_s_at | major facilitator family transporter/Pfam=PF07690.8 |
| LMIG_02557_s_at | helicase domaincontaining protein/Pfam=PF00271.23 |
| LMIG_02577_s_at | predicted protein |
| LMIG_02625_s_at | flagellar hookassociated protein FlgL/Pfam=PF00669.12 |
| LMIG_02666_s_at | conserved hypothetical protein/Pfam=PF00892.12 |
| LMIG_02705_x_at | conserved hypothetical protein |
| LMIG_02781_s_at | monovalent cationH+ antiporter subunit A/Pfam=PF00662.12 |
| LMIG_02843_x_at | conserved hypothetical protein/Pfam=PF01527.12 |
| LMIG_02849_x_at | conserved hypothetical protein |
| LMIG_02857_s_at | predicted protein |
| LMIG_02863_s_at | predicted protein |
| LMIG_02888_s_at | predicted protein |
| LMIG_02892_s_at | trpD/Pfam=PF00591.13 |
| LMIG_02893_s_at | predicted protein |
| LMIG_02910_s_at | predicted protein |
| LMIG_02918_at | predicted protein |
| LMIG_02918_x_at | predicted protein |
| LMIG_02929_s_at | predicted protein |
| LMIG_02931_s_at | conserved hypothetical protein |
| LMIG_02931_x_at | conserved hypothetical protein |
| LMIG_02933_s_at | conserved hypothetical protein |
| LMIG_02945_s_at | predicted protein |
| LMIG_02947_s_at | predicted protein |
| LMIG_02949_s_at | predicted protein/Pfam=PF05738.5 |
| LMKG_00432_s_at | conserved hypothetical protein |
| LMKG_00455_s_at | predicted protein |
| LMKG_00597_x_at | conserved hypothetical protein |
| LMKG_00886_s_at | conserved hypothetical protein |
| LMKG_00917_s_at | predicted protein |
| LMKG_00923_s_at | predicted protein |
| LMKG_00949_s_at | ribonuclease HII |
| LMKG_00950_s_at | ribonuclease HII |
| LMKG_01071_x_at | predicted protein |
| LMKG_01096_s_at | predicted protein |
| LMKG_01145_s_at | predicted protein |
| LMKG_01228_s_at | predicted protein |
| LMKG_01270_x_at | conserved hypothetical protein |
| LMKG_01576_at | predicted protein |
| LMKG_01581_x_at | predicted protein |
| LMKG_01871_x_at | conserved hypothetical protein |
| LMKG_01986_s_at | conserved hypothetical protein/Pfam=PF02518.18 |
| LMKG_02134_x_at | predicted protein |
| LMKG_02149_s_at | autolysin/Pfam=PF01832.12 |
| LMKG_02316_s_at | predicted protein |
| LMKG_02358_at | predicted protein |
| LMKG_02513_s_at | predicted protein |
| LMKG_02694_s_at | predicted protein |
| LMKG_02905_s_at | cobalt ABC transporter |
| LMKG_03009_s_at | conserved hypothetical protein/Pfam=PF02126.10 |
| LMKG_03088_x_at | conserved hypothetical protein |
| LMKG_03122_s_at | conserved hypothetical protein |
| LMLG_00020_x_at | conserved hypothetical protein |
| LMLG_00086_x_at | pentapeptide repeats domaincontaining protein/Pfam=PF00805.14 |
| LMLG_00208_s_at | Disomer specific 2hydroxyacid dehydrogenase family protein/Pfam=PF02826.11 |
| LMLG_00271_s_at | DalanylDalanine carboxypeptidase/Pfam=PF00768.12 |
| LMLG_00418_s_at | conserved hypothetical protein/Pfam=PF09261.3 |
| LMLG_00440_s_at | conserved hypothetical protein/Pfam=PF00585.10 |
| LMLG_00473_s_at | ferrichrome ABC transporter/Pfam=PF01032.10 |
| LMLG_00641_x_at | conserved hypothetical protein |
| LMLG_00696_s_at | conserved hypothetical protein |
| LMLG_00833_s_at | conserved hypothetical protein |
| LMLG_00837_s_at | iron compound ABC transporter |
| LMLG_00838_s_at | iron compound ABC transporter/Pfam=PF01032.10 |
| LMLG_00840_s_at | conserved hypothetical protein/Pfam=PF05031.4 |
| LMLG_00868_s_at | uroporphyrinogen decarboxylase/Pfam=PF01208.9 |
| LMLG_00914_s_at | aspartate aminotransferase |
| LMLG_00964_s_at | transcriptional regulator/Pfam=PF08280.3 |
| LMLG_01074_x_at | conserved hypothetical protein/Pfam=PF07883.3 |
| LMLG_01173_s_at | ABC transporter/Pfam=PF00664.15 |
| LMLG_01285_s_at | alcohol dehydrogenase |
| LMLG_01340_s_at | ABC transporter/Pfam=PF00005.19 |
| LMLG_01439_at | conserved hypothetical protein/Pfam=PF01594.8 |
| LMLG_01450_s_at | conserved hypothetical protein/Pfam=PF00953.13 |
| LMLG_01460_x_at | predicted protein |
| LMLG_01581_s_at | conserved hypothetical protein |
| LMLG_01624_s_at | amino acid permease family protein/Pfam=PF00324.13 |
| LMLG_01773_x_at | glutamyltRNA reductase/Pfam=PF05201.7 |
| LMLG_01841_x_at | conserved hypothetical protein/Pfam=PF01022.12 |
| LMLG_01845_s_at | phosphate ABC transporter |
| LMLG_01850_s_at | DNAbinding response regulator PhoP/Pfam=PF00486.20 |
| LMLG_01851_s_at | DNAbinding response regulator PhoP/Pfam=PF00072.16 |
| LMLG_01895_s_at | uridylate kinase/Pfam=PF00696.20 |
| LMLG_01914_s_at | polyribonucleotide nucleotidyltransferase/Pfam=PF00575.15 |
| LMLG_01956_s_at | 2oxoisovalerate dehydrogenase E3/Pfam=PF07992.6 |
| LMLG_01960_s_at | 2oxoisovalerate dehydrogenase E1 |
| LMLG_02010_s_at | predicted protein |
| LMLG_02015_s_at | conserved hypothetical protein/Pfam=PF00391.15 |
| LMLG_02156_s_at | conserved hypothetical protein |
| LMLG_02165_x_at | conserved hypothetical protein/Pfam=PF07843.3 |
| LMLG_02174_x_at | conserved hypothetical protein/Pfam=PF00746.13 |
| LMLG_02181_s_at | major facilitator family transporter/Pfam=PF07690.8 |
| LMLG_02235_s_at | conserved hypothetical protein |
| LMLG_02269_s_at | conserved hypothetical protein |
| LMLG_02270_s_at | conserved hypothetical protein |
| LMLG_02292_x_at | formate acetyltransferase |
| LMLG_02370_s_at | transcriptionrepair coupling factor/Pfam=PF04851.7 |
| LMLG_02411_s_at | cell wall surface anchor family protein/Pfam=PF06458.4 |
| LMLG_02443_s_at | ribulosephosphate 3epimerase/Pfam=PF01816.9 |
| LMLG_02461_s_at | conserved hypothetical protein/Pfam=PF03466.12 |
| LMLG_02600_x_at | conserved hypothetical protein/Pfam=PF00376.15 |
| LMLG_02771_s_at | conserved hypothetical protein/Pfam=PF08242.4 |
| LMLG_02807_s_at | predicted protein/Pfam=PF00746.13 |
| LMLG_02864_s_at | conserved hypothetical protein/Pfam=PF01757.14 |
| LMLG_02907_s_at | tyrA protein |
| LMMG_01283_at | acetyltransferase/Pfam=PF00583.16 |
| LMMG_02986_at | predicted protein |
| LMMG_02986_x_at | predicted protein |
| lmo0003_s_at | GI=16409362 |
| lmo0007_s_at | gyrA DNA gyrase subunit A/GI=16409366 |
| lmo0008_s_at | GI=16409367 |
| lmo0010_s_at | GI=16409369 |
| lmo0011_s_at | GI=16409370 |
| lmo0012_s_at | GI=16409371 |
| lmo0016_s_at | qoxD GI=16409375 |
| lmo0017_s_at | GI=16409376 |
| lmo0017_x_at | GI=16409376 |
| lmo0020_s_at | GI=16409379 |
| lmo0021_s_at | GI=16409380 |
| lmo0023_s_at | GI=16409382 |
| lmo0024_s_at | GI=16409383 |
| lmo0026_s_at | GI=16409385 |
| lmo0030_s_at | GI=16409389 |
| lmo0031_s_at | transcriptional regulator LacI family/GI=16409390 |
| lmo0032_s_at | GI=16409391 |
| lmo0034_s_at | GI=16409393 |
| lmo0035_s_at | GI=16409394 |
| lmo0036_s_at | GI=16409395 |
| lmo0039_s_at | GI=16409398 |
| lmo0042_at | GI=16409401 |
| lmo0043_s_at | GI=16409402 |
| lmo0054_s_at | dnaC GI=16409413 |
| lmo0085_s_at | GI=16409444 |
| lmo0087_s_at | GI=16409446 |
| lmo0090_s_at | GI=16409449 |
| lmo0091_s_at | GI=16409450 |
| lmo0092_s_at | GI=16409451 |
| lmo0094_s_at | GI=16409453 |
| lmo0095_s_at | GI=16409454 |
| lmo0104_s_at | GI=16409463 |
| lmo0113_s_at | GI=16409472 |
| lmo0125_s_at | GI=16409484 |
| lmo0136_s_at | GI=16409495 |
| lmo0157_s_at | GI=16409516 |
| lmo0158_s_at | GI=16409517 |
| lmo0159_s_at | putative peptidoglycan bound protein (LPXTG motif)/GI=16409518 |
| lmo0161_s_at | GI=16409520 |
| lmo0162_s_at | GI=16409521 |
| lmo0167_s_at | GI=16409526 |
| lmo0168_at | GI=16409527 |
| lmo0168_x_at | GI=16409527 |
| lmo0174_s_at | GI=16409531 |
| lmo0176_s_at | GI=16409533 |
| lmo0181_s_at | GI=16409538 |
| lmo0183_s_at | GI=16409540 |
| lmo0184_s_at | GI=16409541 |
| lmo0187_s_at | GI=16409544 |
| lmo0188_s_at | ksgA dimethyladenosine transferase (16S rRNA dimethylase)/GI=16409545 |
| lmo0190_s_at | GI=16409547 |
| lmo0191_s_at | GI=16409548 |
| lmo0194_s_at | "ABC transporter, ATP-binding protein"/GI=16409551 |
| lmo0195_s_at | GI=16409552 |
| lmo0198_s_at | gcaD GI=16409563 |
| lmo0206_s_at | GI=16409571 |
| lmo0207_at | GI=16409572 |
| lmo0210_s_at | ldh GI=16409575 |
| lmo0211_s_at | ctc GI=16409576 |
| lmo0212_s_at | GI=16409577 |
| lmo0213_s_at | pth GI=16409578 |
| lmo0215_s_at | GI=16409580 |
| lmo0221_s_at | GI=16409586 |
| lmo0222_s_at | GI=16409587 |
| lmo0223_s_at | cysK GI=16409588 |
| lmo0224_s_at | sul GI=16409589 |
| lmo0225_s_at | folA GI=16409590 |
| lmo0228_s_at | lysS lysyl-tRNA synthetase/GI=16409593 |
| lmo0231_s_at | GI=16409596 |
| lmo0232_s_at | clpC endopeptidase Clp ATP-binding chain C/GI=16409597 |
| lmo0233_s_at | GI=16409598 |
| lmo0236_s_at | GI=16409601 |
| lmo0240_s_at | GI=16409605 |
| lmo0241_s_at | GI=16409606 |
| lmo0242_at | GI=16409607 |
| lmo0242_x_at | GI=16409607 |
| lmo0247_s_at | GI=16409612 |
| lmo0264_s_at | inlE internalin E/GI=16409629 |
| lmo0267_s_at | GI=16409632 |
| lmo0286_s_at | GI=16409651 |
| lmo0287_s_at | GI=16409652 |
| lmo0288_s_at | GI=16409653 |
| lmo0338_s_at | GI=16409716 |
| lmo0341_s_at | GI=16409719 |
| lmo0346_s_at | GI=16409724 |
| lmo0351_s_at | GI=16409729 |
| lmo0352_s_at | GI=16409730 |
| lmo0354_s_at | GI=16409732 |
| lmo0357_s_at | GI=16409735 |
| lmo0359_s_at | GI=16409737 |
| lmo0360_s_at | GI=16409738 |
| lmo0384_s_at | GI=16409762 |
| lmo0385_s_at | GI=16409763 |
| lmo0389_s_at | ltrA low temperature requirement protein A/GI=16409767 |
| lmo0390_s_at | GI=16409768 |
| lmo0393_s_at | GI=16409771 |
| lmo0394_s_at | GI=16409772 |
| lmo0396_s_at | GI=16409774 |
| lmo0397_s_at | GI=16409775 |
| lmo0398_x_at | GI=16409776 |
| lmo0402_s_at | GI=16409780 |
| lmo0411_s_at | GI=16409788 |
| lmo0415_s_at | GI=16409792 |
| lmo0417_s_at | GI=16409794 |
| lmo0420_s_at | GI=16409797 |
| lmo0422_s_at | GI=16409799 |
| lmo0430_s_at | GI=16409807 |
| lmo0434_s_at | inlB Internalin B/GI=16409811 |
| lmo0450_s_at | GI=16409827 |
| lmo0453_s_at | GI=16409830 |
| lmo0456_s_at | GI=16409833 |
| lmo0478_s_at | putative secreted protein/GI=16409854 |
| lmo0495_s_at | GI=16409871 |
| lmo0498_s_at | GI=16409874 |
| lmo0499_s_at | GI=16409875 |
| lmo0507_at | GI=16409883 |
| lmo0507_x_at | GI=16409883 |
| lmo0512_s_at | GI=16409888 |
| lmo0513_s_at | GI=16409889 |
| lmo0514_s_at | GI=16409890 |
| lmo0515_s_at | GI=16409891 |
| lmo0523_s_at | GI=16409899 |
| lmo0524_s_at | GI=16409900 |
| lmo0526_s_at | GI=16409902 |
| lmo0532_x_at | GI=16409908 |
| lmo0534_s_at | GI=16409910 |
| lmo0538_s_at | GI=16409914 |
| lmo0542_s_at | GI=16409918 |
| lmo0545_s_at | GI=16409921 |
| lmo0546_s_at | GI=16409922 |
| lmo0552_s_at | GI=16409928 |
| lmo0554_s_at | GI=16409930 |
| lmo0557_s_at | GI=16409933 |
| lmo0558_s_at | GI=16409934 |
| lmo0563_s_at | hisF GI=16409939 |
| lmo0569_s_at | hisZ histidyl-tRNA synthetase/GI=16409945 |
| lmo0570_s_at | hisJ GI=16409946 |
| lmo0572_s_at | GI=16409948 |
| lmo0573_s_at | GI=16409949 |
| lmo0578_s_at | putative conserved membrane protein/GI=16409954 |
| lmo0583_s_at | GI=16409959 |
| lmo0584_s_at | GI=16409960 |
| lmo0585_s_at | putative secreted protein/GI=16409961 |
| lmo0589_s_at | GI=16409965 |
| lmo0595_s_at | GI=16409971 |
| lmo0600_s_at | GI=16409989 |
| lmo0603_x_at | GI=16409992 |
| lmo0610_s_at | GI=16409999 |
| lmo0612_s_at | GI=16410001 |
| lmo0614_s_at | GI=16410003 |
| lmo0616_s_at | C-terminal domain similar to glycerophosphoryl diester phosphodiesterase/GI=16410005 |
| lmo0617_s_at | GI=16410006 |
| lmo0621_s_at | GI=16410010 |
| lmo0622_x_at | GI=16410011 |
| lmo0623_at | GI=16410012 |
| lmo0624_s_at | GI=16410013 |
| lmo0625_s_at | GI=16410014 |
| lmo0632_s_at | GI=16410021 |
| lmo0634_s_at | GI=16410023 |
| lmo0635_s_at | GI=16410024 |
| lmo0640_s_at | GI=16410029 |
| lmo0642_s_at | GI=16410031 |
| lmo0643_s_at | GI=16410032 |
| lmo0645_s_at | GI=16410034 |
| lmo0646_s_at | GI=16410035 |
| lmo0648_at | GI=16410037 |
| lmo0650_s_at | GI=16410039 |
| lmo0651_s_at | GI=16410040 |
| lmo0652_s_at | GI=16410041 |
| lmo0658_s_at | GI=16410047 |
| lmo0659_s_at | GI=16410048 |
| lmo0660_s_at | GI=16410049 |
| lmo0661_s_at | GI=16410050 |
| lmo0662_s_at | thiD GI=16410051 |
| lmo0664_s_at | GI=16410053 |
| lmo0671_s_at | GI=16410060 |
| lmo0678_s_at | GI=16410067 |
| lmo0681_s_at | GI=16410070 |
| lmo0682_x_at | GI=16410071 |
| lmo0683_at | GI=16410072 |
| lmo0687_s_at | GI=16410076 |
| lmo0688_s_at | GI=16410077 |
| lmo0689_at | GI=16410078 |
| lmo0689_s_at | GI=16410078 |
| lmo0693_s_at | GI=16410082 |
| lmo0696_s_at | GI=16410085 |
| lmo0703_s_at | GI=16410092 |
| lmo0705_s_at | GI=16410094 |
| lmo0707_s_at | GI=16410096 |
| lmo0715_s_at | GI=16410104 |
| lmo0719_at | GI=16410108 |
| lmo0721_s_at | putative fibronectin-binding protein/GI=16410110 |
| lmo0722_s_at | GI=16410111 |
| lmo0724_s_at | GI=16410113 |
| lmo0725_s_at | putative peptidoglycan bound protein (LPXTG motif)/GI=16410114 |
| lmo0727_s_at | GI=16410116 |
| lmo0732_s_at | putative peptidoglycan bound protein (LPXTG motif)/GI=16410121 |
| lmo0735_s_at | GI=16410124 |
| lmo0736_at | GI=16410125 |
| lmo0737_s_at | GI=16410126 |
| lmo0738_s_at | GI=16410127 |
| lmo0743_s_at | GI=16410132 |
| lmo0759_s_at | GI=16410148 |
| lmo0762_s_at | GI=16410151 |
| lmo0763_s_at | GI=16410152 |
| lmo0764_s_at | GI=16410153 |
| lmo0772_at | GI=16410161 |
| lmo0774_s_at | GI=16410163 |
| lmo0776_s_at | GI=16410165 |
| lmo0781_s_at | GI=16410170 |
| lmo0785_s_at | GI=16410174 |
| lmo0791_s_at | GI=16410180 |
| lmo0803_s_at | GI=16410192 |
| lmo0819_s_at | GI=16410208 |
| lmo0825_s_at | GI=16410214 |
| lmo0831_s_at | GI=16410220 |
| lmo0832_s_at | GI=16410221 |
| lmo0833_s_at | GI=16410222 |
| lmo0843_x_at | GI=16410231 |
| lmo0846_s_at | GI=16410234 |
| lmo0847_s_at | GI=16410235 |
| lmo0864_s_at | GI=16410267 |
| lmo0869_s_at | GI=16410272 |
| lmo0872_s_at | GI=16410275 |
| lmo0875_s_at | GI=16410278 |
| lmo0879_s_at | GI=16410282 |
| lmo0880_s_at | GI=16410283 |
| lmo0881_s_at | GI=16410284 |
| lmo0883_s_at | GI=16410286 |
| lmo0884_s_at | GI=16410287 |
| lmo0891_s_at | rsbT GI=16410294 |
| lmo0893_at | rsbV anti-anti-sigma factor (antagonist of RsbW)/GI=16410296 |
| lmo0894_s_at | rsbW sigma-B activity negative regulator RsbW/GI=16410297 |
| lmo0895_s_at | sigB RNA polymerase sigma-37 factor (sigma-B)/GI=16410298 |
| lmo0896_s_at | rsbX Indirect negative regulation of sigma B dependant gene expression (serine phosphatase)/GI=16410299 |
| lmo0898_s_at | GI=16410301 |
| lmo0904_s_at | GI=16410307 |
| lmo0925_s_at | putative membrane protein/GI=16410327 |
| lmo0926_s_at | GI=16410328 |
| lmo0928_s_at | GI=16410330 |
| lmo0931_s_at | GI=16410333 |
| lmo0932_s_at | GI=16410334 |
| lmo0935_at | GI=16410337 |
| lmo0935_x_at | GI=16410337 |
| lmo0938_s_at | GI=16410340 |
| lmo0947_s_at | GI=16410349 |
| lmo0949_s_at | GI=16410351 |
| lmo0950_s_at | GI=16410352 |
| lmo0951_s_at | GI=16410353 |
| lmo0953_at | GI=16410355 |
| lmo0953_x_at | GI=16410355 |
| lmo0963_s_at | GI=16410365 |
| lmo0968_s_at | GI=16410370 |
| lmo0974_s_at | dltA "D-alanine-activating enzyme (dae), D-alanine-D-alanyl carrier protein ligase (dcl)"/GI=16410376 |
| lmo0975_s_at | GI=16410377 |
| lmo0976_s_at | GI=16410378 |
| lmo0979_s_at | GI=16410381 |
| lmo0982_s_at | GI=16410384 |
| lmo0983_s_at | GI=16410385 |
| lmo0984_s_at | GI=16410386 |
| lmo0985_s_at | GI=16410387 |
| lmo0986_s_at | GI=16410388 |
| lmo0987_s_at | GI=16410389 |
| lmo0988_s_at | GI=16410390 |
| lmo0990_s_at | GI=16410392 |
| lmo1000_s_at | GI=16410402 |
| lmo1006_s_at | GI=16410408 |
| lmo1007_s_at | GI=16410409 |
| lmo1011_s_at | GI=16410413 |
| lmo1012_s_at | GI=16410414 |
| lmo1013_s_at | GI=16410415 |
| lmo1022_s_at | GI=16410424 |
| lmo1025_s_at | GI=16410427 |
| lmo1029_s_at | GI=16410431 |
| lmo1030_s_at | GI=16410432 |
| lmo1031_s_at | GI=16410433 |
| lmo1033_s_at | GI=16410435 |
| lmo1041_s_at | GI=16410443 |
| lmo1043_x_at | GI=16410445 |
| lmo1049_s_at | GI=16410451 |
| lmo1050_s_at | GI=16410452 |
| lmo1056_s_at | GI=16410458 |
| lmo1057_s_at | GI=16410459 |
| lmo1059_s_at | GI=16410461 |
| lmo1063_s_at | GI=16410465 |
| lmo1071_s_at | GI=16410473 |
| lmo1136_s_at | GI=16410552 |
| lmo1137_s_at | GI=16410553 |
| lmo1138_s_at | GI=16410554 |
| lmo1146_s_at | GI=16410562 |
| lmo1147_s_at | GI=16410563 |
| lmo1148_s_at | GI=16410564 |
| lmo1149_s_at | GI=16410565 |
| lmo1150_s_at | Regulatory protein similar to Salmonella typhimurium PocR protein/GI=16410566 |
| lmo1152_s_at | GI=16410568 |
| lmo1160_s_at | GI=16410576 |
| lmo1169_s_at | cobD GI=16410585 |
| lmo1170_s_at | GI=16410586 |
| lmo1171_s_at | pduQ GI=16410587 |
| lmo1179_s_at | GI=16410595 |
| lmo1186_s_at | GI=16410602 |
| lmo1193_s_at | GI=16410609 |
| lmo1195_s_at | cbiE GI=16410611 |
| lmo1196_at | GI=16410612 |
| lmo1197_s_at | cbiF GI=16410613 |
| lmo1203_s_at | cbiL GI=16410619 |
| lmo1204_s_at | GI=16410620 |
| lmo1206_s_at | cbiQ GI=16410622 |
| lmo1210_s_at | GI=16410626 |
| lmo1216_s_at | GI=16410632 |
| lmo1217_s_at | GI=16410633 |
| lmo1224_s_at | GI=16410640 |
| lmo1226_s_at | GI=16410642 |
| lmo1231_s_at | GI=16410647 |
| lmo1232_s_at | GI=16410648 |
| lmo1254_x_at | GI=16410670 |
| lmo1262_s_at | GI=16410678 |
| lmo1264_s_at | GI=16410680 |
| lmo1265_s_at | GI=16410681 |
| lmo1266_s_at | GI=16410682 |
| lmo1272_s_at | GI=16410688 |
| lmo1276_s_at | gid GI=16410692 |
| lmo1277_s_at | codV GI=16410693 |
| lmo1280_s_at | codY GI=16410696 |
| lmo1284_s_at | GI=16410700 |
| lmo1290_s_at | GI=16410706 |
| lmo1291_s_at | GI=16410707 |
| lmo1294_s_at | miaA GI=16410710 |
| lmo1296_s_at | GI=16410712 |
| lmo1297_s_at | GI=16410713 |
| lmo1299_s_at | glnA GI=16410715 |
| lmo1301_s_at | GI=16410717 |
| lmo1302_s_at | GI=16410718 |
| lmo1303_s_at | GI=16410719 |
| lmo1304_s_at | GI=16410720 |
| lmo1305_s_at | tkt GI=16410721 |
| lmo1307_s_at | GI=16410723 |
| lmo1308_s_at | GI=16410724 |
| lmo1310_s_at | GI=16410726 |
| lmo1311_s_at | GI=16410727 |
| lmo1312_at | GI=16410728 |
| lmo1317_s_at | GI=16410733 |
| lmo1318_s_at | GI=16410734 |
| lmo1319_s_at | proS prolyl-tRNA synthetase/GI=16410735 |
| lmo1320_s_at | polC GI=16410736 |
| lmo1329_s_at | ribC GI=16410745 |
| lmo1331_s_at | pnpA polynucleotide phosphorylase (PNPase)/GI=16410747 |
| lmo1332_s_at | GI=16410748 |
| lmo1336_s_at | GI=16410752 |
| lmo1337_s_at | GI=16410753 |
| lmo1339_s_at | GI=16410755 |
| lmo1349_s_at | GI=16410765 |
| lmo1350_s_at | GI=16410766 |
| lmo1352_s_at | GI=16410768 |
| lmo1353_s_at | GI=16410769 |
| lmo1354_s_at | GI=16410770 |
| lmo1355_s_at | efp GI=16410771 |
| lmo1356_s_at | GI=16410772 |
| lmo1357_s_at | acetyl-CoA carboxylase subunit (biotin carboxylase subunit)/GI=16410773 |
| lmo1361_s_at | GI=16410777 |
| lmo1363_s_at | GI=16410779 |
| lmo1365_s_at | tktB GI=16410781 |
| lmo1366_s_at | GI=16410782 |
| lmo1368_s_at | recN DNA repair and genetic recombination/GI=16410784 |
| lmo1369_s_at | GI=16410785 |
| lmo1372_s_at | GI=16410788 |
| lmo1373_s_at | GI=16410789 |
| lmo1377_at | lisR two-component response regulator/GI=16410793 |
| lmo1379_x_at | GI=16410795 |
| lmo1382_s_at | GI=16410798 |
| lmo1383_s_at | GI=16410799 |
| lmo1385_s_at | GI=16410801 |
| lmo1386_s_at | GI=16410815 |
| lmo1387_at | GI=16410816 |
| lmo1387_s_at | GI=16410816 |
| lmo1390_s_at | GI=16410819 |
| lmo1391_s_at | GI=16410820 |
| lmo1393_s_at | GI=16410822 |
| lmo1394_s_at | GI=16410823 |
| lmo1395_s_at | GI=16410824 |
| lmo1396_s_at | GI=16410825 |
| lmo1397_s_at | cinA GI=16410826 |
| lmo1406_s_at | pflB pyruvate formate-lyase/GI=16410835 |
| lmo1409_s_at | GI=16410838 |
| lmo1410_s_at | GI=16410839 |
| lmo1411_at | GI=16410840 |
| lmo1411_x_at | GI=16410840 |
| lmo1412_s_at | modulates DNA topology/GI=16410841 |
| lmo1413_s_at | putative peptidoglycan bound protein (LPXTG motif)/GI=16410842 |
| lmo1414_s_at | GI=16410843 |
| lmo1416_s_at | GI=16410845 |
| lmo1419_s_at | GI=16410848 |
| lmo1421_s_at | GI=16410850 |
| lmo1422_s_at | GI=16410851 |
| lmo1423_s_at | GI=16410852 |
| lmo1424_at | GI=16410853 |
| lmo1424_s_at | GI=16410853 |
| lmo1426_s_at | opuCC GI=16410855 |
| lmo1429_s_at | GI=16410858 |
| lmo1430_s_at | GI=16410859 |
| lmo1433_s_at | GI=16410862 |
| lmo1435_s_at | GI=16410864 |
| lmo1436_s_at | GI=16410865 |
| lmo1440_s_at | GI=16410869 |
| lmo1441_s_at | GI=16410870 |
| lmo1442_s_at | GI=16410871 |
| lmo1443_s_at | GI=16410872 |
| lmo1444_s_at | GI=16410873 |
| lmo1445_s_at | zurR transcriptional regulator ZurR (ferric uptake regulation)/GI=16410874 |
| lmo1446_x_at | zurM "metal (zinc) transport protein (ABC transporter, permease protein)"/GI=16410875 |
| lmo1449_s_at | GI=16410878 |
| lmo1450_s_at | GI=16410879 |
| lmo1452_s_at | GI=16410881 |
| lmo1456_s_at | GI=16410885 |
| lmo1461_s_at | GI=16410890 |
| lmo1466_s_at | GI=16410895 |
| lmo1467_s_at | GI=16410896 |
| lmo1470_s_at | GI=16410899 |
| lmo1471_s_at | GI=16410900 |
| lmo1474_s_at | grpE heat shock protein GrpE/GI=16410903 |
| lmo1481_s_at | GI=16410910 |
| lmo1484_s_at | comEA GI=16410913 |
| lmo1489_at | GI=16410918 |
| lmo1491_s_at | GI=16410920 |
| lmo1499_s_at | GI=16410928 |
| lmo1500_s_at | GI=16410929 |
| lmo1504_s_at | alaS alanyl-tRNA synthetase/GI=16410933 |
| lmo1505_s_at | GI=16410934 |
| lmo1506_s_at | GI=16410935 |
| lmo1507_s_at | GI=16410936 |
| lmo1508_s_at | GI=16410937 |
| lmo1509_s_at | GI=16410938 |
| lmo1512_s_at | GI=16410941 |
| lmo1514_s_at | GI=16410943 |
| lmo1515_s_at | GI=16410944 |
| lmo1516_s_at | GI=16410945 |
| lmo1519_s_at | aspS aspartyl-tRNA synthetase/GI=16410948 |
| lmo1521_s_at | GI=16410950 |
| lmo1522_s_at | GI=16410951 |
| lmo1523_s_at | relA GI=16410952 |
| lmo1524_at | apt GI=16410953 |
| lmo1525_s_at | GI=16410954 |
| lmo1530_s_at | GI=16410959 |
| lmo1532_s_at | ruvB GI=16410961 |
| lmo1533_s_at | ruvA GI=16410962 |
| lmo1534_s_at | GI=16410963 |
| lmo1539_s_at | GI=16410968 |
| lmo1549_s_at | GI=16410978 |
| lmo1555_s_at | GI=16410984 |
| lmo1557_s_at | hemA GI=16410986 |
| lmo1563_s_at | GI=16410992 |
| lmo1566_s_at | citC GI=16410995 |
| lmo1568_s_at | GI=16410997 |
| lmo1569_at | GI=16410998 |
| lmo1570_s_at | pykA GI=16410999 |
| lmo1578_s_at | GI=16411007 |
| lmo1582_s_at | GI=16411011 |
| lmo1583_s_at | GI=16411012 |
| lmo1584_s_at | GI=16411013 |
| lmo1586_s_at | GI=16411015 |
| lmo1587_s_at | argF GI=16411016 |
| lmo1588_s_at | argD GI=16411017 |
| lmo1589_s_at | argB GI=16411018 |
| lmo1590_s_at | argJ GI=16411019 |
| lmo1591_s_at | argC GI=16411020 |
| lmo1592_s_at | GI=16411021 |
| lmo1604_s_at | GI=16411033 |
| lmo1607_s_at | pheT GI=16411043 |
| lmo1608_s_at | GI=16411044 |
| lmo1609_s_at | GI=16411045 |
| lmo1610_s_at | GI=16411046 |
| lmo1615_s_at | GI=16411051 |
| lmo1616_at | GI=16411052 |
| lmo1617_s_at | GI=16411053 |
| lmo1619_s_at | daaA D-Amino Acid Aminotransferase/GI=16411055 |
| lmo1623_s_at | GI=16411059 |
| lmo1627_s_at | trpA GI=16411063 |
| lmo1630_s_at | trpC GI=16411066 |
| lmo1635_s_at | GI=16411071 |
| lmo1637_s_at | GI=16411073 |
| lmo1640_s_at | GI=16411076 |
| lmo1647_s_at | GI=16411083 |
| lmo1652_s_at | GI=16411088 |
| lmo1653_s_at | putative cellsurface protein/GI=16411089 |
| lmo1654_x_at | putative cellsurface protein/GI=16411090 |
| lmo1657_s_at | tsf translation elongation factor/GI=16411093 |
| lmo1688_s_at | GI=16411142 |
| lmo1689_s_at | GI=16411143 |
| lmo1692_s_at | GI=16411146 |
| lmo1694_s_at | GI=16411148 |
| lmo1695_s_at | GI=16411149 |
| lmo1697_s_at | GI=16411151 |
| lmo1698_s_at | GI=16411152 |
| lmo1704_s_at | GI=16411158 |
| lmo1706_s_at | GI=16411160 |
| lmo1707_s_at | GI=16411161 |
| lmo1723_s_at | GI=16411177 |
| lmo1724_s_at | GI=16411178 |
| lmo1726_s_at | GI=16411180 |
| lmo1738_s_at | GI=16411192 |
| lmo1741_s_at | GI=16411195 |
| lmo1742_s_at | adeC GI=16411196 |
| lmo1752_s_at | GI=16411206 |
| lmo1754_s_at | gatB glutamyl-tRNA(Gln) amidotransferase (subunit B)/GI=16411208 |
| lmo1755_s_at | gatA glutamyl-tRNA(Gln) amidotransferase (subunit A)/GI=16411209 |
| lmo1756_x_at | gatC glutamyl-tRNA(Gln) amidotransferase (subunit C)/GI=16411210 |
| lmo1758_s_at | GI=16411212 |
| lmo1760_s_at | GI=16411214 |
| lmo1761_s_at | GI=16411215 |
| lmo1763_at | GI=16411217 |
| lmo1777_s_at | GI=16411231 |
| lmo1778_s_at | GI=16411232 |
| lmo1779_s_at | GI=16411233 |
| lmo1780_s_at | GI=16411234 |
| lmo1781_s_at | GI=16411235 |
| lmo1782_s_at | GI=16411236 |
| lmo1789_s_at | GI=16411243 |
| lmo1790_s_at | GI=16411244 |
| lmo1792_at | trmD GI=16411246 |
| lmo1793_s_at | GI=16411247 |
| lmo1802_at | GI=16411256 |
| lmo1809_s_at | plsX GI=16411263 |
| lmo1822_s_at | GI=16411276 |
| lmo1829_s_at | GI=16411283 |
| lmo1834_s_at | pyrDII GI=16411288 |
| lmo1836_s_at | pyrAa GI=16411290 |
| lmo1837_s_at | pyrC GI=16411291 |
| lmo1840_s_at | pyrR GI=16411294 |
| lmo1846_s_at | GI=16411300 |
| lmo1849_s_at | GI=16411303 |
| lmo1861_s_at | GI=16411315 |
| lmo1865_s_at | GI=16411319 |
| lmo1866_s_at | GI=16411320 |
| lmo1874_s_at | GI=16411328 |
| lmo1880_s_at | GI=16411333 |
| lmo1881_s_at | GI=16411334 |
| lmo1882_s_at | GI=16411335 |
| lmo1883_s_at | GI=16411336 |
| lmo1887_s_at | GI=16411340 |
| lmo1892_s_at | pbpA GI=16411345 |
| lmo1893_x_at | GI=16411346 |
| lmo1901_s_at | panC GI=16411354 |
| lmo1902_s_at | panB GI=16411355 |
| lmo1903_s_at | GI=16411356 |
| lmo1904_s_at | birA GI=16411357 |
| lmo1905_s_at | cca GI=16411358 |
| lmo1906_s_at | GI=16411359 |
| lmo1907_s_at | dapB GI=16411360 |
| lmo1908_s_at | GI=16411361 |
| lmo1909_s_at | GI=16411362 |
| lmo1911_s_at | GI=16411364 |
| lmo1912_s_at | GI=16411365 |
| lmo1916_s_at | GI=16411369 |
| lmo1917_s_at | pflA GI=16411370 |
| lmo1920_s_at | GI=16411373 |
| lmo1921_at | GI=16411374 |
| lmo1921_x_at | GI=16411374 |
| lmo1924_s_at | tyrA GI=16411377 |
| lmo1926_s_at | GI=16411379 |
| lmo1927_s_at | aroB GI=16411380 |
| lmo1928_s_at | aroF GI=16411381 |
| lmo1929_s_at | ndk GI=16411382 |
| lmo1942_s_at | recS GI=16411395 |
| lmo1943_s_at | GI=16411396 |
| lmo1945_s_at | GI=16411398 |
| lmo1946_at | GI=16411399 |
| lmo1949_s_at | GI=16411402 |
| lmo1950_s_at | GI=16411403 |
| lmo1951_s_at | GI=16411404 |
| lmo1952_s_at | lysA GI=16411405 |
| lmo1953_s_at | pnp GI=16411406 |
| lmo1954_s_at | drm GI=16411407 |
| lmo1955_s_at | GI=16411408 |
| lmo1959_s_at | GI=16411412 |
| lmo1961_s_at | GI=16411414 |
| lmo1962_s_at | GI=16411415 |
| lmo1963_s_at | GI=16411416 |
| lmo1965_s_at | GI=16411418 |
| lmo1968_s_at | GI=16411421 |
| lmo1972_at | GI=16411425 |
| lmo1973_s_at | GI=16411426 |
| lmo1974_s_at | GI=16411427 |
| lmo1977_s_at | GI=16411430 |
| lmo1978_s_at | GI=16411431 |
| lmo1980_x_at | GI=16411433 |
| lmo1981_s_at | GI=16411434 |
| lmo1982_s_at | GI=16411435 |
| lmo1987_s_at | leuA GI=16411440 |
| lmo1990_s_at | leuD GI=16411443 |
| lmo1999_s_at | GI=16411452 |
| lmo2000_s_at | GI=16411453 |
| lmo2001_s_at | GI=16411454 |
| lmo2008_s_at | GI=16411461 |
| lmo2009_s_at | GI=16411462 |
| lmo2010_s_at | GI=16411463 |
| lmo2011_s_at | GI=16411464 |
| lmo2012_s_at | GI=16411465 |
| lmo2034_s_at | divIB GI=16411504 |
| lmo2035_s_at | murG GI=16411505 |
| lmo2036_s_at | murD GI=16411506 |
| lmo2038_s_at | murE GI=16411508 |
| lmo2043_s_at | GI=16411513 |
| lmo2044_s_at | GI=16411514 |
| lmo2046_s_at | GI=16411516 |
| lmo2057_s_at | ctaB GI=16411527 |
| lmo2059_s_at | GI=16411529 |
| lmo2060_s_at | GI=16411530 |
| lmo2061_s_at | GI=16411531 |
| lmo2062_s_at | GI=16411532 |
| lmo2065_s_at | GI=16411535 |
| lmo2067_s_at | GI=16411537 |
| lmo2070_s_at | GI=16411540 |
| lmo2074_s_at | GI=16411544 |
| lmo2075_s_at | GI=16411545 |
| lmo2077_s_at | GI=16411547 |
| lmo2078_s_at | GI=16411548 |
| lmo2082_s_at | GI=16411552 |
| lmo2084_s_at | GI=16411554 |
| lmo2085_s_at | putative peptidoglycan bound protein (LPXTG motif)/GI=16411555 |
| lmo2087_s_at | GI=16411557 |
| lmo2094_s_at | GI=16411564 |
| lmo2096_s_at | GI=16411566 |
| lmo2099_s_at | GI=16411569 |
| lmo2106_s_at | GI=16411576 |
| lmo2107_s_at | GI=16411577 |
| lmo2109_s_at | GI=16411579 |
| lmo2110_s_at | GI=16411580 |
| lmo2114_s_at | GI=16411584 |
| lmo2116_s_at | GI=16411586 |
| lmo2117_s_at | GI=16411587 |
| lmo2123_s_at | GI=16411593 |
| lmo2124_s_at | GI=16411594 |
| lmo2126_s_at | GI=16411596 |
| lmo2127_s_at | GI=16411597 |
| lmo2128_s_at | GI=16411598 |
| lmo2132_s_at | GI=16411602 |
| lmo2132_x_at | GI=16411602 |
| lmo2133_s_at | GI=16411603 |
| lmo2134_s_at | GI=16411604 |
| lmo2139_s_at | GI=16411609 |
| lmo2140_s_at | GI=16411610 |
| lmo2141_s_at | GI=16411611 |
| lmo2142_s_at | GI=16411612 |
| lmo2143_s_at | GI=16411613 |
| lmo2144_s_at | GI=16411614 |
| lmo2146_s_at | GI=16411616 |
| lmo2159_s_at | GI=16411629 |
| lmo2166_s_at | GI=16411636 |
| lmo2167_s_at | GI=16411637 |
| lmo2170_s_at | GI=16411640 |
| lmo2171_s_at | GI=16411641 |
| lmo2173_s_at | GI=16411643 |
| lmo2174_s_at | GI=16411644 |
| lmo2176_s_at | GI=16411646 |
| lmo2177_s_at | GI=16411647 |
| lmo2179_s_at | putative peptidoglycan bound protein (LPXTG motif)/GI=16411649 |
| lmo2181_s_at | GI=16411651 |
| lmo2182_s_at | GI=16411652 |
| lmo2184_s_at | GI=16411654 |
| lmo2186_s_at | GI=16411656 |
| lmo2187_at | GI=16411657 |
| lmo2188_s_at | GI=16411658 |
| lmo2189_s_at | GI=16411659 |
| lmo2197_s_at | GI=16411667 |
| lmo2198_s_at | trpS tryptophanyl-tRNA synthetase/GI=16411668 |
| lmo2200_s_at | GI=16411670 |
| lmo2203_s_at | GI=16411673 |
| lmo2206_s_at | clpB GI=16411676 |
| lmo2208_s_at | GI=16411678 |
| lmo2209_s_at | GI=16411679 |
| lmo2211_s_at | hemH GI=16411681 |
| lmo2214_s_at | GI=16411684 |
| lmo2218_s_at | GI=16411688 |
| lmo2224_s_at | GI=16411694 |
| lmo2225_s_at | citG GI=16411695 |
| lmo2226_s_at | GI=16411696 |
| lmo2227_s_at | GI=16411697 |
| lmo2230_at | GI=16411700 |
| lmo2231_s_at | GI=16411701 |
| lmo2232_s_at | GI=16411702 |
| lmo2239_s_at | GI=16411709 |
| lmo2240_s_at | GI=16411710 |
| lmo2241_s_at | GI=16411711 |
| lmo2243_s_at | GI=16411713 |
| lmo2245_s_at | GI=16411715 |
| lmo2247_s_at | GI=16411717 |
| lmo2254_s_at | GI=16411724 |
| lmo2257_at | GI=16411727 |
| lmo2258_at | GI=16411728 |
| lmo2258_x_at | GI=16411728 |
| lmo2335_s_at | fruA GI=16411823 |
| lmo2337_s_at | GI=16411825 |
| lmo2339_s_at | GI=16411827 |
| lmo2341_s_at | GI=16411829 |
| lmo2348_s_at | GI=16411836 |
| lmo2354_s_at | GI=16411842 |
| lmo2359_s_at | GI=16411847 |
| lmo2363_s_at | GI=16411851 |
| lmo2368_s_at | GI=16411856 |
| lmo2371_s_at | GI=16411859 |
| lmo2372_s_at | GI=16411860 |
| lmo2381_s_at | GI=16411869 |
| lmo2382_s_at | GI=16411870 |
| lmo2386_s_at | GI=16411874 |
| lmo2387_s_at | GI=16411875 |
| lmo2391_s_at | GI=16411879 |
| lmo2399_s_at | GI=16411887 |
| lmo2400_s_at | GI=16411888 |
| lmo2403_s_at | GI=16411891 |
| lmo2406_s_at | GI=16411894 |
| lmo2418_s_at | GI=16411906 |
| lmo2424_s_at | GI=16411912 |
| lmo2426_at | GI=16411914 |
| lmo2427_s_at | GI=16411915 |
| lmo2434_s_at | GI=16411922 |
| lmo2435_s_at | GI=16411923 |
| lmo2436_at | GI=16411924 |
| lmo2443_s_at | GI=16411931 |
| lmo2453_s_at | GI=16411941 |
| lmo2460_s_at | GI=16411948 |
| lmo2461_s_at | sigL RNA polymerase sigma-54 factor (sigma-L)/GI=16411949 |
| lmo2462_s_at | GI=16411950 |
| lmo2464_x_at | GI=16411952 |
| lmo2466_s_at | GI=16411954 |
| lmo2473_s_at | GI=16411961 |
| lmo2474_s_at | GI=16411962 |
| lmo2477_s_at | galE UDP-glucose 4-epimerase/GI=16411965 |
| lmo2480_s_at | GI=16411968 |
| lmo2481_x_at | GI=16411969 |
| lmo2482_s_at | lgt GI=16411970 |
| lmo2485_at | GI=16411973 |
| lmo2485_x_at | GI=16411973 |
| lmo2487_s_at | GI=16411975 |
| lmo2488_s_at | uvrA excinuclease ABC (subunit A)/GI=16411976 |
| lmo2492_s_at | GI=16411980 |
| lmo2494_s_at | GI=16411982 |
| lmo2495_s_at | GI=16411983 |
| lmo2498_s_at | GI=16411986 |
| lmo2503_s_at | GI=16411991 |
| lmo2508_at | GI=16411996 |
| lmo2511_s_at | GI=16411999 |
| lmo2512_s_at | comFC GI=16412000 |
| lmo2513_s_at | comFA GI=16412001 |
| lmo2514_s_at | GI=16412002 |
| lmo2516_s_at | GI=16412004 |
| lmo2521_s_at | GI=16412009 |
| lmo2526_x_at | murA UDP-N-acetylglucosamine 1-carboxyvinyltransferase/GI=16412014 |
| lmo2529_s_at | atpD GI=16412017 |
| lmo2541_s_at | GI=16412029 |
| lmo2553_s_at | GI=16412041 |
| lmo2554_s_at | GI=16412042 |
| lmo2557_s_at | GI=16412045 |
| lmo2559_s_at | pyrG GI=16412047 |
| lmo2562_s_at | GI=16412050 |
| lmo2563_s_at | GI=16412051 |
| lmo2565_s_at | GI=16412053 |
| lmo2566_s_at | GI=16412054 |
| lmo2569_s_at | GI=16412057 |
| lmo2571_s_at | GI=16412059 |
| lmo2572_s_at | GI=16412060 |
| lmo2574_x_at | GI=16412062 |
| lmo2575_s_at | GI=16412063 |
| lmo2577_s_at | GI=16412065 |
| lmo2578_s_at | GI=16412066 |
| lmo2581_s_at | GI=16412069 |
| lmo2582_s_at | GI=16412070 |
| lmo2585_s_at | GI=16412073 |
| lmo2592_s_at | GI=16412080 |
| lmo2601_s_at | GI=16412089 |
| lmo2602_s_at | GI=16412090 |
| lmo2603_s_at | GI=16412091 |
| lmo2604_s_at | GI=16412092 |
| lmo2634_s_at | GI=16412122 |
| lmo2635_s_at | GI=16412123 |
| lmo2636_s_at | GI=16412124 |
| lmo2641_s_at | GI=16412129 |
| lmo2643_s_at | GI=16412131 |
| lmo2647_s_at | GI=16412147 |
| lmo2648_s_at | GI=16412148 |
| lmo2650_at | GI=16412150 |
| lmo2651_s_at | GI=16412151 |
| lmo2658_s_at | GI=16412158 |
| lmo2659_s_at | GI=16412159 |
| lmo2662_s_at | GI=16412162 |
| lmo2669_s_at | GI=16412169 |
| lmo2679_s_at | GI=16412179 |
| lmo2687_s_at | GI=16412187 |
| lmo2694_s_at | GI=16412194 |
| lmo2700_s_at | GI=16412200 |
| lmo2702_s_at | recR GI=16412202 |
| lmo2705_s_at | GI=16412205 |
| lmo2710_s_at | GI=16412210 |
| lmo2712_s_at | GI=16412212 |
| lmo2715_s_at | cydD GI=16412215 |
| lmo2716_s_at | cydC GI=16412216 |
| lmo2719_s_at | GI=16412219 |
| lmo2731_s_at | GI=16412231 |
| lmo2741_s_at | GI=16412241 |
| lmo2742_s_at | GI=16412242 |
| lmo2743_s_at | GI=16412243 |
| lmo2745_s_at | GI=16412245 |
| lmo2746_s_at | GI=16412246 |
| lmo2753_s_at | GI=16412253 |
| lmo2757_s_at | GI=16412257 |
| lmo2758_s_at | guaB GI=16412258 |
| lmo2769_s_at | GI=16412269 |
| lmo2785_s_at | kat catalase/GI=16412285 |
| lmo2787_s_at | bvrB beta-glucoside-specific phosphotransferase enzyme II ABC component/GI=16412287 |
| lmo2788_s_at | bvrA transcription antiterminator/GI=16412288 |
| lmo2789_x_at | GI=16412289 |
| lmo2790_s_at | parB Partition protein ParB homolg/GI=16412290 |
| lmo2792_x_at | GI=16412292 |
| lmo2795_s_at | GI=16412295 |
| lmo2803_s_at | GI=16412303 |
| lmo2807_s_at | GI=16412307 |
| lmo2808_s_at | GI=16412308 |
| lmo2814_s_at | GI=16412314 |
| lmo2818_s_at | GI=16412318 |
| lmo2819_s_at | GI=16412319 |
| lmo2824_s_at | GI=16412324 |
| lmo2833_s_at | GI=16412333 |
| lmo2842_s_at | GI=16412342 |
| lmo2852_s_at | GI=16412352 |
| LMOf6854_0090_x_at | D-isomer specific 2-hydroxyacid dehydrogenase family protein/GI=47016493 |
| LMOf6854_0097_s_at | hypothetical protein/GI=47016500 |
| LMOf6854_0139_at | conserved hypothetical protein/GI=47016542 |
| LMOf6854_0147_s_at | acetyltransferase, GNAT family/GI=47016550 |
| LMOf6854_0182_s_at | ISLmo1, transposase OrfB, N-terminus/GI=47014654 |
| LMOf6854_0183_s_at | ISLmo1, transposase OrfB, C-terminus/GI=47015415 |
| LMOf6854_0236_s_at | folK 2-amino-4-hydroxy-6- hydroxymethyldihydropteridine pyrophosphokinase/GI=47014366 |
| LMOf6854_0248_s_at | gltX glutamyl-tRNA synthetase/GI=47014205 |
| LMOf6854_0276_s_at | inlD internalin D/GI=47015115 |
| LMOf6854_0278_s_at | peptidase, M20M25M40 family/GI=47015117 |
| LMOf6854_0392_at | oxidoreductase, YhhX family/GI=47014941 |
| LMOf6854_0392_s_at | oxidoreductase, YhhX family/GI=47014941 |
| LMOf6854_0392_x_at | oxidoreductase, YhhX family/GI=47014941 |
| LMOf6854_0435_s_at | hypothetical protein/GI=47015048 |
| LMOf6854_0436_x_at | PTS system, IIA component, putative/GI=47015049 |
| LMOf6854_0493_s_at | hypothetical protein/GI=47016826 |
| LMOf6854_0509_s_at | probable secreted protein lmo0477/GI=47016842 |
| LMOf6854_0512_at | hypothetical protein/GI=47016845 |
| LMOf6854_0514_s_at | antigen, putative/GI=47016847 |
| LMOf6854_0571_s_at | conserved hypothetical protein/GI=47016904 |
| LMOf6854_0573_x_at | conserved hypothetical protein/GI=47016906 |
| LMOf6854_0607_s_at | hisB imidazoleglycerol-phosphate dehydratase/GI=47016940 |
| LMOf6854_0616_s_at | transcriptional regulator, GntR family/GI=47016949 |
| LMOf6854_0632_s_at | membrane protein, putative/GI=47014749 |
| LMOf6854_0639_s_at | BioY family protein/GI=47014756 |
| LMOf6854_0643_x_at | conserved hypothetical protein/GI=47014013 |
| LMOf6854_0685_s_at | hypothetical protein/GI=47015353 |
| LMOf6854_0688_s_at | amino acid permease family protein/GI=47016278 |
| LMOf6854_0703_s_at | transposase OrfA, IS3 family, putative, POINT MUTATION/GI=47016293 |
| LMOf6854_0739_s_at | cheA chemotaxis protein CheA/GI=47016329 |
| LMOf6854_0755_x_at | flagellar protein FliS, putative/GI=47016345 |
| LMOf6854_0829_s_at | sigma-54 dependent transcriptional regulator/GI=47014295 |
| LMOf6854_0832_s_at | BadFBadGBcrABcrD ATPase family/GI=47014298 |
| LMOf6854_0864_x_at | conserved hypothetical protein/GI=47016707 |
| LMOf6854_0879_s_at | conserved hypothetical protein/GI=47016722 |
| LMOf6854_0882_s_at | ABC transporter, ATP-binding protein/GI=47016725 |
| LMOf6854_0884_s_at | tetA tetracycline resistance protein/GI=47016727 |
| LMOf6854_0911_at | hypothetical protein/GI=47016754 |
| LMOf6854_0964_s_at | beta-glucosidase/GI=47016807 |
| LMOf6854_0992_s_at | lipoprotein, putative/GI=47015377 |
| LMOf6854_1021_s_at | dltB dltB protein/GI=47015406 |
| LMOf6854_1023_at | conserved hypothetical protein/GI=47015408 |
| LMOf6854_1038_s_at | transcriptional regulator, MarR family/GI=47015279 |
| LMOf6854_1056_x_at | conserved hypothetical protein/GI=47015297 |
| LMOf6854_1068_s_at | conserved hypothetical protein/GI=47015309 |
| LMOf6854_1072_s_at | TrkA domain protein/GI=47015313 |
| LMOf6854_1093_x_at | mobB molybdopterin-guanine dinucleotide biosynthesis protein B/GI=47015479 |
| LMOf6854_1116_at | hypothetical protein/GI=47015502 |
| LMOf6854_1116_x_at | hypothetical protein/GI=47015502 |
| LMOf6854_1165_s_at | conserved hypothetical protein/GI=47016586 |
| LMOf6854_1173_s_at | conserved hypothetical protein/GI=47016594 |
| LMOf6854_1174_s_at | conserved hypothetical protein/GI=47016595 |
| LMOf6854_1203_s_at | pduP CoA-dependent propionaldehyde dehydrogenase/GI=47016624 |
| LMOf6854_1204_s_at | pduQ propanol dehydrogenase/GI=47016625 |
| LMOf6854_1251_s_at | conserved hypothetical protein/GI=47016672 |
| LMOf6854_1254_s_at | N-acetylmuramoyl-L-alanine amidase, family 4/GI=47016675 |
| LMOf6854_1295_at | conserved hypothetical protein/GI=47014582 |
| LMOf6854_1295_x_at | conserved hypothetical protein/GI=47014582 |
| LMOf6854_1316_s_at | topA DNA topoisomerase I/GI=47014217 |
| LMOf6854_1317_s_at | topA DNA topoisomerase I/GI=47014084 |
| LMOf6854_1364_s_at | conserved hypothetical protein/GI=47015652 |
| LMOf6854_1435_s_at | conserved hypothetical protein/GI=47014178 |
| LMOf6854_1441_s_at | recA recA protein/GI=47016170 |
| LMOf6854_1521_s_at | oxygen-independent coproporphyrinogen III oxidase, putative/GI=47016250 |
| LMOf6854_1527_s_at | comE operon protein 3, putative/GI=47016256 |
| LMOf6854_1557_x_at | conserved hypothetical protein/GI=47014422 |
| LMOf6854_1561_s_at | rrf2 rrf2 protein/GI=47014426 |
| LMOf6854_1562_s_at | Rrf2 family protein/GI=47014594 |
| LMOf6854_1564_x_at | nitrogen regulatory protein P-II/GI=47014596 |
| LMOf6854_1584_s_at | pheA prephenate dehydratase/GI=47013919 |
| LMOf6854_1592_s_at | ribonuclease G/GI=47014890 |
| LMOf6854_1598_s_at | hypothetical protein/GI=47014896 |
| LMOf6854_1600_s_at | type IV leader peptidase family protein/GI=47014898 |
| LMOf6854_1626_s_at | dnaE DNA polymerase III, alpha subunit/GI=47014820 |
| LMOf6854_1628_s_at | CBS domain protein/GI=47014822 |
| LMOf6854_1650_s_at | S4 domain protein/GI=47013862 |
| LMOf6854_1673_s_at | dipeptidase, putative subfamily/GI=47013870 |
| LMOf6854_1674_s_at | dipeptidase/GI=47013878 |
| LMOf6854_1676_s_at | conserved hypothetical protein/GI=47013880 |
| LMOf6854_1679_s_at | polysaccharide biosynthesis family protein/GI=47013944 |
| LMOf6854_1689_s_at | conserved hypothetical protein/GI=47014164 |
| LMOf6854_1695_s_at | conserved hypothetical protein/GI=47014615 |
| LMOf6854_1702_s_at | exonuclease, SbcC family/GI=47014622 |
| LMOf6854_1751_s_at | conserved hypothetical protein/GI=47013995 |
| LMOf6854_1756_s_at | methyl-accepting chemotaxis protein/GI=47013964 |
| LMOf6854_1759_s_at | conserved hypothetical protein/GI=47013967 |
| LMOf6854_1760_s_at | glyoxalase family protein/GI=47013968 |
| LMOf6854_1764_s_at | deoxynucleoside kinase family protein/GI=47014018 |
| LMOf6854_1767_s_at | aminoglycoside N3-acetyltransferase/GI=47014021 |
| LMOf6854_1871_s_at | recG ATP-dependent DNA helicase RecG/GI=47015064 |
| LMOf6854_1902_s_at | sigma-B regulator RsbR, putative/GI=47013886 |
| LMOf6854_1903_x_at | STAS domain protein/GI=47013930 |
| LMOf6854_1929_x_at | conserved hypothetical protein/GI=47014724 |
| LMOf6854_1936_s_at | fhs formate--tetrahydrofolate ligase/GI=47014731 |
| LMOf6854_1938_s_at | 5'-3' exonuclease family protein/GI=47013904 |
| LMOf6854_1992_s_at | heptaprenyl diphosphate synthase component I, putative/GI=47013899 |
| LMOf6854_2016_x_at | transcriptional regulator, Fur family/GI=47014544 |
| LMOf6854_2018_s_at | iron compound ABC transporter, permease protein/GI=47013902 |
| LMOf6854_2021_x_at | iron compound ABC transporter, ATP-binding protein/GI=47014989 |
| LMOf6854_2054_s_at | transcriptional regulator, LacI family/GI=47013913 |
| LMOf6854_2056_s_at | transcriptional regulator, putative/GI=47013915 |
| LMOf6854_2064_s_at | transcriptional regulator, GntR family/GI=47014967 |
| LMOf6854_2066_x_at | hypothetical protein/GI=47014969 |
| LMOf6854_2128_x_at | conserved hypothetical protein/GI=47014151 |
| LMOf6854_2131_s_at | CAAX amino terminal protease family protein/GI=47015783 |
| LMOf6854_2169_s_at | conserved hypothetical protein/GI=47015821 |
| LMOf6854_2280_s_at | ABC transporter, ATP-binding protein/GI=47014279 |
| LMOf6854_2286_s_at | conserved hypothetical protein/GI=47014057 |
| LMOf6854_2287_s_at | SerThr protein phosphatase family protein/GI=47014758 |
| LMOf6854_2300_s_at | NADH oxidase, putative/GI=47014771 |
| LMOf6854_2405_s_at | amino acid ABC transporter, ATP-binding protein/GI=47015641 |
| LMOf6854_2415_s_at | transporter, putative/GI=47014283 |
| LMOf6854_2439_s_at | Na+H+ antiporter component A, putative/GI=47015734 |
| LMOf6854_2464_x_at | conserved hypothetical protein/GI=47015759 |
| LMOf6854_2480_s_at | ABC transporter, substrate-binding protein/GI=47015775 |
| LMOf6854_2635_x_at | conserved hypothetical protein/GI=47014523 |
| LMOf6854_2641_s_at | internalin H/GI=47014848 |
| LMOf6854_2719_s_at | cobalt ABC transporter, permease protein/GI=47014429 |
| LMOf6854_2752_s_at | pyridine nucleotide-disulfide oxidoreductase family protein/GI=47014488 |
| LMOf6854_2759_s_at | conserved hypothetical protein/GI=47014495 |
| LMOf6854_2766_s_at | PTS system, IIA component, putative/GI=47014502 |
| LMOf6854_2776_s_at | transketolase/GI=47016074 |
| LMOf6854_2797_s_at | kdpB K+-transporting ATPase, B subunit/GI=47016095 |
| LMOf6854_2849_s_at | HAD-superfamily hydrolase, subfamily IA, variant 1/GI=47016147 |
| LMOf6854_2871_s_at | ABC transporter, ATP-bindingpermease protein/GI=47015990 |
| LMOf6854_2897_s_at | ychF GTP-binding protein YchF/GI=47016016 |
| LMOf6854_2916_s_at | hydrolase, haloacid dehalogenase-like family/GI=47016035 |
| LMOf6854_2921_s_at | conserved hypothetical protein/GI=47016040 |
| LMOG_00087_x_at | conserved hypothetical protein |
| LMOG_00443_at | predicted protein |
| LMOG_00454_at | predicted protein |
| LMOG_00709_at | predicted protein |
| LMOG_00709_x_at | predicted protein |
| LMOG_00891_at | predicted protein |
| LMOG_00894_s_at | predicted protein |
| LMOG_00906_s_at | predicted protein |
| LMOG_01016_s_at | predicted protein |
| LMOG_01050_at | predicted protein |
| LMOG_01088_s_at | predicted protein |
| LMOG_01125_at | predicted protein |
| LMOG_01518_at | predicted protein |
| LMOG_01576_at | predicted protein |
| LMOG_01581_s_at | predicted protein |
| LMOG_01752_s_at | predicted protein |
| LMOG_02003_at | predicted protein |
| LMOG_02125_s_at | predicted protein |
| LMOG_02478_x_at | conserved hypothetical protein/Pfam=PF06458.4 |
| LMOG_02729_s_at | predicted protein |
| LMOG_03190_s_at | conserved hypothetical protein |
| LMPG_00294_s_at | predicted protein |
| LMPG_01354_s_at | predicted protein |
| LMPG_01354_x_at | predicted protein |
| LMRG_00094_s_at | glycosyl hydrolase/Pfam=PF07748.5 |
| LMRG_00214_x_at | conserved hypothetical protein/Pfam=PF08241.4 |
| LMRG_00305_x_at | conserved hypothetical protein |
| LMRG_00343_x_at | conserved hypothetical protein/Pfam=PF04238.4 |
| LMRG_00358_x_at | conserved hypothetical protein |
| LMRG_00370_x_at | flagellar basal body rod protein FlgG/Pfam=PF00460.12 |
| LMRG_00505_s_at | molybdenum cofactor biosynthesis protein E/Pfam=PF02391.9 |
| LMRG_00567_s_at | conserved hypothetical protein |
| LMRG_00810_s_at | folD/Pfam=PF02882.11 |
| LMRG_01072_s_at | histidinolphosphate aminotransferase/Pfam=PF00155.13 |
| LMRG_01101_s_at | phosphopentomutase/Pfam=PF08342.2 |
| LMRG_01364_s_at | aminopeptidase protein |
| LMRG_01408_s_at | threonyltRNA synthetase/Pfam=PF07973.6 |
| LMRG_01662_x_at | conserved hypothetical protein/Pfam=PF03060.7 |
| LMRG_01799_s_at | rnr/Pfam=PF08206.3 |
| LMRG_01805_x_at | predicted protein |
| LMRG_01894_x_at | conserved hypothetical protein |
| LMRG_01898_s_at | BC component PTS system mannitolpermease II/Pfam=PF02378.10 |
| LMRG_01904_x_at | conserved hypothetical protein |
| LMRG_01943_x_at | conserved hypothetical protein |
| LMRG_02099_s_at | conserved hypothetical protein |
| LMRG_02214_s_at | conserved hypothetical protein/Pfam=PF06541.3 |
| LMRG_02266_x_at | conserved hypothetical protein/Pfam=PF06961.5 |
| LMRG_02605_x_at | glycosyl hydrolase/Pfam=PF00232.10 |
| LMRG_02889_s_at | conserved hypothetical protein/Pfam=PF02775.13 |
| LMRG_02890_s_at | ATP synthase F1 |
| LMRG_02929_s_at | glucose6phosphate 1dehydrogenase/Pfam=PF02781.8 |
| LMRG_02962_x_at | predicted protein |

NK: Gene function not known as predicted by Gene Locator and Interpolated Markov ModelER 3 (Glimmer3)
