# Supplementary material for: High Density Microarray Analysis Reveals New Insights into Genetic Footprints of Listeria monocytogenes Strains Involved in Listeriosis Outbreaks
Source: PLoS One. 2012 Mar 21;7(3):e32896. doi: 10.1371/journal.pone.0032896 (PMC3310058; doi:10.1371/journal.pone.0032896)
Supplement: Table S3 — Probe-sets uniquely present in the serotype 1/2b strains. (DOCX) [file pone.0032896.s003.docx]

**Supporting Information Table S3: Probe-sets uniquely present in the serotype 1/2b strains**

| Probe ID | Annotation |
| --- | --- |
| AARO_1290_s_at | NK |
| AARO_1767_s_at | NK |
| IGLMHCC_0123_at | Intergenic region |
| LMBG_00296_s_at | predicted protein |
| LMBG_00297_s_at | glycosyl transferase/Pfam=PF00535.18 |
| LMBG_01400_at | conserved hypothetical protein |
| LMBG_01400_x_at | conserved hypothetical protein |
| LMBG_01454_s_at | predicted protein |
| LMBG_01456_s_at | conserved hypothetical protein |
| LMBG_01837_s_at | conserved hypothetical protein/Pfam=PF03706.5 |
| LMBG_02275_s_at | internalin B/Pfam=PF09479.2 |
| LMBG_02473_at | conserved hypothetical protein |
| LMFG_00178_x_at | cytidinedeoxycytidylate deaminase/Pfam=PF00383.14 |
| LMFG_03141_s_at | transcription termination factor Rho/Pfam=PF07498.4 |
| LMHCC_0037_s_at | pyrG CTP synthase/GI=217332607 |
| LMHCC_0425_s_at | maltose phosphorylase/GI=217332988 |
| LMHCC_2815_x_at | tRNA-specific adenosine deaminase/GI=217335353 |
| LMHG_00425_x_at | cytidinedeoxycytidylate deaminase/Pfam=PF00383.14 |

NK: Gene function not known as predicted by Gene Locator and Interpolated Markov ModelER 3 (Glimmer3)
