# Supplementary material for: High Density Microarray Analysis Reveals New Insights into Genetic Footprints of Listeria monocytogenes Strains Involved in Listeriosis Outbreaks
Source: PLoS One. 2012 Mar 21;7(3):e32896. doi: 10.1371/journal.pone.0032896 (PMC3310058; doi:10.1371/journal.pone.0032896)
Supplement: Table S4 — Probe-sets uniquely present in the serotype 4b strains. (DOCX) [file pone.0032896.s004.docx]

**Supporting Information Table S4: Probe-sets uniquely present in the serotype 4b strains**

| **Probe ID** | **Annotation** |
| --- | --- |
| AARK_0127_at | NK |
| AARL_0147_s_at | NK |
| AARL_0368_s_at | NK |
| AARL_0603_s_at | NK |
| AARL_0763_s_at | NK |
| IGLm4b_00013_at | Intergenic region |
| IGLm4b_00014_s_at | Intergenic region |
| IGLm4b_01084_x_at | Intergenic region |
| IGLm4b_02144_s_at | Intergenic region |
| IGLm4b_02145_s_at | Intergenic region |
| IGLMHCC_0135_at | Intergenic region |
| IGLMHCC_2527_x_at | Intergenic region |
| IGLMHCC_2528_x_at | Intergenic region |
| IGLMOf2365_0014_s_at | Intergenic region |
| IGLMOf2365_0016_at | Intergenic region |
| IGLMOf2365_0118_at | Intergenic region |
| IGLMOf2365_0119_s_at | Intergenic region |
| IGLMOf2365_0340_at | Intergenic region |
| IGLMOf2365_0449_s_at | Intergenic region |
| IGLMOf2365_0450_at | Intergenic region |
| IGLMOf2365_0470_s_at | Intergenic region |
| IGLMOf2365_0527_s_at | Intergenic region |
| IGLMOf2365_1091_s_at | Intergenic region |
| IGLMOf2365_1093_s_at | Intergenic region |
| IGLMOf2365_1094_at | Intergenic region |
| IGLMOf2365_1095_at | Intergenic region |
| IGLMOf2365_1099_at | Intergenic region |
| IGLMOf2365_1100_at | Intergenic region |
| IGLMOf2365_1102_s_at | Intergenic region |
| IGLMOf2365_1105_s_at | Intergenic region |
| IGLMOf2365_1106_at | Intergenic region |
| IGLMOf2365_1106_x_at | Intergenic region |
| IGLMOf2365_1900_s_at | Intergenic region |
| IGLMOf2365_1901_at | Intergenic region |
| IGLMOf2365_2524_s_at | Intergenic region |
| IGLMOf2365_2525_at | Intergenic region |
| IGLMOf2365_2525_x_at | Intergenic region |
| IGLMOf2365_2680_at | Intergenic region |
| IGLMOf2365_2680_x_at | Intergenic region |
| IGLMOf2365_2740_at | Intergenic region |
| IGLMOf2365_2742_at | Intergenic region |
| IGLMOf2365_2877_at | Intergenic region |
| IGLMOf2365_2877_x_at | Intergenic region |
| Lm4b_00013_s_at | Hypothetical protein of unknown function/GI=225875114 |
| Lm4b_00015_s_at | Putative autolysin (amidase) |
| Lm4b_00462_s_at | inlB Internalin B/GI=225875528 |
| Lm4b_02144_s_at | Putative protein possibly involved in teichoic acid biosynthesis/GI=225877189 |
| LMHCC_1544_s_at | cdp-glycerol:poly(glycerophosphate) glycerophosphotransFerase (polyglycerol phosphate polymerase) (cgptase) (majorteichoic acid biosynthesis protein f)/GI=217334094 |
| LMHG_03285_s_at | teichoic acid biosynthesis domaincontaining protein/Pfam=PF04464.6 |
| LMHG_03286_s_at | conserved hypothetical protein |
| LMOf2365_0014_at | hypothetical protein/GI=46879502 |
| LMOf2365_0118_s_at | conserved hypothetical protein/GI=46879604 |
| LMOf2365_0119_s_at | transcriptional regulator, ArsR family/GI=46879605 |
| LMOf2365_0145_s_at | conserved hypothetical protein/GI=46879631 |
| LMOf2365_0340_s_at | conserved hypothetical protein/GI=46879825 |
| LMOf2365_0341_s_at | endonucleaseexonucleasephosphatase family protein/GI=46879826 |
| LMOf2365_0449_s_at | conserved hypothetical protein/GI=46879932 |
| LMOf2365_0458_s_at | hypothetical protein/GI=46879941 |
| LMOf2365_0469_s_at | hypothetical protein/GI=46879952 |
| LMOf2365_0954_s_at | dolichyl-phosphate-mannose-protein mannosyltransferase family protein/GI=46880434 |
| LMOf2365_1085_s_at | conserved hypothetical protein/GI=46880563 |
| LMOf2365_1091_s_at | techoic acid ABC transporter, permease protein/GI=46880569 |
| LMOf2365_1092_at | putative techoic acid ABC transporter, ATP-binding protein/GI=46880570 |
| LMOf2365_1093_s_at | N-acetylmuramoyl-L-alanine amidase, family 4/GI=46880571 |
| LMOf2365_1094_s_at | conserved hypothetical protein/GI=46880572 |
| LMOf2365_1095_at | glycosyl transferase, group 2 family protein/GI=46880573 |
| LMOf2365_1096_s_at | putative membrane protein/GI=46880574 |
| LMOf2365_1097_s_at | glycosyl transferase, group 2 family protein/GI=46880575 |
| LMOf2365_1098_s_at | conserved hypothetical protein/GI=46880576 |
| LMOf2365_1099_at | galU UTP-glucose-1-phosphate uridylyltransferase/GI=46880577 |
| LMOf2365_1100_at | nucleotidyltransferase family protein/GI=46880578 |
| LMOf2365_1100_s_at | nucleotidyltransferase family protein/GI=46880578 |
| LMOf2365_1101_s_at | alcohol dehydrogenase, zinc-dependent/GI=46880579 |
| LMOf2365_1102_s_at | glycosyl transferase, group 2 family protein/GI=46880580 |
| LMOf2365_1105_at | tagD glycerol-3-phosphate cytidylyltransferase/GI=46880583 |
| LMOf2365_1105_s_at | tagD glycerol-3-phosphate cytidylyltransferase/GI=46880583 |
| LMOf2365_1198_s_at | transcriptional regulator, AraC family/GI=46880676 |
| LMOf2365_1900_s_at | putative serine protease/GI=46881373 |
| LMOf2365_2155_s_at | glycosyl transferase, family 65/GI=46881628 |
| LMOf2365_2522_s_at | gtcA cell wall teichoic acid glycosylation protein GtcA/GI=46881993 |
| LMOf2365_2523_s_at | rho transcription termination factor Rho/GI=46881994 |
| LMOf2365_2525_s_at | putative membrane protein/GI=46881996 |
| LMOf2365_2530_s_at | N-acetylmuramoyl-L-alanine amidase, family 2/GI=46882001 |
| LMOf2365_2679_s_at | MutTnudix family protein/GI=46882151 |
| LMOf2365_2740_at | putative membrane protein/GI=46882212 |
| LMOf2365_2741_at | glycosyl transferase, group 2 family protein/GI=46882213 |
| LMOh7858_0478_at | conserved hypothetical protein/GI=47019139 |
| LMOh7858_0489_s_at | hypothetical protein/GI=47019146 |
| LMOh7858_0490_s_at | hypothetical protein/GI=47019140 |
| LMOh7858_0496_s_at | hypothetical protein/GI=47019143 |
| LMOh7858_1140_s_at | techoic acid ABC transporter, efflux permease/GI=47017032 |
| LMOh7858_1142_s_at | autolysin, putative/GI=47017034 |
| LMSG_03017_s_at | conserved hypothetical protein |

NK: Gene function not known as predicted by Gene Locator and Interpolated Markov ModelER 3 (Glimmer3)
