# Supplementary material for: High Density Microarray Analysis Reveals New Insights into Genetic Footprints of Listeria monocytogenes Strains Involved in Listeriosis Outbreaks
Source: PLoS One. 2012 Mar 21;7(3):e32896. doi: 10.1371/journal.pone.0032896 (PMC3310058; doi:10.1371/journal.pone.0032896)
Supplement: Table S5 — Probe-sets uniquely present in the serotype 4b, epidemic clone I. (DOCX) [file pone.0032896.s005.docx]

**Supporting Information Table S5: Probe-sets uniquely present in the serotype 4b, epidemic clone I**

| **Probe ID** | **Annotation** |
| --- | --- |
| AARI_0552_s_at | 100% similar to LMHCC_2830 |
| AARL_0232_s_at | NK |
| AARL_0233_s_at | 98% similar to LMOf2365_0325 |
| AARM_0860_x_at | NK |
| IGLMOf2365_0316_at | Intergenic region |
| IGLMOf2365_0325_at | Intergenic region |
| IGLMOf2365_0326_at | Intergenic region |
| IGLMOf2365_0328_at | Intergenic region |
| IGLMOf2365_0329_at | Intergenic region |
| IGLMOf2365_0549_at | Intergenic region |
| IGLMOf2365_0549_x_at | Intergenic region |
| IGLMOf2365_0687_at | Intergenic region |
| IGLMOf2365_0688_at | Intergenic region |
| IGLMOf2365_1139_at | Intergenic region |
| IGLMOf2365_2051_at | Intergenic region |
| IGLMOf2365_2095_x_at | Intergenic region |
| IGLMOf2365_2341_at | Intergenic region |
| IGLMOf2365_2341_x_at | Intergenic region |
| IGLMOf2365_2346_at | Intergenic region |
| IGLMOf2365_2347_at | Intergenic region |
| IGLMOf2365_2348_at | Intergenic region |
| IGLMOf2365_2511_at | Intergenic region |
| IGLMOf2365_2511_x_at | Intergenic region |
| IGLMOf2365_2701_at | Intergenic region |
| IGLMOf2365_2704_at | Intergenic region |
| IGLMOf2365_2705_at | Intergenic region |
| IGLMOf2365_2706_at | Intergenic region |
| IGLMOf2365_2707_at | Intergenic region |
| IGLMOf2365_2708_at | Intergenic region |
| IGLMOf2365_2750_at | Intergenic region |
| IGLMOf2365_2750_x_at | Intergenic region |
| IGLMOf2365_2797_at | Intergenic region |
| IGLMOf2365_2797_x_at | Intergenic region |
| IGLMOf2365_2798_at | Intergenic region |
| IGLMOf2365_2799_at | Intergenic region |
| IGLMOf2365_2801_at | Intergenic region |
| IGLMOf2365_2879_x_at | Intergenic region |
| LMFG_00474_x_at | thymidylate synthase/Pfam=PF00303.11 |
| LMHCC_2165_x_at | cell wall surface anchor family protein/GI=217334709 |
| LMHCC_2717_s_at | conserved hypothetical protein/GI=217335258 |
| LMHCC_2821_at | hypothetical protein/GI=217335359 |
| LMIG_02842_x_at | cell wall surface anchor family protein/Pfam=PF00746.13 |
| LMOf2365_0084_at | hypothetical protein/GI=46879570 |
| LMOf2365_0323_at | hypothetical protein/GI=46879809 |
| LMOf2365_0323_x_at | hypothetical protein/GI=46879809 |
| LMOf2365_0325_at | putative type II restriction enzyme Sau3AI/GI=46879810 |
| LMOf2365_0326_at | DNA-binding protein/GI=46879811 |
| LMOf2365_0326_s_at | DNA-binding protein/GI=46879811 |
| LMOf2365_0327_at | C-5 cytosine-specific DNA methylase family protein/GI=46879812 |
| LMOf2365_0327_s_at | C-5 cytosine-specific DNA methylase family protein/GI=46879812 |
| LMOf2365_0328_s_at | conserved domain protein/GI=46879813 |
| LMOf2365_0346_x_at | conserved hypothetical protein/GI=46879831 |
| LMOf2365_0383_at | conserved hypothetical protein/GI=46879867 |
| LMOf2365_0498_x_at | cell wall surface anchor family protein/GI=46879980 |
| LMOf2365_0505_at | conserved domain protein/GI=46879987 |
| LMOf2365_0505_x_at | conserved domain protein/GI=46879987 |
| LMOf2365_0506_s_at | conserved hypothetical protein/GI=46879988 |
| LMOf2365_0557_x_at | conserved hypothetical protein/GI=46880038 |
| LMOf2365_0687_at | conserved hypothetical protein/GI=46880168 |
| LMOf2365_1275_at | hydrolase, alphabeta fold family/GI=46880752 |
| LMOf2365_1752_at | conserved hypothetical protein/GI=46881228 |
| LMOf2365_2345_at | aspartate kinase family protein/GI=46881817 |
| LMOf2365_2346_s_at | conserved hypothetical protein/GI=46881818 |
| LMOf2365_2347_at | conserved domain protein/GI=46881819 |
| LMOf2365_2347_s_at | conserved domain protein/GI=46881819 |
| LMOf2365_2348_at | hypothetical protein/GI=46881820 |
| LMOf2365_2406_at | conserved hypothetical protein/GI=46881878 |
| LMOf2365_2684_at | dnaX DNA polymerase III, gamma and tau subunits/GI=46882156 |
| LMOf2365_2688_at | putative PTS system, cellobiose-specific, IIC component/GI=46882160 |
| LMOf2365_2693_s_at | conserved hypothetical protein/GI=46882165 |
| LMOf2365_2701_at | conserved hypothetical protein/GI=46882173 |
| LMOf2365_2702_at | hypothetical protein/GI=46882174 |
| LMOf2365_2703_at | conserved domain protein/GI=46882175 |
| LMOf2365_2704_at | hypothetical protein/GI=46882176 |
| LMOf2365_2705_at | hypothetical protein/GI=46882177 |
| LMOf2365_2706_at | hypothetical protein/GI=46882178 |
| LMOf2365_2707_at | putative membrane protein/GI=46882179 |
| LMOf2365_2785_at | PTS system, IIA component/GI=46882257 |
| LMOf2365_2785_x_at | PTS system, IIA component/GI=46882257 |
| LMOf2365_2797_at | conserved hypothetical protein/GI=46882269 |
| LMOf2365_2797_x_at | conserved hypothetical protein/GI=46882269 |
| LMOf2365_2798_at | hypothetical protein/GI=46882270 |
| LMOf2365_2799_at | DNA-binding protein/GI=46882271 |
| LMOf2365_2801_at | gidA glucose-inhibited division protein A/GI=46882272 |
| LMOf2365_2801_x_at | gidA glucose-inhibited division protein A/GI=46882272 |

NK: Gene function not known as predicted by Gene Locator and Interpolated Markov ModelER 3 (Glimmer3)
