# Supplementary material for: High Density Microarray Analysis Reveals New Insights into Genetic Footprints of Listeria monocytogenes Strains Involved in Listeriosis Outbreaks
Source: PLoS One. 2012 Mar 21;7(3):e32896. doi: 10.1371/journal.pone.0032896 (PMC3310058; doi:10.1371/journal.pone.0032896)
Supplement: Table S6 — Probe-sets uniquely present in the serotype 4b, epidemic clone II. (DOCX) [file pone.0032896.s006.docx]

**Supporting Information Table S6: Probe-sets uniquely present in the serotype 4b, epidemic clone II**

| **Probe ID** | **Annotation** |
| --- | --- |
| AARI_0397_s_at | 99% similar to lmo0767 |
| AARK_1189_x_at | NK |
| AARK_1677_at | 99% similar to LMHCC_2212 |
| AARK_1706_s_at | 98% similar to lmo0767 |
| AARL_0455_s_at | NK |
| AARM_1004_s_at | 99% similar to lmo0765 |
| AARM_1327_x_at | NK |
| AARO_1464_s_at | 98% similar to lmo0770 |
| AARO_1842_s_at | 99% similar to lmo0307 |
| AARY_0457_s_at | 100% similar to lmo0310 |
| AARY_0460_s_at | 99% similar to lmo0313 |
| IGLm4b_00397_at | Intergenic region |
| IGLm4b_00397_x_at | Intergenic region |
| IGLm4b_00398a_x_at | Intergenic region |
| IGLm4b_00400_x_at | Intergenic region |
| IGLm4b_02814_at | Intergenic region |
| IGLMHCC_0840_at | Intergenic region |
| IGLMHCC_0840_x_at | Intergenic region |
| IGLMHCC_1221_at | Intergenic region |
| IGLMHCC_1818_at | Intergenic region |
| IGLMHCC_2255_at | Intergenic region |
| IGLMHCC_2325_at | Intergenic region |
| IGLMHCC_2326_at | Intergenic region |
| IGLMHCC_T001_s_at | Intergenic region |
| IGlmo0309_at | Intergenic region |
| IGlmo0310_at | Intergenic region |
| IGlmo0311_at | Intergenic region |
| IGlmo0314_at | Intergenic region |
| IGlmo0381_s_at | Intergenic region |
| IGlmo0382_s_at | Intergenic region |
| IGlmo0765_s_at | Intergenic region |
| IGlmo0771_s_at | Intergenic region |
| IGlmo0772_at | Intergenic region |
| IGlmo0815_at | Intergenic region |
| IGlmo2595_at | Intergenic region |
| IGlmo2596_at | Intergenic region |
| IGlmo2596_x_at | Intergenic region |
| IGlmo2932_x_at | Intergenic region |
| Lm4b_00398a_s_at | Hypothetical protein of unknown function |
| Lm4b_00398b_s_at | NK |
| Lm4b_00784_s_at | Putative sugar ABC transporter (binding protein)/GI=225875848 |
| Lm4b_00785_s_at | Putative alpha-1,6-mannanase/GI=225875849 |
| Lm4b_01653_s_at | Putative sigma factor regulator/GI=225876705 |
| Lm4b_01654_x_at | Hypothetical protein of unknown function/GI=225876706 |
| Lm4b_02804_at | Putative phosphoglucomutase/GI=225877843 |
| LMBG_00809_x_at | conserved hypothetical protein |
| LMFG_02973_s_at | phage protein |
| LMHCC_0188_x_at | sufD FeS assembly protein SufD/GI=217332756 |
| LMHCC_1817_s_at | transcriptional regulator/GI=217334364 |
| LMHCC_2009_x_at | domain of unknown function, putative/GI=217334554 |
| LMHCC_2255_s_at | conserved hypothetical protein/GI=217334799 |
| LMHCC_2701_s_at | amino-domain protein/GI=217335242 |
| LMHG_01146_x_at | InlC2/Pfam=PF09479.2 |
| LMHG_01357_x_at | conserved hypothetical protein/Pfam=PF01458.9 |
| LMHG_03118_s_at | InlG/Pfam=PF09479.2 |
| LMIG_02175_s_at | conserved hypothetical protein |
| LMKG_02912_at | predicted protein |
| LMKG_02912_x_at | predicted protein |
| LMKG_02914_at | predicted protein |
| LMKG_02915_at | conserved hypothetical protein |
| LMKG_02915_s_at | conserved hypothetical protein |
| lmo0309_s_at | GI=16409673 |
| lmo0311_s_at | GI=16409675 |
| lmo0312_s_at | GI=16409676 |
| lmo0766_s_at | GI=16410155 |
| lmo0771_s_at | GI=16410160 |
| lmo0815_s_at | GI=16410204 |
| lmo0816_s_at | GI=16410205 |
| lmo2594_s_at | GI=16412082 |
| LMOf6854_0275_x_at | inlC2 internalin C2/GI=47015114 |
| LMOh7858_0095_at | hypothetical protein/GI=47019410 |
| LMOh7858_0096_at | conserved hypothetical protein/GI=47019411 |
| LMOh7858_0096_x_at | conserved hypothetical protein/GI=47019411 |
| LMOh7858_0289_x_at | conserved hypothetical protein/GI=47020071 |
| LMOh7858_0291_at | Leucine Rich Repeat domain protein/GI=47020073 |
| LMOh7858_0293_at | inlD internalin D/GI=47020075 |
| LMOh7858_0295_at | inlE internalin E/GI=47020077 |
| LMOh7858_0339_s_at | conserved hypothetical protein/GI=47020119 |
| LMOh7858_0339_x_at | conserved hypothetical protein/GI=47020119 |
| LMOh7858_0341_s_at | conserved hypothetical protein/GI=47020121 |
| LMOh7858_0342_at | hypothetical protein/GI=47020238 |
| LMOh7858_0345_s_at | conserved hypothetical protein/GI=47020239 |
| LMOh7858_0417_s_at | conserved hypothetical protein/GI=47020192 |
| LMOh7858_0465_s_at | conserved hypothetical protein/GI=47019072 |
| LMOh7858_0532_at | HD domain protein/GI=47019128 |
| LMOh7858_0532_x_at | HD domain protein/GI=47019128 |
| LMOh7858_0533_at | conserved domain protein/GI=47019129 |
| LMOh7858_0533_x_at | conserved domain protein/GI=47019129 |
| LMOh7858_0718_at | endonuclease III domain protein/GI=47019936 |
| LMOh7858_0730_at | ABC transporter, ATP-binding protein/GI=47019948 |
| LMOh7858_0763_x_at | flagellar motor switch protein, putative/GI=47019979 |
| LMOh7858_0863_at | oxidoreductase, short chain dehydrogenasereductase family/GI=47017692 |
| LMOh7858_0864_at | merR transcriptional regulator, MerR family/GI=47017693 |
| LMOh7858_0864_x_at | merR transcriptional regulator, MerR family/GI=47017693 |
| LMOh7858_0865_at | conserved hypothetical protein/GI=47017694 |
| LMOh7858_0866_at | transcription regulator/GI=47017695 |
| LMOh7858_0867_at | RNA-directed DNA polymerase from retron ec67/GI=47017696 |
| LMOh7858_0867_s_at | RNA-directed DNA polymerase from retron ec67/GI=47017696 |
| LMOh7858_1161_at | Helix-turn-helix domain protein/GI=47019609 |
| LMOh7858_1162_at | transcriptional regulator, putative/GI=47019610 |
| LMOh7858_1163_at | site-specific recombinase, phage integrase family, putative/GI=47019611 |
| LMOh7858_1164_at | nikA protein, putative/GI=47019612 |
| LMOh7858_1165_at | hypothetical protein/GI=47019744 |
| LMOh7858_1166_at | conserved hypothetical protein/GI=47019613 |
| LMOh7858_1167_at | Rlx-like protein/GI=47019745 |
| LMOh7858_1168_at | AAA superfamily ATPase/GI=47019614 |
| LMOh7858_1169_at | hypothetical protein/GI=47019615 |
| LMOh7858_1170_at | putative immunity repressor protein/GI=47019616 |
| LMOh7858_1171_at | hypothetical protein/GI=47019617 |
| LMOh7858_1172_at | hypothetical protein/GI=47019750 |
| LMOh7858_1173_at | hypothetical protein/GI=47019618 |
| LMOh7858_1174_at | conserved hypothetical protein/GI=47019619 |
| LMOh7858_1200_at | conserved hypothetical protein/GI=47019644 |
| LMOh7858_1494_x_at | conserved hypothetical protein TIGR00282/GI=47018234 |
| LMOh7858_1758_s_at | STAS domain protein/GI=47017939 |
| LMOh7858_1759_x_at | conserved hypothetical protein/GI=47017940 |
| LMOh7858_1994_s_at | conserved hypothetical protein/GI=47018383 |
| LMOh7858_2150_s_at | cell wall surface anchor family protein/GI=47018448 |
| LMOh7858_2410_at | conserved hypothetical protein/GI=47019241 |
| LMOh7858_2411_at | conserved hypothetical protein/GI=47019242 |
| LMOh7858_2411_x_at | conserved hypothetical protein/GI=47019242 |
| LMOh7858_2444_at | SAM:benzoic acid carboxyl methyltransferase/GI=47019185 |
| LMOh7858_2444_x_at | SAM:benzoic acid carboxyl methyltransferase/GI=47019185 |
| LMOh7858_2445_at | phage conserved hypothetical protein TIGR01671/GI=47019186 |
| LMOh7858_2446_at | hypothetical protein/GI=47019243 |
| LMOh7858_2447_at | protein gp51/GI=47019187 |
| LMOh7858_2448_at | Gp32 protein/GI=47019188 |
| LMOh7858_2448_x_at | Gp32 protein/GI=47019188 |
| LMOh7858_2473_at | lipoprotein, putative/GI=47019206 |
| LMOh7858_2753_at | hypothetical protein/GI=47018085 |
| LMOh7858_2754_at | ulcer associated adenine specific DNA methyltransferase, putative/GI=47018086 |
| LMOh7858_2755_at | conserved hypothetical protein/GI=47018105 |
| LMOh7858_2756_at | DNA repair protein RadC/GI=47018087 |
| LMOh7858_2757_at | hypothetical protein/GI=47018088 |
| LMOh7858_2758_at | hypothetical protein/GI=47018089 |
| LMOh7858_2759_at | hypothetical protein/GI=47018090 |
| LMOh7858_2760_at | hypothetical protein/GI=47018091 |
| LMOh7858_2761_at | hypothetical protein/GI=47018092 |
| LMOh7858_2762_at | hypothetical protein/GI=47018093 |
| LMOh7858_2763_at | hypothetical protein/GI=47018094 |
| LMOh7858_2764_at | site-specific recombinase, phage integrase family, putative/GI=47018095 |
| LMOh7858_2903_at | serinethreonine protein phosphatase family protein/GI=47017808 |
| LMOh7858_2958_at | dihydroxyacetone kinase family protein/GI=47019295 |
| LMOh7858_2962_at | MutTnudix family protein/GI=47019299 |
| LMOh7858_3069_s_at | conserved hypothetical protein/GI=47018361 |
| LMOh7858_3070_s_at | conserved hypothetical protein/GI=47018340 |
| LMOh7858_3095_at | glycosyl transferase, family 65/GI=47018398 |
| LMRG_01324_x_at | conserved hypothetical protein |
| LMRG_01773_s_at | phosphoglucomutasephosphomannomutase family protein/Pfam=PF02880.8 |

NK: Gene function not known as predicted by Gene Locator and Interpolated Markov ModelER 3 (Glimmer3)
