# Supplementary material for: High Density Microarray Analysis Reveals New Insights into Genetic Footprints of Listeria monocytogenes Strains Involved in Listeriosis Outbreaks
Source: PLoS One. 2012 Mar 21;7(3):e32896. doi: 10.1371/journal.pone.0032896 (PMC3310058; doi:10.1371/journal.pone.0032896)
Supplement: Table S7 — Probe-sets uniquely present in the serotype 4b, epidemic clone IV. (DOCX) [file pone.0032896.s007.docx]

**Supporting Information Table S7: Probe-sets uniquely present in the serotype 4b, epidemic clone IV**

| **Probe ID** | **Annotation** |
| --- | --- |
| AARI_0046_s_at | NK |
| AARI_0381_s_at | NK |
| AARK_0852_x_at | NK |
| AARK_1188_at | 99% similar to LMOf2365_0508 |
| AARM_0108_at | NK |
| AARM_0110_at | NK |
| AARM_0161_s_at | 100% similar to lmo2302 |
| AARM_0162_at | NK |
| AARO_1643_x_at | NK |
| IGLm4b_00497_at | Intergenic region |
| IGLm4b_00497_x_at | Intergenic region |
| IGLm4b_00499_s_at | Intergenic region |
| IGLm4b_00499_x_at | Intergenic region |
| IGLMHCC_1379_at | Intergenic region |
| IGLMHCC_2529_at | Intergenic region |
| IGLMHCC_2529_x_at | Intergenic region |
| IGlmo2206_x_at | Intergenic region |
| IGlmo2302_x_at | Intergenic region |
| IGlmo2806_x_at | Intergenic region |
| Lm4b_00370_at | Putative regulatory protein (DeoR family)/GI=225875440 |
| Lm4b_00497_at | Conserved hypothetical proteins/GI=225875562 |
| Lm4b_00497_s_at | Conserved hypothetical proteins/GI=225875562 |
| Lm4b_00498_at | Putative secreted protein/GI=225875563 |
| Lm4b_02375_s_at | GI=225877416 |
| LMBG_00762_s_at | predicted protein |
| LMBG_00894_x_at | primosomal protein DnaI/Pfam=PF07319.3 |
| LMBG_01657_at | conserved hypothetical protein |
| LMBG_02369_x_at | inorganic pyrophosphatase |
| LMBG_02370_x_at | conserved hypothetical protein |
| LMBG_02371_s_at | predicted protein |
| LMBG_02373_s_at | replicationassociated protein RepB |
| LMBG_02374_s_at | conserved hypothetical protein |
| LMBG_02375_at | oxidoreductase |
| LMBG_02376_at | predicted protein/Pfam=PF00440.15 |
| LMBG_02376_s_at | predicted protein/Pfam=PF00440.15 |
| LMBG_02377_at | predicted protein |
| LMBG_02378_at | predicted protein |
| LMBG_02378_s_at | predicted protein |
| LMBG_02553_x_at | conserved hypothetical protein/Pfam=PF05913.3 |
| LMBG_02937_s_at | thioesterase/Pfam=PF03061.14 |
| LMFG_01112_x_at | conserved hypothetical protein/Pfam=PF00923.11 |
| LMFG_01220_x_at | conserved hypothetical protein/Pfam=PF04794.4 |
| LMFG_01521_x_at | conserved hypothetical protein |
| LMFG_01791_at | conserved hypothetical protein |
| LMFG_01791_x_at | conserved hypothetical protein |
| LMFG_03051_x_at | inorganic pyrophosphatase |
| LMFG_03054_s_at | predicted protein |
| LMFG_03054_x_at | predicted protein |
| LMHCC_1392_s_at | Gp37 protein/GI=217333943 |
| LMHCC_2057_s_at | glycosyl hydrolase, family 1/GI=217334602 |
| LMHCC_2294_s_at | inorganic pyrophosphatase/GI=217334838 |
| LMHCC_2294_x_at | inorganic pyrophosphatase/GI=217334838 |
| LMHCC_2393_x_at | conserved hypothetical protein/GI=217334936 |
| LMHCC_2968_at | putative gp69/GI=217335505 |
| LMHCC_2968_x_at | putative gp69/GI=217335505 |
| LMHG_01965_x_at | phosphosugarbinding transcriptional regulator/Pfam=PF01418.9 |
| LMHG_02312_x_at | primosomal protein DnaI/Pfam=PF07319.3 |
| LMLG_00743_s_at | predicted protein |
| LMLG_00745_s_at | conserved hypothetical protein |
| LMLG_00746_s_at | conserved hypothetical protein/Pfam=PF04471.4 |
| lmo0479_s_at | putative secreted protein/GI=16409855 |
| lmo2302_s_at | GI=16411772 |
| lmo2490_x_at | GI=16411978 |
| LMOf6854_0646_s_at | conserved hypothetical protein/GI=47013873 |
| LMOf6854_2316_s_at | amino acid ABC transporter, permease protein, HisGluGlnArgopine family/GI=47013921 |
| LMRG_02931_at | predicted protein |
| LMSG_00002_at | conserved hypothetical protein/Pfam=PF02086.7 |
| LMSG_00108_at | cell division protein/Pfam=PF01098.11 |
| LMSG_00109_at | ftsW/Pfam=PF01098.11 |
| LMSG_00110_at | magnesiumtranslocating Ptype ATPase/Pfam=PF00702.18 |
| LMSG_00178_at | hydrolase/Pfam=PF08530.2 |
| LMSG_00232_x_at | tRNA uridine 5carboxymethylaminomethyl modification enzyme GidA/Pfam=PF01134.14 |
| LMSG_00485_s_at | conserved hypothetical protein |
| LMSG_00888_x_at | primosomal protein DnaI/Pfam=PF07319.3 |
| LMSG_01239_at | DNA helicase |
| LMSG_01240_at | yeeC |
| LMSG_01573_at | type I restrictionmodification system/Pfam=PF02384.8 |
| LMSG_01574_at | HsdS/Pfam=PF01420.11 |
| LMSG_01577_at | conserved hypothetical protein |
| LMSG_01582_x_at | conserved hypothetical protein/Pfam=PF04794.4 |
| LMSG_01584_x_at | hydroxyethylthiazole kinase/Pfam=PF02110.7 |
| LMSG_01620_at | conserved hypothetical protein |
| LMSG_01702_x_at | conserved hypothetical protein |
| LMSG_02105_at | spermidine N1acetyltransferase/Pfam=PF00583.16 |
| LMSG_02188_x_at | conserved hypothetical protein |
| LMSG_02455_x_at | prophage LambdaLm01/Pfam=PF01520.10 |
| LMSG_02462_x_at | oligopeptide ABC transporter/Pfam=PF00528.14 |
| LMSG_02556_x_at | conserved hypothetical protein |
| LMSG_02557_at | conserved hypothetical protein/Pfam=PF06860.3 |
| LMSG_02558_at | conserved hypothetical protein |
| LMSG_02665_at | class I glutamine amidotransferase/Pfam=PF00117.20 |
| LMSG_02670_at | peptidase U32 |
| LMSG_02671_at | solutebinding family 5 protein |
| LMSG_02785_at | phage protein |
| LMSG_02941_s_at | TerL |
| LMSG_02956_x_at | phage protein |
| LMSG_02979_at | phage protein |
| LMSG_02988_at | DNAcytosine methyltransferase/Pfam=PF00145.9 |
| LMSG_02992_at | conserved hypothetical protein |
| LMSG_02992_x_at | conserved hypothetical protein |
| LMSG_02994_at | phage protein/Pfam=PF07852.3 |
| LMSG_02998_at | transposase/Pfam=PF01609.13 |
| LMSG_03005_x_at | major tail shaft protein |
| LMSG_03012_at | conserved hypothetical protein |
| LMSG_03069_at | type I sitespecific deoxyribonuclease/Pfam=PF04313.6 |
| LMSG_03070_at | type I sitespecific deoxyribonuclease/Pfam=PF04851.7 |
| LMSG_03071_s_at | restriction endonuclease |
| LMSG_03074_at | thiaminephosphate pyrophosphorylase/Pfam=PF02581.9 |
| LMSG_03075_at | LcmR protein/Pfam=PF01381.14 |
| LMSG_03076_at | cytosine methyl transferase/Pfam=PF00145.9 |
| LMSG_03077_at | conserved hypothetical protein |
| LMSG_03097_at | yeeA |
| LMSG_03108_at | replicationassociated protein RepB |
| LMSG_03115_at | predicted protein |
| LMSG_03116_at | class I glutamine amidotransferase/Pfam=PF00425.10 |
| LMSG_03117_at | conserved hypothetical protein |
| LMSG_03118_at | conserved hypothetical protein |
| LMSG_03119_at | predicted protein/Pfam=PF01817.13 |
| LMSG_03159_at | conserved hypothetical protein/Pfam=PF03382.6 |
| LMSG_03160_at | predicted protein |
| LMSG_03162_at | phage protein |
| LMSG_03163_at | phage protein |
| LMSG_03164_x_at | conserved hypothetical protein |

NK: Gene function not known as predicted by Gene Locator and Interpolated Markov ModelER 3 (Glimmer3)
