# Supplementary material for: High Density Microarray Analysis Reveals New Insights into Genetic Footprints of Listeria monocytogenes Strains Involved in Listeriosis Outbreaks
Source: PLoS One. 2012 Mar 21;7(3):e32896. doi: 10.1371/journal.pone.0032896 (PMC3310058; doi:10.1371/journal.pone.0032896)
Supplement: Table S8 — Probe-sets uniquely present in the serotype 4b, epidemic clone V. (DOCX) [file pone.0032896.s008.docx]

**Supporting Information Table S8: Probe-sets uniquely present in the serotype 4b, epidemic clone V**

| **Probe ID** | **Annotation** |
| --- | --- |
| AARI_0276_x_at | 100% similar to lmo2752 |
| AARL_0363_x_at | NK |
| AARL_0416_at | 99% similar to LMHCC_0209 |
| AARL_0844_x_at | NK |
| AARM_0199_at | 100% similar to lmo0333 |
| AARM_1025_x_at | 99% similar to lmo0560 |
| AARO_0419_at | 98% similar to LMOf2365_1509 |
| AARO_1499_at | 98% similar to LMOf2365_2739 |
| AARY_1162_s_at | 100% similar to lmo2389 |
| IGLm4b_02049_at | Intergenic region |
| IGLMHCC_0202_x_at | Intergenic region |
| IGLMHCC_0203_at | Intergenic region |
| IGlmo0102_x_at | Intergenic region |
| IGlmo0466_at | Intergenic region |
| IGlmo2181_x_at | Intergenic region |
| IGlmo2456_x_at | Intergenic region |
| Lm4b_02686_s_at | Putative gluconate kinase/GI=225877725 |
| LMBG_01285_s_at | diphosphomevalonate decarboxylase/Pfam=PF08544.5 |
| LMBG_02701_s_at | copper resistance domaincontaining protein/Pfam=PF05425.5 |
| LMFG_01232_at | conserved hypothetical protein |
| LMFG_01233_at | conserved hypothetical protein |
| LMFG_01234_at | DNA adenine methylase/Pfam=PF02086.7 |
| LMFG_02006_x_at | homoserine Oacetyltransferase/Pfam=PF00561.12 |
| LMFG_02913_x_at | major facilitator family transporter/Pfam=PF07690.8 |
| LMHCC_0195_s_at | SerThr protein phosphatase family protein/GI=217332763 |
| LMHCC_0199_x_at | CBS domain protein/GI=217332767 |
| LMHCC_0202_at | cell wall surface anchor family protein/GI=217332770 |
| LMHCC_0207_at | NADH dehydrogenase ndh/GI=217332775 |
| LMHCC_0207_x_at | NADH dehydrogenase ndh/GI=217332775 |
| LMHCC_0209_s_at | conserved hypothetical protein/GI=217332777 |
| LMHCC_0599_s_at | ferrichrome transport system permease protein FhuG/GI=217333161 |
| LMHCC_0649_s_at | MazG nucleotide pyrophosphohydrolase/GI=217333210 |
| LMHCC_0920_s_at | conserved hypothetical protein/GI=217333475 |
| LMHCC_1265_s_at | tkt transketolase/GI=217333818 |
| LMHCC_1487_s_at | PduO/GI=217334037 |
| LMHCC_1767_s_at | sugar ABC transporter permease protein/GI=217334314 |
| LMHCC_2299_s_at | conserved hypothetical protein/GI=217334843 |
| LMHCC_2463_at | sugar ABC transporter, permease protein/GI=217335004 |
| LMHCC_2463_x_at | sugar ABC transporter, permease protein/GI=217335004 |
| LMHG_00319_x_at | conserved hypothetical protein/Pfam=PF00923.11 |
| LMHG_00428_x_at | multidrug efflux transporter transcriptional regulatory protein/Pfam=PF00376.15 |
| LMHG_01337_x_at | conserved hypothetical protein |
| LMHG_01345_x_at | CBS domaincontaining protein/Pfam=PF03471.9 |
| LMIG_02954_x_at | conserved hypothetical protein |
| LMKG_01430_s_at | conserved hypothetical protein |
| LMKG_02150_x_at | predicted protein |
| LMKG_02749_x_at | cell wall surface anchor family protein/Pfam=PF06458.4 |
| LMMG_03068_x_at | conserved hypothetical protein |
| lmo0078_x_at | GI=16409437 |
| lmo0466_at | GI=16409843 |
| lmo2394_x_at | GI=16411882 |
| LMOf6854_0339_at | site-specific recombinase, resolvase family/GI=47015209 |
| LMOG_03190_x_at | conserved hypothetical protein |
| LMOG_03191_x_at | conserved hypothetical protein |
| LMRG_00028_x_at | conserved hypothetical protein |
| LMRG_00650_x_at | cbiM/Pfam=PF01891.8 |

NK: Gene function not known as predicted by Gene Locator and Interpolated Markov ModelER 3 (Glimmer3)
