# Supplementary material for: High Density Microarray Analysis Reveals New Insights into Genetic Footprints of Listeria monocytogenes Strains Involved in Listeriosis Outbreaks
Source: PLoS One. 2012 Mar 21;7(3):e32896. doi: 10.1371/journal.pone.0032896 (PMC3310058; doi:10.1371/journal.pone.0032896)
Supplement: Table S9 — Probe-sets uniquely present in the serotype 1/2b strains that cause invasive listeriosis. (DOCX) [file pone.0032896.s009.docx]

**Supporting Information Table S9: Probe-sets uniquely present in the serotype 1/2b strains that cause invasive listeriosis**

| **Probe ID** | **Annotation** |
| --- | --- |
| AARI_0276_x_at | 100% similar to lmo2752 |
| AARM_0103_x_at | NK |
| AARM_1126_s_at | NK |
| AARO_0167_x_at | 99% similar to LMOf2365_2666 |
| AARO_0419_at | 98% similar to LMOf2365_1509 |
| IGLMHCC_0686_at | Intergenic region |
| IGLMHCC_2114_at | Intergenic region |
| IGlmo2686_at | Intergenic region |
| IGLMOf2365_1757_x_at | Intergenic region |
| IGLMOf2365_2051_at | Intergenic region |
| IGLMOf2365_2051_x_at | Intergenic region |
| IGLMOf2365_2055_x_at | Intergenic region |
| IGLMOf2365_2756_x_at | Intergenic region |
| lmo2686_s_at | GI=16412186 |
| LMOf2365_0547_x_at | putative membrane protein/GI=46880028 |
| LMOf6854_2802_s_at | hypothetical protein/GI=47016100 |
| LMOG_01819_s_at | predicted protein |
| LMOG_01819_x_at | predicted protein |
| LMSG_01256_x_at | conserved hypothetical protein/Pfam=PF03992.8 |

NK: unknown function gene as predicted by Gene Locator and Interpolated Markov ModelER 3 (Glimmer3)
