# Supplementary material for: High Density Microarray Analysis Reveals New Insights into Genetic Footprints of Listeria monocytogenes Strains Involved in Listeriosis Outbreaks
Source: PLoS One. 2012 Mar 21;7(3):e32896. doi: 10.1371/journal.pone.0032896 (PMC3310058; doi:10.1371/journal.pone.0032896)
Supplement: Table S10 — Probe-sets uniquely present in the serotype 1/2b strains that cause febrile gastroenteritis listeriosis. (DOCX) [file pone.0032896.s010.docx]

**Supporting Information Table S10: Probe-sets uniquely present in the serotype 1/2b strains that cause febrile gastroenteritis listeriosis**

| **Probe ID** | **Annotation** |
| --- | --- |
| AARM_0148_s_at | 98% similar to lmo2752 |
| AARO_0403_at | NK |
| AARY_0504_at | NK |
| IGLm4b_01609_s_at | Intergenic region |
| IGLm4b_02741_x_at | Intergenic region |
| IGLMHCC_1069_at | Intergenic region |
| IGLMHCC_1408_at | Intergenic region |
| IGLMHCC_2168_x_at | Intergenic region |
| IGLMHCC_2758_at | Intergenic region |
| IGlmo1597_at | Intergenic region |
| IGlmo1598_x_at | Intergenic region |
| IGLMOf2365_1619_at | Intergenic region |
| IGLMOf2365_2056_s_at | Intergenic region |
| IGLMOf2365_2631_at | Intergenic region |
| IGLMOf2365_2632_s_at | Intergenic region |
| Lm4b_01608_at | Hypothetical protein of unknown function/GI=225876660 |
| Lm4b_01730_x_at | Putative transcription regulator/GI=225876781 |
| LMBG_00856_at | conserved hypothetical protein |
| LMBG_02554_x_at | transcriptional regulator/Pfam=PF00440.15 |
| LMFG_00327_at | predicted protein |
| LMFG_00327_x_at | predicted protein |
| LMFG_01059_at | predicted protein |
| LMFG_01842_at | conserved hypothetical protein/Pfam=PF06993.4 |
| LMFG_01842_x_at | conserved hypothetical protein/Pfam=PF06993.4 |
| LMFG_01843_at | drug resistance transporter/Pfam=PF07690.8 |
| LMFG_01866_at | phage protein |
| LMFG_02839_at | conserved hypothetical protein |
| LMFG_02954_at | phage protein |
| LMFG_02955_at | phage protein |
| LMFG_02973_s_at | phage protein |
| LMFG_03063_at | 2oxoisovalerate dehydrogenase E1 component/Pfam=PF02780.12 |
| LMFG_03063_x_at | 2oxoisovalerate dehydrogenase E1 component/Pfam=PF02780.12 |
| LMHCC_1370_at | gp8/GI=217333921 |
| LMHCC_1408_s_at | conserved hypothetical protein/GI=217333959 |
| LMHCC_2171_s_at | hydantoinaseoxoprolinase family protein/GI=217334715 |
| LMHCC_2702_x_at | aminoacylase/GI=217335243 |
| LMHG_00285_at | transcriptional regulator/Pfam=PF01047.14 |
| LMJG_02806_s_at | predicted protein |
| LMJG_02961_x_at | conserved hypothetical protein |
| LMJG_03009_at | conserved hypothetical protein |
| LMMG_03068_x_at | conserved hypothetical protein |
| lmo1597_s_at | GI=16411026 |
| lmo2275_s_at | Portein gp28 [Bacteriophage A118]/GI=16411745 |
| lmo2326_s_at | GI=16411815 |
| LMOf2365_2626_s_at | hypothetical protein/GI=46882098 |
| LMOf2365_2627_at | ABC transporter, ATP-binding protein/GI=46882099 |
| LMOf2365_2628_at | putative membrane protein/GI=46882100 |
| LMOf2365_2629_s_at | putative membrane protein/GI=46882101 |
| LMOf2365_2630_s_at | putative membrane protein/GI=46882102 |
| LMOf2365_2631_s_at | conserved hypothetical protein/GI=46882103 |
| LMOf6854_1649_s_at | conserved hypothetical protein/GI=47013861 |
| LMOf6854_2656_at | holin, phage phi LC3 family/GI=47014863 |
| LMOf6854_2657_s_at | conserved hypothetical protein/GI=47014864 |
| LMOf6854_2669_s_at | conserved hypothetical protein/GI=47014316 |
| LMOf6854_2670_s_at | main capsid protein Gp34/GI=47014317 |
| LMOf6854_2673_s_at | conserved hypothetical protein/GI=47014320 |
| LMOh7858_1159_at | PTS system, IIB component, putative/GI=47019607 |
| LMOh7858_2437_s_at | conserved hypothetical protein/GI=47019178 |

NK: unknown function gene as predicted by Gene Locator and Interpolated Markov ModelER 3 (Glimmer3)
