# Supplementary material for: High Density Microarray Analysis Reveals New Insights into Genetic Footprints of Listeria monocytogenes Strains Involved in Listeriosis Outbreaks
Source: PLoS One. 2012 Mar 21;7(3):e32896. doi: 10.1371/journal.pone.0032896 (PMC3310058; doi:10.1371/journal.pone.0032896)
Supplement: Table S11 — Probe-sets uniquely present in the serotype 4b, ECIV strains that cause invasive listeriosis. (DOCX) [file pone.0032896.s011.docx]

**Supporting Information Table S11: Probe-sets uniquely present in the serotype 4b, ECIV strains that cause invasive listeriosis**

| **Probe ID** | **Annotation** |
| --- | --- |
| AARL_0858_at | NK |
| IGlmo0903_x_at | Intergenic region |
| IGLMOf2365_1690_x_at | Intergenic region |
| LMHCC_2317_x_at | thiM hydroxyethylthiazole kinase/GI=217334860 |
| LMIG_02904_s_at | parC/Pfam=PF00521.12 |
| LMKG_01035_at | phage protein |
| LMMG_03022_x_at | NK |
| lmo1556_s_at | hemC GI=16410985 |
| LMOh7858_0273_x_at | conserved hypothetical protein/GI=47017012 |
| LMRG_00513_x_at | NK |

NK: unknown function gene as predicted by Gene Locator and Interpolated Markov ModelER 3 (Glimmer3)
