# Supplementary material for: High Density Microarray Analysis Reveals New Insights into Genetic Footprints of Listeria monocytogenes Strains Involved in Listeriosis Outbreaks
Source: PLoS One. 2012 Mar 21;7(3):e32896. doi: 10.1371/journal.pone.0032896 (PMC3310058; doi:10.1371/journal.pone.0032896)
Supplement: Table S12 — Probe-sets uniquely present in the serotype 4b, ECIV strains that cause febrile gastroenteritis listeriosis. (DOCX) [file pone.0032896.s012.docx]

**Supporting Information Table S12: Probe-sets uniquely present in the serotype 4b, ECIV strains that cause febrile gastroenteritis listeriosis**

| **Probe ID** | **Annotation** |
| --- | --- |
| AARI_0400_at | NK |
| AARM_0169_at | NK |
| AARO_0228_s_at | NK |
| IGLMHCC_2939_at | Intergenic region |
| IGLMHCC_3005_at | Intergenic region |
| IGlmo2321_at | Intergenic region |
| IGlmo2329_at | Intergenic region |
| LMBG_01649_s_at | predicted protein |
| LMBG_01659_s_at | TerL/Pfam=PF07570.4 |
| LMFG_02488_at | phage protein/Pfam=PF00589.14 |
| LMFG_02489_at | phage protein |
| LMFG_02490_at | phage protein/Pfam=PF06114.5 |
| LMFG_02491_at | phage protein/Pfam=PF01381.14 |
| LMFG_02492_at | phage protein/Pfam=PF01381.14 |
| LMFG_02493_at | conserved hypothetical protein |
| LMFG_02494_at | phage protein |
| LMFG_02495_at | phage protein |
| LMFG_02496_at | phage protein |
| LMFG_02497_at | phage protein |
| LMFG_02498_at | phage protein |
| LMFG_02499_at | phage protein |
| LMFG_02500_at | phage protein/Pfam=PF03837.6 |
| LMFG_02507_at | phage protein/Pfam=PF05866.3 |
| LMFG_02510_at | phage protein |
| LMFG_02519_at | phage protein |
| LMFG_02523_at | phage protein |
| LMFG_02524_at | phage protein/Pfam=PF09669.2 |
| LMFG_02525_at | phage protein |
| LMFG_02525_x_at | phage protein |
| LMFG_02531_at | phage protein |
| LMFG_02535_at | phage protein/Pfam=PF09714.2 |
| LMFG_02538_at | phage protein |
| LMFG_02541_at | phage protein |
| LMFG_02542_at | phage protein |
| LMFG_02543_at | phage protein |
| LMFG_02544_at | phage protein |
| LMFG_02545_at | phage protein |
| LMFG_02546_at | phage protein |
| LMFG_02547_at | phage protein |
| LMFG_02548_at | phage protein |
| LMFG_02549_at | phage protein |
| LMFG_02550_at | phage tail tape measure protein/Pfam=PF01551.14 |
| LMFG_02551_at | phage protein/Pfam=PF01476.12 |
| LMFG_02552_at | phage protein |
| LMFG_02553_at | phage protein |
| LMFG_02554_at | phage protein |
| LMFG_02555_at | phage protein |
| LMFG_02561_at | phage protein |
| LMFG_02562_at | phage protein |
| LMFG_02563_at | phage protein/Pfam=PF01476.12 |
| LMFG_02566_at | phage protein/Pfam=PF01381.14 |
| LMFG_02567_at | phage protein |
| LMFG_02568_at | phage protein |
| LMFG_02569_at | phage protein |
| LMFG_02839_at | conserved hypothetical protein |
| LMFG_02965_s_at | TerS |
| LMFG_02966_s_at | TerL/Pfam=PF03237.7 |
| LMFG_03196_x_at | phage protein |
| LMFG_03215_s_at | conserved hypothetical protein/Pfam=PF06356.3 |
| LMHCC_1395_s_at | alkylphosphonate utilization operon protein PhnA/GI=217333946 |
| LMHCC_1401_s_at | conserved hypothetical protein/GI=217333952 |
| LMHCC_2938_at | rpsI ribosomal protein S9/GI=217335475 |
| LMHCC_2939_s_at | integrase/GI=217335476 |
| LMHCC_2941_s_at | gp15/GI=217335478 |
| LMHCC_2950_s_at | gp43/GI=217335487 |
| LMHCC_2951_s_at | conserved hypothetical protein/GI=217335488 |
| LMHCC_2979_s_at | Phage terminase, small subunit/GI=217335516 |
| LMHCC_2992_at | gp15/GI=217335529 |
| LMJG_02942_s_at | predicted protein/Pfam=PF01381.14 |
| LMJG_02960_s_at | phage protein |
| LMKG_00015_s_at | phage protein/Pfam=PF06152.3 |
| LMMG_02721_s_at | conserved hypothetical protein |
| lmo2294_s_at | Protein gp9 [Bacteriophage A118]/GI=16411764 |
| lmo2328_at | GI=16411817 |
| lmo2328_s_at | GI=16411817 |
| lmo2329_s_at | GI=16411818 |
| LMOf6854_2366_s_at | positive control factor, putative/GI=47015603 |
| LMOf6854_2663_s_at | conserved hypothetical protein/GI=47014310 |
| LMOf6854_2666_s_at | conserved hypothetical protein/GI=47014313 |
| LMOf6854_2667_s_at | hypothetical protein/GI=47014314 |
| LMOf6854_2668_s_at | conserved hypothetical protein/GI=47014315 |
| LMOf6854_2671_s_at | conserved hypothetical protein/GI=47014318 |
| LMOf6854_2699_s_at | transcriptional regulator, CroCI family/GI=47014066 |
| LMOf6854_2700_s_at | conserved hypothetical protein/GI=47014067 |
| LMOG_03115_s_at | phage protein/Pfam=PF05866.3 |
| LMOG_03116_s_at | phage protein |
| LMOG_03116_x_at | phage protein |
| LMOh7858_2418_at | Gp16 protein/GI=47019159 |
| LMOh7858_2419_at | gp20/GI=47019160 |
| LMOh7858_2421_at | phage minor structural protein, N-terminal region subfamily/GI=47019162 |
| LMOh7858_2422_s_at | holin-like protein, putative/GI=47019163 |
| LMOh7858_2434_s_at | conserved hypothetical protein/GI=47019175 |
| LMOh7858_2437_s_at | conserved hypothetical protein/GI=47019178 |
| LMOh7858_2439_s_at | hypothetical gene lin2398 -related protein/GI=47019180 |
| LMOh7858_2439_x_at | hypothetical gene lin2398 -related protein/GI=47019180 |
| LMOh7858_2448_s_at | Gp32 protein/GI=47019188 |
| LMOh7858_2453_s_at | recC/GI=47019193 |
| LMOh7858_2475_s_at | site-specific recombinase, resolvase family, putative/GI=47019208 |
| LMRG_01513_at | predicted protein |
| LMRG_01521_s_at | phage protein |
| LMRG_01532_x_at | TerL/Pfam=PF07570.4 |
| LMRG_02920_s_at | phage protein/Pfam=PF08346.4 |
| LMRG_02920_x_at | phage protein/Pfam=PF08346.4 |
| LMSG_00001_at | phage protein/Pfam=PF07852.3 |
| LMSG_00003_at | phage protein/Pfam=PF09681.2 |
| LMSG_00007_at | conserved hypothetical protein |
| LMSG_00007_x_at | conserved hypothetical protein |
| LMSG_00010_at | predicted protein |
| LMSG_00011_at | predicted protein/Pfam=PF01381.14 |
| LMSG_00011_x_at | predicted protein/Pfam=PF01381.14 |
| LMSG_00012_at | transcriptional regulator/Pfam=PF01381.14 |
| LMSG_00014_at | conserved hypothetical protein |
| LMSG_00015_at | integrase/Pfam=PF00589.14 |
| LMSG_02265_at | comK |
| LMSG_02266_at | conserved hypothetical protein |
| LMSG_02267_x_at | phage protein |
| LMSG_02515_at | transcriptional regulator |
| LMSG_02518_at | conserved hypothetical protein/Pfam=PF07508.5 |
| LMSG_02519_at | ComK/Pfam=PF06338.3 |
| LMSG_02787_at | phage protein |
| LMSG_02793_at | phage protein/Pfam=PF05565.3 |
| LMSG_02794_at | phage protein |
| LMSG_02795_at | phage protein |
| LMSG_02796_at | phage protein |
| LMSG_02797_at | predicted protein |
| LMSG_02798_at | conserved hypothetical protein |
| LMSG_02799_at | phage protein |
| LMSG_02799_s_at | phage protein |
| LMSG_02802_at | conserved hypothetical protein |
| LMSG_02805_at | phage protein/Pfam=PF02368.10 |
| LMSG_02807_at | phage protein |
| LMSG_02807_s_at | phage protein |
| LMSG_02808_at | phage protein |
| LMSG_02808_x_at | phage protein |
| LMSG_02809_at | phage protein |
| LMSG_02810_at | predicted protein |
| LMSG_02810_x_at | predicted protein |
| LMSG_02811_at | main capsid protein |
| LMSG_02814_at | phage protein |
| LMSG_02814_x_at | phage protein |
| LMSG_02815_at | TerL/Pfam=PF03237.7 |
| LMSG_02818_at | conserved hypothetical protein/Pfam=PF08281.4 |
| LMSG_02819_x_at | conserved hypothetical protein |
| LMSG_02820_at | phage protein |
| LMSG_02823_at | helixdestabilizing protein 2 |
| LMSG_02823_x_at | helixdestabilizing protein 2 |
| LMSG_02926_at | TerS/Pfam=PF03592.8 |
| LMSG_02926_x_at | TerS/Pfam=PF03592.8 |
| LMSG_02927_at | phage protein |
| LMSG_02928_at | conserved hypothetical protein/Pfam=PF08281.4 |
| LMSG_02928_x_at | conserved hypothetical protein/Pfam=PF08281.4 |
| LMSG_02929_at | phage protein/Pfam=PF05866.3 |
| LMSG_02930_at | phage protein |
| LMSG_02931_at | singlestrand binding protein/Pfam=PF00436.17 |
| LMSG_02931_x_at | singlestrand binding protein/Pfam=PF00436.17 |
| LMSG_02932_at | conserved hypothetical protein |
| LMSG_02965_x_at | phage protein |
| LMSG_02987_at | phage protein |
| LMSG_02995_at | conserved hypothetical protein |
| LMSG_02996_x_at | phage protein |
| LMSG_03002_s_at | recombinase/Pfam=PF03837.6 |
| LMSG_03007_s_at | major tail shaft protein |
| LMSG_03007_x_at | major tail shaft protein |
| LMSG_03035_at | transcriptional regulator/Pfam=PF00440.15 |
| LMSG_03036_at | small multidrug resistance protein/Pfam=PF00893.11 |
| LMSG_03037_at | transposase C |
| LMSG_03038_at | transposase B/Pfam=PF00589.14 |
| LMSG_03039_at | transposase A/Pfam=PF02899.9 |
| LMSG_03040_at | DNA repair protein RadC/Pfam=PF04002.6 |
| LMSG_03123_at | predicted protein |
| LMSG_03124_at | predicted protein |
| LMSG_03134_s_at | phage protein |
| LMSG_03134_x_at | phage protein |
| LMSG_03143_at | conserved hypothetical protein |
| LMSG_03144_at | phage protein |
| LMSG_03144_x_at | phage protein |
| LMSG_03147_at | phage protein |
| LMSG_03148_at | XlyB/Pfam=PF01510.17 |
| LMSG_03149_at | conserved hypothetical protein |
| LMSG_03149_x_at | conserved hypothetical protein |
| LMSG_03152_at | phage protein/Pfam=PF03374.6 |
| LMSG_03153_x_at | conserved hypothetical protein |
| LMSG_03154_x_at | phage protein |
| LMSG_03156_s_at | conserved hypothetical protein |
| LMSG_03158_s_at | phage protein |
| LMSG_03158_x_at | phage protein |
| LMSG_03166_at | phage protein |
| LMSG_03167_s_at | predicted protein |
| LMSG_03168_x_at | predicted protein |
| LMSG_03170_at | phage protein |
| LMSG_03171_s_at | phage protein |
| LMSG_03176_at | phage protein/Pfam=PF03374.6 |
| LMSG_03176_x_at | phage protein/Pfam=PF03374.6 |

NK: unknown function gene as predicted by Gene Locator and Interpolated Markov ModelER 3 (Glimmer3)
